# A DNA barcode library for ground beetles (Insecta, Coleoptera, Carabidae) of Germany: The genus *Bembidion* Latreille, 1802 and allied taxa

**DOI:** 10.3897/zookeys.592.8316

**Published:** 2016-05-25

**Authors:** Michael J. Raupach, Karsten Hannig, Jérome Morinière, Lars Hendrich

**Affiliations:** 1 Molecular Taxonomy of Marine Organisms, German Centre of Marine Biodiversity Research (DZMB), Senckenberg am Meer, Südstrand 44, 26382 Wilhelmshaven, Germany; 2 Bismarckstraße 5, 45731 Waltrop, Germany; 3 Taxonomic coordinator – Barcoding Fauna Bavarica, Bavarian State Collection of Zoology (SNSB – ZSM), Münchhausenstraße 21, 81247 München, Germany; 4 Sektion Insecta varia, Bavarian State Collection of Zoology (SNSB – ZSM), Münchhausenstraße 21, 81247 München, Germany

**Keywords:** Asaphidion, Central Europe, cytochrome *c* oxidase subunit I, German Barcode of Life, mitochondrial DNA, molecular specimen identification, Ocys, Sinechostictus

## Abstract

As molecular identification method, DNA barcoding based on partial cytochrome *c* oxidase subunit 1 (COI) sequences has been proven to be a useful tool for species determination in many insect taxa including ground beetles. In this study we tested the effectiveness of DNA barcodes to discriminate species of the ground beetle genus *Bembidion* and some closely related taxa of Germany. DNA barcodes were obtained from 819 individuals and 78 species, including sequences from previous studies as well as more than 300 new generated DNA barcodes. We found a 1:1 correspondence between BIN and traditionally recognized species for 69 species (89%). Low interspecific distances with maximum pairwise K2P values below 2.2% were found for three species pairs, including two species pairs with haplotype sharing (*Bembidion atrocaeruleum*/*Bembidion varicolor* and *Bembidion guttula*/*Bembidion mannerheimii*). In contrast to this, deep intraspecific sequence divergences with distinct lineages were revealed for two species (*Bembidion geniculatum*/*Ocys harpaloides*). Our study emphasizes the use of DNA barcodes for the identification of the analyzed ground beetles species and represents an important step in building-up a comprehensive barcode library for the Carabidae in Germany and Central Europe as well.

## Introduction

The Carabidae (ground beetles) is a large cosmopolitan family of the Coleoptera, with an estimated number of 40,000 species world-wide, about 2,700 in Europe and 567 in Germany (Arnett et al. 2000, Arndt et al. 2005, Luff 2007, Trautner et al. 2014). Their body is usually rather flattened, especially in species living in crevices in soil such as some species of *Bembidion* Latreille, 1802, *Pterostichus* Bonelli, 1810, and *Polistichus* Bonelli, 1810, or under bark as in some *Dromius* Bonelli, 1810 species (Luff 2007). Although the majority of ground beetles are dark-colored and often black, there are many exceptions to this general rule, for example various colorful species of the genera *Anchomenus* Bonelli, 1810, *Carabus* Linnaeus, 1758, *Ceroglossus* Solier, 1848, or *Lebia* Latreille, 1802. Most ground beetles are active terrestrial beetles which forage on the ground surface and prey on other small invertebrates. Carabid beetles show, however, different levels of habitat selectivity, ranging from generalists to specialists. As consequence, carabid assemblages can be used as highly valuable bioindicators for characterizing disturbances in various habitats such as forests, meadows or fens (Lövei and Sunderland 1996, Rainio and Niemelä 2003, Pearce and Venier 2004, Koivula 2011, Kotze et al. 2011).

**Figure 1.**
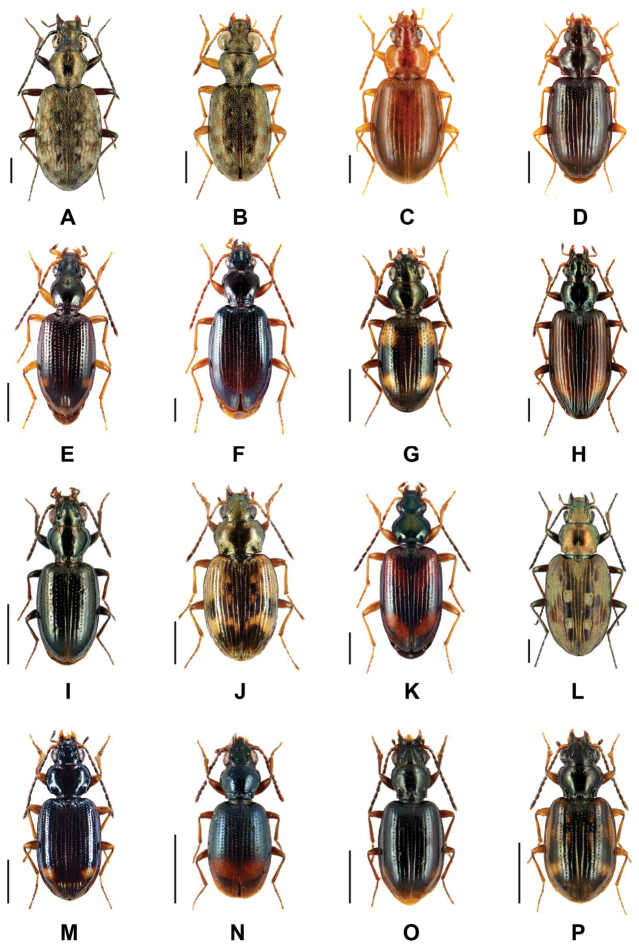
Representative images of analyzed beetle species. **A** *Asaphidion caraboides* (Schrank, 1781) **B** *Asaphidion flavipes* (Linnaeus, 1761) **C** *Ocys harpaloides* (Audinet-Serville, 1821) **D** *Ocys quinquestriatus* (Gyllenhal, 1810) **E** *Sinechostictus elongatus* (Dejean, 1831) **F** *Sinechostictus ruficornis* (Sturm, 1825) **G** Bembidion (Bembidion) quadrimaculatum (Linnaeus, 1761) **H** Bembidion (Bembidionetolitzkya) fasciolatum (Duftschmid, 1812) **I** Bembidion (Emphanes) azurescens Dalla Torre, 1877 **J** Bembidion (Notaphus) semipunctatum (Donovan, 1806) **K** Bembidion (Ocydromus) testaceum (Duftschmid, 1812) **L** Bembidion (Bracteon) litorale (Olivier, 1790) **M** Bembidion (Philochthus) biguttatum (Fabricius, 1779) **N** Bembidion (Talanes) aspericolle (Germar, 1829) **O** Bembidion (Trepanedoris) doris (Panzer, 1796), and **P** Bembidion (Trepanes) octomaculatum (Goeze, 1777). Scale bars = 1 mm. All images were obtained from www.eurocarabidae.de.

Within the Carabidae, the genus *Bembidion* Latreille, 1802 is the largest in this family, with more than 1,200 described species mostly in the temperate regions of the world (Maddison 2012), including about 220 in Europe (Luff 2007) and more than 80 in Germany (Trautner et al. 2014). Species of *Bembidion* are typically small predators that inhabit shores of running or standing waters including coastlines in temperate regions. Most adults have a body length between 2 and 9 mm (Lindroth 1985). Typically, species of this genus vary in the form of the prothorax and elytra, microsculpture, color pattern, mouthparts, male genitalia, and other characters (Maddison 2012) (Fig. 1). However, a study of males is often indispensable for the identification of morphologically similar species. Moreover, the identification of larvae is even more difficult due to a lack of documentation as well as missing experts with relevant skills and reference material.

During the last years the analysis of DNA sequence data, in particular the use of an approx. 650 base pair (bp) fragment of the mitochondrial cytochrome *c* oxidase subunit 1 (COI) known as DNA barcode, was proposed as marker of choice for specimen identification (Hebert et al. 2003a, Hebert et al. 2003b). The idea of DNA barcoding is based on the assumption that the observed interspecific genetic variation exceeds the intraspecific variation to such proportion that a clear gap exists, allowing the assignment of unidentified individuals to their species (Hebert et al. 2003a, Hebert et al. 2003b). Thus, the compilation of comprehensive DNA barcode libraries represents an essential step for subsequent studies, e.g. biodiversity assessment studies via metabarcoding using high-throughput sequencing technologies in the near future (e.g. Zhou et al. 2013, Cristescu 2014, Brandon-Mong et al. 2015).

However, the application of COI (and other mitochondrial markers in general) for species identification is not without problems. Recent speciation and hybridization events (e.g. Kubota and Sota 1998, Sota et al. 2000), heteroplasmy (e.g. Boyce et al. 1989), the presence of mitochondrial pseudogenes (numts; e.g. Hazakani-Covo et al. 2010, Maddison 2012), or incomplete lineage sorting as consequence of occasional complex phylogeographic processes (e.g. Petit and Excoffier 2009) can influence the mitochondrial variability of the barcode fragment. In the case of terrestrial arthropods and in particular insects, maternally inherited α-proteobacteriae as *Wolbachia* Hertig, 1936 can limit the application of DNA barcodes for valid species identification also (e.g. Dobson 2004, Duron et al. 2008, Werren et al. 2008). It is also possible to generate *Wolbachia* COI sequences using standard insect primers (Smith et al. 2012). Finally, other studies highlight methodological problems of the analysis of DNA barcodes, for example an inappropriate use of neighbor-joining trees or of fixed distance thresholds (e.g. Will and Rubinoff 2004, Goldstein and DeSalle 2010, Collins and Cruickshank 2013). Nevertheless, numerous studies clearly demonstrate the usefulness of DNA barcoding for vertebrates (e.g. Lijtmaer et al. 2011, Ivanova et al. 2012, Knebelsberger et al. 2014) as well as invertebrates (e.g. Costa et al. 2007, Lobo et al. 2015, Barco et al. 2016). Not surprisingly, most DNA barcoding studies of arthropods focus on insects (Raupach and Radulovici 2015). In this context, numerous sequence libraries have been build-up for a broad range of insect taxa, including Heteroptera (Jung et al. 2011, Park et al. 2011, Grebennikov and Heiss 2014, Raupach et al. 2014), Neuroptera (Morinière et al. 2014), Hymenoptera (e.g. Smith and Fisher 2009, Quicke et al. 2012, Schmidt et al. 2015), Trichoptera (Zhou et al. 2009, Zhou et al. 2011, Ruiter et al. 2013), and in particular Lepidoptera (e.g. deWaard et al. 2009, Dincǎ et al. 2011, Hausmann et al. 2011, Hebert et al. 2013, Rajaei Sh et al. 2013, Kekkonen et al. 2015). Beside various other articles analyzing Coleoptera by the means of DNA barcoding (e.g. Hendrich et al. 2010, Greenstone et al. 2011, Jusoh et al. 2014, Pentinsaari et al. 2014, Oba et al. 2015, Rougerie et al. 2015), an amazingly large DNA barcode library of beetles has been published just recently (Hendrich et al. 2015). However, the number of barcoding studies focusing specifically on ground beetles is still low (e.g. Greenstone et al. 2005, Maddison 2008, Raupach et al. 2010, Raupach et al. 2011, Woodcock et al. 2013).

In this study we present as part of the German Barcode of Life project a comprehensive DNA barcode library of a variety of Central European species of the genus *Bembidion* and associated taxa. Our new barcode library includes 65 species of the genus *Bembidion* as well as five species of the closely related genera *Asaphidion* Des Gozis, 1886, two species of the genus *Ocys* Stephens, 1828 and six species of the genus *Sinechostictus* Motschulsky, 1864. In total, our library comprised 819 sequences of 78 species.

## Material and methods

### Sampling of specimens

All analyzed ground beetles were collected between 1997 and 2015 using various sampling methods (i.e. hand collecting, pitfall traps). All specimens were stored in ethanol (96%). The analyzed beetles were identified by one of the authors (KH) using the keys in Müller-Motzfeld (2006). For our analysis we also included 481 DNA barcodes of three previous studies (Raupach et al. 2010: 63 specimens, 11 species; Raupach et al. 2011: 26 specimens, 7 species; Hendrich et al. 2015: 392 specimens, 68 species). In total, 338 new barcodes of 57 species were generated.

Most specimens were collected in Germany (*n* = 617, 75%), but for comparison some specimens were also included from Austria (*n* = 107, 13%), Belgium (*n* = 3, 0.04%), Czech Republic (*n* = 1, 0.01%), Italy (*n* = 41, 0.5%), France (*n* = 34, 0.4%), Slovenia (*n* = 15, 0.2%) and Sweden (*n* = 1, 0.01%). The number of analyzed specimens per species ranged from one (8 species, 10.3%) to a maximum of 38 in the case of *Bembidion tetracolum* Say, 1823.

### DNA barcode amplification, sequencing and data depository

Laboratory operations were carried out either at the Canadian Center for DNA Barcoding (CCDB), University of Guelph, following standardized high-throughput protocols for COI amplification and sequencing (Ivanova et al. 2006, deWaard et al. 2008), the molecular labs of the Zoologisches Forschungsmuseum Alexander Koenig in Bonn, Germany, or the German Center of Marine Biodiversity Research, Senckenberg am Meer, in Wilhelmshaven, Germany. Photographs were taken for each studied beetle before molecular work was performed. For very small specimens with a body length <3 mm, complete specimens were used for DNA extraction, whereas tissue samples (legs) were used for beetles >3 mm. In the case of own molecular studies, DNA was extracted using the QIAmp^©^ Tissue Kit (Qiagen GmbH, Hilden, Germany) or NucleoSpin Tissue Kit (Macherey-Nagel, Düren, Germany), following the extraction protocol.


 Polymerase chain reaction (PCR) has been used for amplifying the COI barcode fragment using the primer pair LCO1480 and HCO2198 (Folmer et al. 1994) or LCO1480 and NANCY (Simon et al. 1994, Simon et al. 2006). The PCR mix contained 4 μl Q-Solution, 2 μl 10x Qiagen PCR buffer, 2 μl dinucleotide triphosphates (dNTPs, 2 mmol/μl), 0.1 μl of each primer (both 25 pmol/μl), 1 μl of DNA template with of between 2 and 150 ng/μl, 0.2 μl Qiagen Taq polymerase (5 U/μl), and was filled up to 20 μl with sterile H_2_O. All PCR amplification reactions were conducted in Thermal Cycler GeneAmp PCR System 2700/2720 (Applied Biosystems, Darmstadt, Germany) or the Eppendorf Mastercycler Pro system (Hamburg, Germany). Thermocycling conditions of the PCR included an initial denaturation at 94 °C (5 min), followed by 38 cycles at 94 °C (denaturation, 45 s), 48 °C (annealing, 45 s), 72 °C (extension, 80 s), and a final extension step at 72 °C (7 min). For each round of reactions negative and positive controls were included. Two μl of the amplified products were verified for size conformity by electrophoresis in a 1% agarose gel stained with GelRed^TM^ and using commercial DNA size standards. The remaining PCR product was purified with the QIAquick^©^ PCR Purification Kit (Qiagen GmbH, Hilden, Germany) or the NucleoSpin Gel and PCR Clean-up (Macherey-Nagel, Düren, Germany). Purified PCR products were cycle-sequenced and sequenced in both directions at contract sequencing facilities (Macrogen, Seoul, Korea, or GATC, Konstanz, Germany), using the same primers as used in PCR. Double stranded sequences were assembled and checked for mitochondrial pseudogenes (numts) analysing the presence of stop codons, frameshifts as well as double peaks in chromatograms with the Geneious version 7.0.4 program package (Biomatters, Auckland, New Zealand) (Kearse et al. 2012). For verification, BLAST searches (nBLAST, search set: others, program selection: megablast) were performed to confirm the identity of all new sequences as beetle sequences based on already published sequences (high identity values, very low E-values) (Zhang et al. 2000, Morgulis et al. 2008). All analyzed sequences had a length of at least 352 base pairs (bp). Relevant voucher information, taxonomic classifications, photos, DNA barcode sequences, used primer pairs and trace files (including their quality) are publicly accessible through the public data set “DS-BABEM” (Dataset ID: doi: 10.5883/DS-BABEM) on the Barcode of Life Data Systems (BOLD; www.boldsystems.org) (Ratnasingham and Hebert 2007). New barcode data were also deposited in GenBank (accession numbers KU876564 to KU876786).

### DNA Barcode analysis

Intra- and interspecific distances of the analyzed ground beetle species were based on the Kimura 2-parameter (K2P; Kimura 1980), using the analytical tools of the BOLD workbench (align sequences: BOLD aligner; ambiguous base/gap handling: pairwise deletion). Beside this, all analyzed COI sequences were subject to the Barcode Index Number (BIN) system implemented in BOLD. This approach clusters DNA barcodes in order to produce operational taxonomic units that closely correspond to species (Ratnasingham and Hebert 2013). Such BINs are unique in that clusters are indexed in a regimented way so genetically identical taxa encountered in different studies reside under shared identifiers (Ratnasingham and Hebert 2013). Using default settings, a recommended threshold of 2.2% was used for a rough differentiation of low and high intraspecific as well as interspecific K2P distances (Ratnasingham and Hebert 2013).

A neighbor joining cluster analysis (NJ; Saitou and Nei 1987) was performed for a graphical representation of the genetic differences between sequences and clusters of sequences in the dataset based on K2P distances using MEGA6.4 (Tamura et al. 2013). Sequences were aligned using MUSCLE (Edgar 2004), implemented in MEGA. Non-parametric bootstrap support values were obtained by resampling and analyzing 1,000 replicates (Felsenstein 1985). Finally, we constructed statistical maximum parsimony networks for species that shared identical haplotypes with TCS 1.21 with a fix connection limit at 50 mutational steps (Clement et al. 2000). Such networks allow the identification of haplotype sharing between species as a consequence of recent speciation or on-going hybridization processes.

## Results

Overall, 819 DNA barcode sequences of 78 carabid beetle species were analyzed. A full list of the analyzed species is presented in the supporting information (Suppl. material 1). For the genus *Bembidion* we analyzed 63 species which represent 77% of the recorded species (*n* = 82) of this genus for Germany. Furthermore, our sampling covered five species (71%) of the genus *Asaphidion* (recorded species for Germany: *n* = 7), two species (100%) of the genus *Ocys* (*n* = 2), and six species (86%) of the genus *Sinechostictus* (*n* = 7). Two analyzed species, *Bembidion dalmatinum* Dejean, 1831 and *Bembidion italicum* De Monte 1943, are actually not recorded from Germany.

Fragment lengths of the analyzed DNA barcode fragments ranged from 352 to 657 bp. As it is typically known for arthropods, our sequence data also revealed a high AT-content for the DNA barcode region: the mean sequence compositions were A=16.6%, C=15.9%, G=29.8% and T=37.7%. Intraspecific K2P distances within a genus ranged from zero to 9.62% (*Ocys harpaloides* (Audinet-Serville, 1821)) whereas interspecific distances within the analyzed genera had values between zero and 14.72%. In this context, the lowest interspecific distances of distinct barcode clusters were revealed for *Bembidion ascendens* K. Daniel, 1902 and *Bembidion fasciolatum* (Duftschmid, 1812) with values ranging from 0.49% to 0.82%. Both species had the same BIN (ACJ7842).

**Figure 2.**
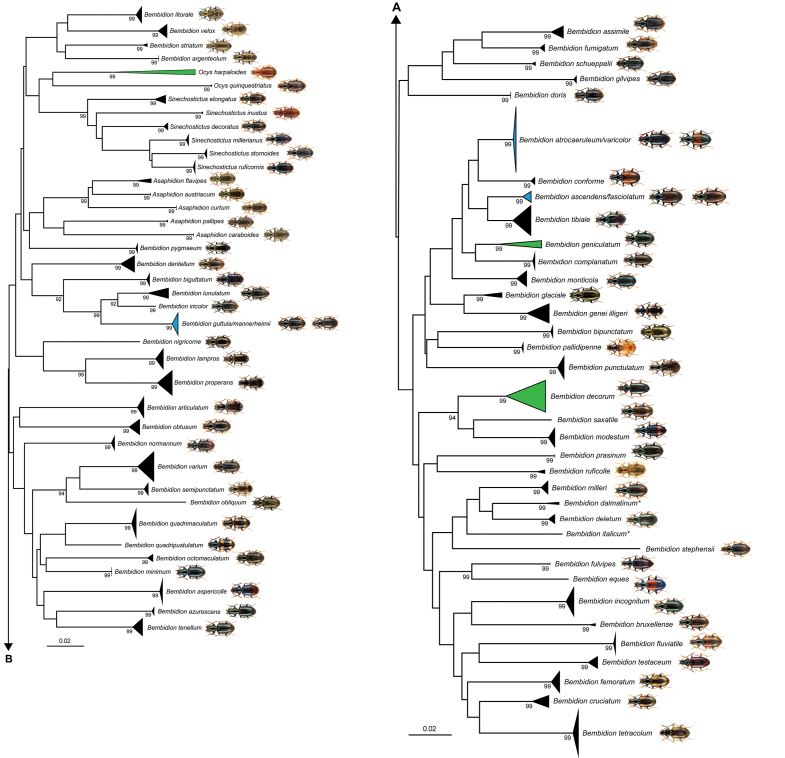
Neighbor joining topology of the analyzed ground beetle species based on Kimura 2-parameter distances. Triangles indicate the relative number of individual’s sampled (height) and sequence divergence (width). Green triangles indicate species with intraspecific maximum pairwise distances >2.2%, blue triangles species pairs with interspecific distances <2.2%. Numbers next to nodes represent non-parametric bootstrap values >90% (1,000 replicates). Asterisks indicate species not recorded in Germany. All images were obtained from www.eurocarabidae.de.

In total, unique BINs were revealed for 69 species (89%), two BINs for 3 species (4%), and one BIN for two species for 6 species (7%). Interspecific distances of zero were found for two species pairs (5.1%): *Bembidion atrocaeruleum* Stephens, 1828 vs. *Bembidion varicolor* Fabricius, 1803 and *Bembidion guttula* (Fabricius, 1792) vs. *Bembidion mannerheimii* C.R. Sahlberg, 1827. In contrast to this, maximum intraspecific pairwise distances >2.2% were found for three species (3.8%): *Bembidion decorum* (Panzer, 1799) (2.56%), *Bembidion geniculatum* Heer, 1837 (4.49%), and *Ocys harpaloides* (Audient-Serville, 1821) (9.62%). The NJ analyses based on K2P distances revealed non-overlapping clusters with bootstrap support values >95% for 63 species (81%) with more than one analyzed specimen (Fig. 2). A detailed topology is presented in the supporting information (Suppl. material 2).

**Figure 3.**
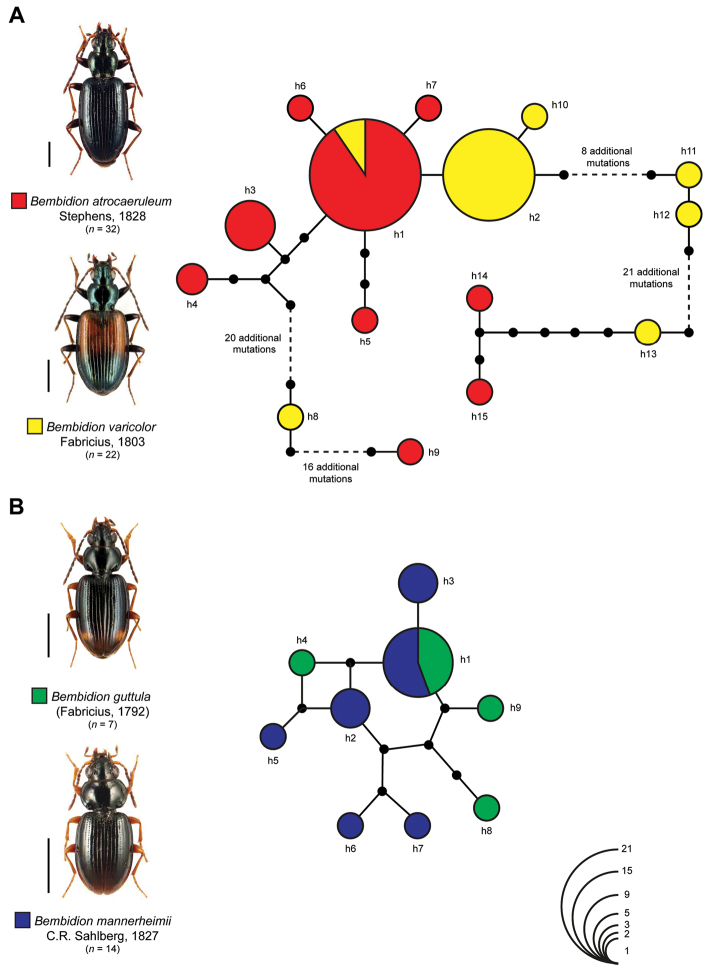
Maximum statistical parsimony network of *Bembidion* species sharing COI haplotypes: **A** *Bembidion atrocaeruleum* Stephens, 1828 and *Bembidion varicolor* Fabricius, 1803 **B** *Bembidion guttula* (Fabricius, 1792) and *Bembidion mannerheimii* C.R. Sahlberg, 1827. Used settings included a user specified maximum of connection steps at 50, gaps were treated as fifth state. Each line represents a single mutational change whereas small black dots indicate missing haplotypes. The numbers of analyzed specimens (*n*) are listed, whereas the diameter of the circles is proportional to the number of haplotypes sampled (see given open half circles with numbers). Scale bars = 1 mm. Beetle images were obtained from www.eurocarabidae.de.

Our statistical maximum parsimony analysis showed multiple sharing of haplotypes for two species pairs: *Bembidion atrocaeruleum* (*n* = 32) and *Bembidion varicolor* (*n* = 22) (Fig. 3A) as well as *Bembidion guttula* (*n* = 7) and *Bembidion mannerheimii* (*n* = 14) (Fig. 3B). For *Bembidion atrocaeruleum* and *Bembidion varicolor* we identified 15 different haplotypes with one dominant haplotype (h1) that was shared by 19 specimens of *Bembidion atrocaeruleum* and two specimens of *Bembidion varicolor*. Interestingly, this haplotype was separated by only one additional mutation step from haplotype h2 which was exclusively composed of specimens of *Bembidion varicolor* (*n* = 15). Whereas a number of haplotypes (h3-h7, h10) was located close to these both major ones, seven haplotypes that were found only within one specimen (*Bembidion atrocaeruleum*: h9, h14, h15; *Bembidion varicolor*: h8, h11, h12, h13) were separated from this core network by high numbers of mutational steps. In the case of *Bembidion guttula* and *Bembidion mannerheimii*, our analysis revealed nine haplotypes. The most dominant haplotype h1 was shared by five specimens of *Bembidion mannerheimii* and four specimens of *Bembidion guttula*. All others were connected to this haplotype by a maximum of five mutational steps, generating a compact network.

## Discussion

Our study clearly confirms the usefulness of DNA barcodes for the identification of species of the genera *Asaphidion*, *Bembidion*, *Ocys*, and *Sinechostictus* of Central Europe, in particular Germany. Unique BINs were found for 69 species (89%) of the analyzed 78 beetle species, coinciding with high rates of successful species identification of previous barcoding studies of ground beetles (Raupach et al. 2010, Raupach et al. 2011, Pentinsaari et al. 2014, Hendrich et al. 2015). Nevertheless, our data also highlights species pairs that share haplotypes as well as species with high genetic diversity and distinct lineages. We will discuss these cases in the following more in detail.

### Species pairs that share haplotypes

Haplotype sharing of COI sequences was found for two species pairs. In the case of *Bembidion guttula* and *Bembidion mannerheimii* identical COI sequences are not surprising (Fig. 3B). A close relationship of both species has been already documented in a previous study (Maddison 2012). In this context our results give some evidence for ongoing hybridization between both species.

A somewhat similar situation was revealed for *Bembidion atrocaeruleum* and *Bembidion varicolor*. Nevertheless, the statistical maximum parsimony network revealed a more complex structure (Fig. 3A). Both species are part of the subgenus *Bembidionetolitzkya* Strand, 1929 and can be easily distinguished by coloration, but morphological differences are subtle, e.g. variations of the male genitalia (Müller-Motzfeld 2006). Both species are also riparian specialists but are found in different regions. In Germany, specimens of *Bembidion atrocaeruleum* are documented in the low mountain ranges whereas beetles of *Bembidion varicolor* are inhabitants of the foothills of the Alps (Müller-Motzfeld 2006, Trautner et al. 2014). A similar situation is given in Switzerland (Luka et al. 2009). These two species are also found in other European countries, e.g. France, Italy, or Slovakia and Slovenia (see www.carabidae.org), but detailed distribution information are not available. However, such a close relationship of both species was not discussed before. Only detailed analysis of a) more specimens sampled from additional localities, b) other faster evolving nuclear markers, e.g. microsatellites or RAD-Seqs, c) ecological parameters, and d) comprehensive morphological and morphometric studies will give us more insights if we face two species or morphotypes of only one species.

### Species with high intraspecific variability

**Figure 4.**
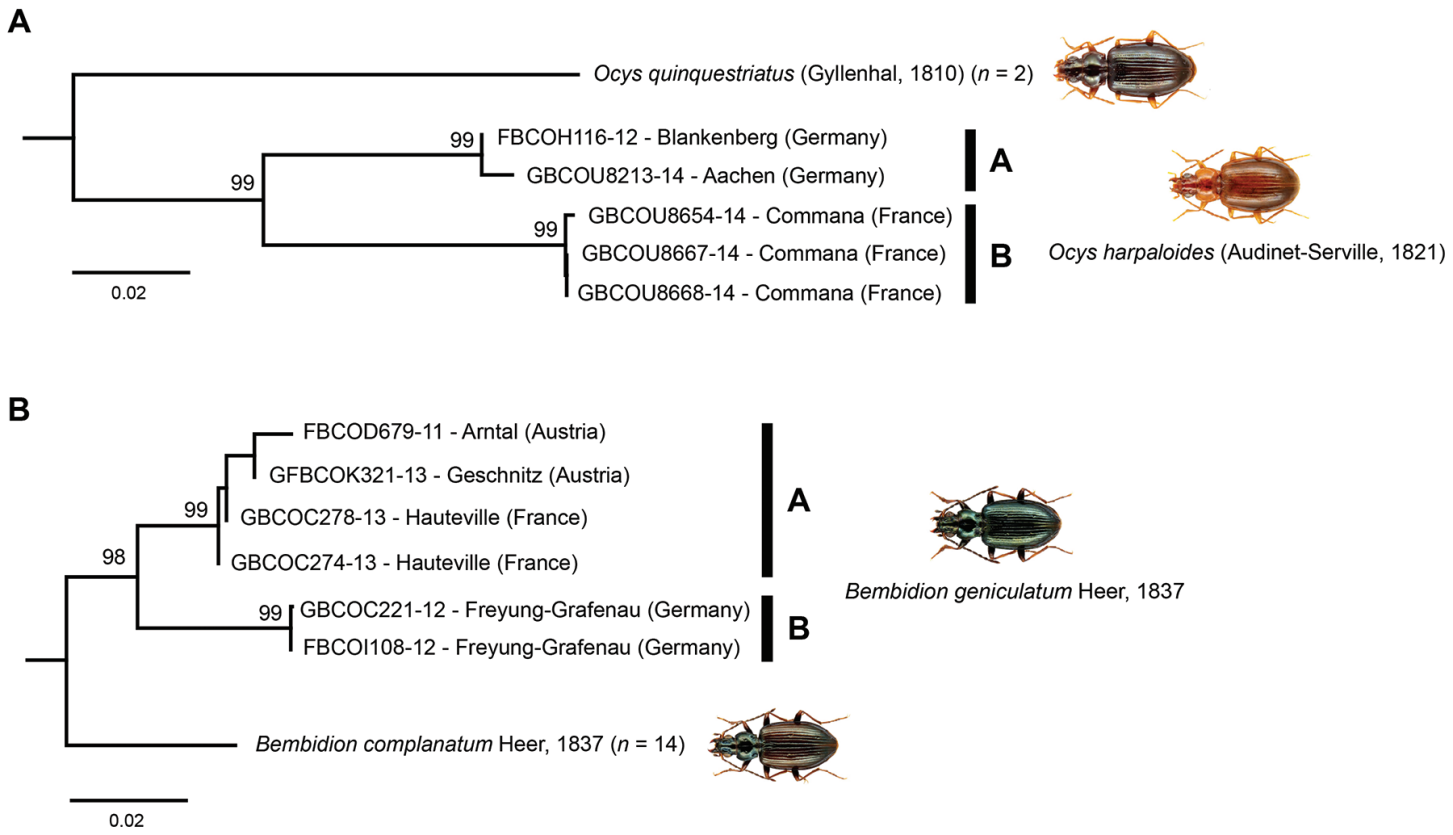
Subtrees of the neighbor joining topology based on Kimura 2-parameter distances of all analyzed specimens of **A** *Ocys harpaloides* (Audinet-Serville, 1821) and nearest neighbor, and **B** *Bembidion geniculatum* Heer, 1837 and nearest neighbor. Branches with specimen ID-number from BOLD, species names and sample localities. Numbers next to internal nodes are non-parametric bootstrap values (in %).

Maximum intraspecific pairwise distances >2.2% were observed for three species. Whereas *Bembidion decorum* showed no conspicuous substructure for the analyzed COI sequences (see Suppl. material 2), two distinct monophyletic lineages were revealed within *Bembidion geniculatum* as well as *Ocys harpaloides* (Fig. 4). In both cases, specimens sampled from Germany form monophyletic clusters (*Bembidion geniculatum*: A, *Ocys harpaloides*: B) that are separated from all other specimens (*Bembidion geniculatum*: four specimens from Austria and France, *Ocys harpaloides*: three specimens from France). Whereas distances between cluster A and B for *Bembidion geniculatum* range from 3.7 to 4.4%, distances from 8.8% to 9.6% are documented for *Ocys harpaloides*.

Based on the given data we are unable to clarify the reasons of the observed distinct lineages which can be caused by various effects, including phylogeographic events (e.g. Zhang et al. 2005, Zhang et al. 2006, Schmidt et al. 2012), infections of maternally inherited endosymbionts as *Wolbachia* (e.g. Roehrdanz and Levitan 2007, Werren et al. 2008, Gerth et al. 2011), or the presence of nuclear copies of mitochondrial DNA (numts) (Bensasson et al. 2001, Hazakani-Covo et al. 2010). Interestingly, numts have been shown for various *Bembidion* species (Maddison 2008, Maddison 2012, Maddison and Swanson 2010), but we found no evidence for any numts within our dataset. Finally, the observed variability may be also caused by the existence of overseen or cryptic species. It is obvious that more specimens of all four species have to be analyzed using morphological as well as molecular data to answer these questions in detail.

## Conclusions

Carabid beetles are one of the best-known taxa in entomology that have been studied intensively by numerous generations of coleopterists, clarifying their taxonomy and phylogeny, biogeography, habitat associations and ecological requirements, life history and adaptations, especially in Central Europe (see review in Kotze et al. 2011). Our analysis revealed some interesting results that should motivate carabidologists to check the species status of various “well known” species more in detail. Due to the fact that specimens of a number of species were collected at the same or close localities (e.g. *Bembidion octomaculatum* (Goeze, 1777)), or only a low number of specimens have been analyzed (e.g. *Bembidion striatum* (Fabricius, 1792)), the intraspecific variability of such species may be underestimated (e.g. Linares et al. 2009, Bergsten et al. 2012, but see Huemer et al. 2014). Nevertheless, our study clearly emphasizes the use of DNA barcodes for the identification of the analyzed ground beetles species of the genera *Asaphidion*, *Bembidion*, *Sinechostictus* and *Ocys*. Therefore, this data set represents an important step in building-up a comprehensive barcode library for the Carabidae in Germany which will be used in modern molecular biodiversity assessment studies.

## Supplementary materials

Supplementary material 1 Barcode analysis using the BOLD workbench Data type: Data table Explanation note: Molecular distances based on the Kimura 2-parameter model of the analyzed specimens of the analyzed species of the genera *Asaphidion*, *Bembidion*, *Ocys* and *Sinechostictus*. Divergence values were calculated for all studied sequences, using the Nearest Neighbour Summary implemented in the Barcode Gap Analysis tool provided by the Barcode of Life Data System (BOLD). Align sequencing option: BOLD aligner (amino acid based HMM), ambiguous base/gap handling: pairwise deletion. ISD = intraspecific distance. BINs are based on the barcode analysis from 01-02-2016. Asterisks indicate species not recorded from Germany. Species with intraspecific maximum pairwise distances >2.2% and species pairs with interspecific distances <2.2% are marked in bold. Country codes: A = Austria, B = Belgium, CZ = Czech Republic, D = Germany, F = France, I = Italy, S = Sweden and SLO = Slovenia. This dataset is made available under the Open Database License (http://opendatacommons.org/licenses/odbl/1.0/). The Open Database License (ODbL) is a license agreement intended to allow users to freely share, modify, and use this Dataset while maintaining this same freedom for others, provided that the original source and author(s) are credited.Michael J. Raupach, Karsten Hannig, Jérome Morinière, Lars Hendrich

Supplementary material 2 Neighbor joining topology Data type: Neighbor joining topology Explanation note: Neighbor joining topology of all analyzed carabid beetles based on Kimura 2-parameter distances. Specimens are classified using ID numbers from BOLD and species name. Numbers next to nodes represent non-parametric bootstrap values (1,000 replicates, in %). This dataset is made available under the Open Database License (http://opendatacommons.org/licenses/odbl/1.0/). The Open Database License (ODbL) is a license agreement intended to allow users to freely share, modify, and use this Dataset while maintaining this same freedom for others, provided that the original source and author(s) are credited.Michael J. Raupach, Karsten Hannig, Jérome Morinière, Lars Hendrich
