# Supplementary material for: A DNA barcode library for ground beetles (Insecta, Coleoptera, Carabidae) of Germany: The genus Bembidion Latreille, 1802 and allied taxa
Source: Zookeys. 2016 May 25;(592):121–41. doi: 10.3897/zookeys.592.8316 (PMC4926639; doi:10.3897/zookeys.592.8316)
Supplement: Supplementary material 1 — Barcode analysis using the BOLD workbench [file zookeys-592-121-s001.docx]

| No. | Species | Mean ISD | Max ISD | *n* | BIN | Country | Nearest Species | Distance to NN |
| --- | --- | --- | --- | --- | --- | --- | --- | --- |
| 1 | *Asaphidion austriacum* Schweiger, 1975 | 0 | 0 | 3 | AAW5817 | A, D | *Asaphidion flavipes* | 5.96 |
|  | *Asaphidion caraboides* (Schrank, 1781) | 0 | 0 | 3 | ABX8684 | A, I | *Asaphidion pallipes* | 12.71 |
|  | *Asaphidion curtum* (Heyden, 1870) | 0 | 0 | 5 | AAO0702 | B, D | *Asaphidion flavipes* | 7.83 |
|  | *Asaphidion flavipes* (Linnaeus, 1761) | 0.87 | 1.71 | 4 | ACC1923 | D | *Asaphidion austriacum* | 5.96 |
| 5 | *Asaphidion pallipes* (Duftschmid, 1812) | 0.15 | 0.15 | 2 | AAK0196 | D | *Bembidion dentellum* | 11.59 |
|  |  |  |  |  |  |  |  |  |
| 1 | *Bembidion argenteolum* Ahrens, 1812 | 0 | 0 | 6 | AAP9143 | D | *Bembidion striatum* | 9.64 |
|  | *Bembidion articulatum* (Panzer, 1796) | 0.08 | 0.62 | 21 | AAO3609 | D | *Bembidion normannum* | 11.32 |
|  | ***Bembidion ascendens* K. Daniel, 1902** | **0.06** | **0.15** | **5** | **ACJ7842** | **A, D** | ***Bembidion fasciolatum*** | **0.49** |
|  | *Bembidion aspericolle* (Germar, 1829) | 0.08 | 0.31 | 24 | AAY7788 | D | *Bembidion minimum* | 10.08 |
| 5 | *Bembidion assimile* Gyllenhal, 1810 | 0.67 | 1.41 | 10 | AAO0684 | D | *Bembidion fumigatum* | 6.4 |
|  | ***Bembidion atrocaeruleum* Stephens, 1828** | **0.08** | **0.65** | **32** | **AAO0687** | **D, F** | ***Bembidion varicolor*** | **0** |
|  | *Bembidion azurescens* Dalla Torre, 1877 | 0.1 | 0.32 | 8 | AAO3610 | A, F, D | *Bembidion tenellum* | 9.58 |
|  | *Bembidion biguttatum* (Fabricius, 1779) | 0.27 | 0.77 | 12 | AAW2883 | D | *Bembidion lunulatum* | 9.54 |
|  | *Bembidion bipunctatum* (Linnaeus, 1761) | 0.09 | 0.46 | 10 | ABA3256 | A, I | *Bembidion pallidipenne* | 9 |
| 10 | *Bembidion bruxellense* Wesmael, 1835 | 0.62 | 0.62 | 2 | AAW5839 | D | *Bembidion tetracolum* | 10.09 |
|  | *Bembidion complanatum* Heer, 1837 | 0.02 | 0.16 | 14 | ABA4132 | A, F, I | *Bembidion geniculatum* | 4.85 |
|  | *Bembidion conforme* (Dejean, 1831) | 0.14 | 0.46 | 6 | ABA4131 | A, I | *Bembidion atrocaeruleum* | 4.27 |
|  | *Bembidion cruciatum* (Dejean, 1831) | 0.55 | 1.68 | 10 | AAZ8054, ACJ4302 | A | *Bembidion tetracolum* | 7.19 |
|  | **Bembidion dalmatinum* Dejean, 1831 | 1.72 | 1.72 | 2 | ACD1082 | F, SLO | *Bembidion deletum* | 5.63 |
| 15 | ***Bembidion decorum* (Panzer, 1799)** | **0.38** | **2.56** | **26** | **AAO0686** | A, D, I | *Bembidion saxatile* | 8.27 |
|  | *Bembidion deletum* Audinet-Serville, 1821 | 0.24 | 0.62 | 8 | AAO0677 | A, D | *Bembidion dalmatinum* | 5.63 |
|  | *Bembidion dentellum* (Thunberg, 1787) | 0.39 | 2.03 | 16 | AAP8867 | D | *Bembidion quadrimaculatum* | 10.03 |
|  | *Bembidion doris* (Panzer, 1796) | 0 | 0 | 6 | AAN1997 | D | *Bembidion schueppelii* | 9.29 |
|  | *Bembidion eques* Sturm, 1825 | N/A | N/A | 1 | ACB9130 | A | *Bembidion fulvipes* | 8.3 |
| 20 | ***Bembidion fasciolatum* (Duftschmid, 1812)** | **0** | **0** | **4** | **ACJ7842** | **I** | ***Bembidion ascendens*** | **0.49** |
|  | *Bembidion femoratum* Sturm, 1825 | 0.28 | 0.93 | 18 | AAO4707 | D, F | *Bembidion cruciatum* | 7.77 |
|  | *Bembidion fluviatile* Dejean, 1832 | 0.18 | 0.48 | 17 | ACB8498 | D, F | *Bembidion incognitum* | 10.22 |
|  | *Bembidion fulvipes* Sturm, 1827 | 0 | 0 | 2 | ABA4133 | I | *Bembidion eques* | 8.3 |
|  | *Bembidion fumigatum* (Duftschmid, 1812) | 0.2 | 0.49 | 6 | ACB6903 | D | *Bembidion assimile* | 6.4 |
| 25 | *Bembidion genei illigeri* Netolitzky, 1914 | 0.4 | 1.88 | 16 | AAO3489 | D, F | *Bembidion glaciale* | 4.74 |
|  | ***Bembidion geniculatum* Heer, 1837** | **2.16** | **4.49** | **6** | **ACC4113, ACD1529** | **A, D, F** | ***Bembidion complanatum*** | **4.75** |
|  | *Bembidion gilvipes* Sturm, 1825 | 0.11 | 0.31 | 6 | AAW3429 | CZ, D | *Bembidion fumigatum* | 10.4 |
|  | *Bembidion glaciale* Heer, 1837 | 0.95 | 1.94 | 4 | ACJ4675 | A | *Bembidion genei illigeri* | 4.74 |
|  | ***Bembidion guttula* (Fabricius, 1792)** | **0.34** | **0.92** | **7** | **AAW3426** | **D** | ***Bembidion mannerheimii*** | **0** |
| 30 | *Bembidion incognitum* G. Müller, 1931 | 0.14 | 0.77 | 24 | AAP9536 | A, D | *Bembidion tetracolum* | 9.61 |
|  | *Bembidion iricolor* Bedel, 1879 | N/A | N/A | 1 | ABX8873 | D | *Bembidion lunulatum* | 3.98 |
|  | **Bembidion italicum* De Monte, 1943 | N/A | N/A | 1 | ACD1072 | SLO | *Bembidion milleri* | 8.1 |
|  | *Bembidion lampros* (Herbst, 1784) | 0.08 | 0.79 | 20 | AAO0675 | A, D, F | *Bembidion properans* | 8.06 |
|  | *Bembidion litorale* (Olivier, 1790) | 0.38 | 1.08 | 16 | AAP9487 | D | *Bembidion velox* | 8.62 |
| 35 | *Bembidion lunulatum* (Geoffroy, 1785) | 0.27 | 1.55 | 10 | AAP9142 | B, D, SLO | *Bembidion iricolor* | 3.98 |
|  | ***Bembidion mannerheimii* C.R. Sahlberg, 1827** | **0.33** | **0.77** | **14** | **AAW3426** | **B, D** | ***Bembidion guttula*** | **0** |
|  | *Bembidion milleri* Jacquelin du Val, 1852 | 0.17 | 0.92 | 11 | AAP9488 | D | *Bembidion deletum* | 6.56 |
|  | *Bembidion minimum* (Fabricius, 1792) | 0 | 0 | 8 | ABW4318 | D | *Bembidion quadrimaculatum* | 8.37 |
|  | *Bembidion modestum* (Fabricius, 1801) | 0.31 | 0.77 | 16 | AAO4709 | D | *Bembidion saxatile* | 7.28 |
| 40 | *Bembidion monticola* Sturm, 1825 | 0.26 | 1.01 | 13 | ABA7653 | D, I | *Bembidion atrocaeruleum* | 6.05 |
|  | *Bembidion nigricorne* Gyllenhal, 1827 | N/A | N/A | 1 | AAQ1100 | D | *Bembidion genei illigeri* | 10.83 |
|  | *Bembidion normannum* Dejean, 1831 | 0.04 | 0.32 | 14 | AAP9489 | D, F | *Bembidion minimum* | 8.42 |
|  | *Bembidion obliquum* Sturm, 1825 | N/A | N/A | 1 | ABW9045 | D | *Bembidion semipunctatum* | 10.41 |
|  | *Bembidion obtusum* Audinet-Serville, 1821 | 0.57 | 1.72 | 15 | AAP9490 | D | *Bembidion glaciale* | 9.73 |
| 45 | *Bembidion octomaculatum* (Goeze, 1777) | 0.39 | 1.09 | 7 | AAW3499 | D | *Bembidion minimum* | 9.44 |
|  | *Bembidion pallidipenne* (Illiger, 1802) | 0.07 | 0.15 | 8 | AAP9685 | D | *Bembidion tibiale* | 8.02 |
|  | *Bembidion prasinum* (Duftschmid, 1812) | 0 | 0 | 3 | AAO3481 | D | *Bembidion genei illigeri* | 9.8 |
|  | *Bembidion properans* (Stephens, 1828) | 0.59 | 1.87 | 24 | AAO3482 | D | *Bembidion lampros* | 8.06 |
|  | *Bembidion punctulatum* Drapiez, 1820 | 0.34 | 0.68 | 19 | AAO4703 | A, D | *Bembidion ascendens* | 10.31 |
| 50 | *Bembidion pygmaeum* (Fabricius, 1792) | 0.13 | 0.31 | 9 | AAO3483 | D | *Asaphidion flavipes* | 11.08 |
|  | *Bembidion quadrimaculatum* (Linnaeus, 1761) | 0.22 | 1.01 | 28 | AAF0278 | A, D | *Bembidion quadripustulatum* | 6.56 |
|  | *Bembidion quadripustulatum* Audinet-Serville, 1821 | N/A | N/A | 1 | AAP8287 | D | *Bembidion quadrimaculatum* | 6.56 |
|  | *Bembidion ruficolle* (Panzer, 1796) | 0.52 | 0.77 | 3 | ACB8438 | D | *Bembidion glaciale* | 9.28 |
|  | *Bembidion saxatile* Gyllenhal, 1827 | N/A | N/A | 1 | ABW9273 | S | *Bembidion modestum* | 7.28 |
| 55 | *Bembidion schueppelii* Dejean, 1831 | 0.21 | 0.32 | 3 | ABX2366 | A, D | *Bembidion assimile* | 7.41 |
|  | *Bembidion semipunctatum* (Donovan, 1806) | 0.29 | 0.93 | 12 | AAO0679 | D | *Bembidion varium* | 6.58 |
|  | *Bembidion stephensii* Crotch, 1869 | N/A | N/A | 1 | AAY7941 | D | *Bembidion deletum* | 12.73 |
|  | *Bembidion striatum* (Fabricius, 1792) | 0.31 | 0.46 | 3 | AAP8865 | D | *Bembidion litorale* | 9.58 |
|  | *Bembidion tenellum* Erichson, 1837 | 0.49 | 1.3 | 17 | AAY7797 | D, SLO | *Bembidion minimum* | 9.28 |
| 60 | *Bembidion testaceum* (Duftschmid, 1812) | 0.62 | 1.4 | 10 | AAO0654 | A, D, F | *Bembidion cruciatum* | 10.24 |
|  | *Bembidion tetracolum* Say, 1823 | 0.3 | 0.93 | 38 | AAH0101 | A, D, F | *Bembidion cruciatum* | 7.19 |
|  | *Bembidion tibiale* (Duftschmid, 1812) | 0.43 | 1.89 | 25 | AAQ1703 | A, D, I, SLO | *Bembidion fasciolatum* | 3.31 |
|  | ***Bembidion varicolor* Fabricius, 1803** | **0.1** | **0.8** | **22** | **AAO0687** | **A, D, I, SLO** | ***Bembidion atrocaeruleum*** | **0** |
|  | *Bembidion varium* (Olivier, 1795) | 0.5 | 2.19 | 29 | AAJ8206 | D, F | *Bembidion semipunctatum* | 6.58 |
| 65 | *Bembidion velox* (Linnaeus, 1761) | 0.71 | 1.61 | 13 | AAP8868 | D | *Bembidion litorale* | 8.62 |
|  |  |  |  |  |  |  |  |  |
| 1 | ***Ocys harpaloides* (Audinet-Serville, 1821)** | **5.61** | **9.62** | **5** | **ACB9625, ACR8306** | **D, F** | *Bembidion articulatum* | 12.8 |
|  | *Ocys quinquestriatus* (Gyllenhal, 1810) | 0.15 | 0.15 | 2 | ACR4156 | D | *Bembidion fumigatum* | 14.72 |
|  |  |  |  |  |  |  |  |  |
| 1 | *Sinechostictus decoratus* (Duftschmid, 1812) | 0.22 | 0.46 | 7 | AAP9537 | A, I | *Sinechostictus elongatus* | 7.92 |
|  | *Sinechostictus elongatus* (Dejean, 1831) | 0.54 | 1.39 | 8 | AAI8642 | D | *Sinechostictus decoratus* | 7.92 |
|  | *Sinechostictus inustus* (Jacquelin du Val, 1857) | 0.15 | 0.15 | 2 | ACR8780 | D | *Sinechostictus millerianus* | 10.16 |
|  | *Sinechostictus millerianus* (Heyden, 1883) | 0.14 | 0.31 | 11 | AAP9683 | D | *Sinechostictus ruficornis* | 4.28 |
| 5 | *Sinechostictus ruficornis* (Sturm, 1825) | 0.05 | 0.33 | 12 | AAQ1955 | A, D, I | *Sinechostictus millerianus* | 4.28 |
|  | *Sinechostictus stomoides* (Dejean, 1831) | 0.2 | 0.46 | 9 | ABA7648 | A, D, I | *Sinechostictus ruficornis* | 5.04 |
