# Supplementary material for: A DNA barcode library for ground beetles (Insecta, Coleoptera, Carabidae) of Germany: The genus Bembidion Latreille, 1802 and allied taxa
Source: Zookeys. 2016 May 25;(592):121–41. doi: 10.3897/zookeys.592.8316 (PMC4926639; doi:10.3897/zookeys.592.8316)
Supplement: Supplementary material 2 — Neighbor joining topology [file zookeys-592-121-s002.pdf]

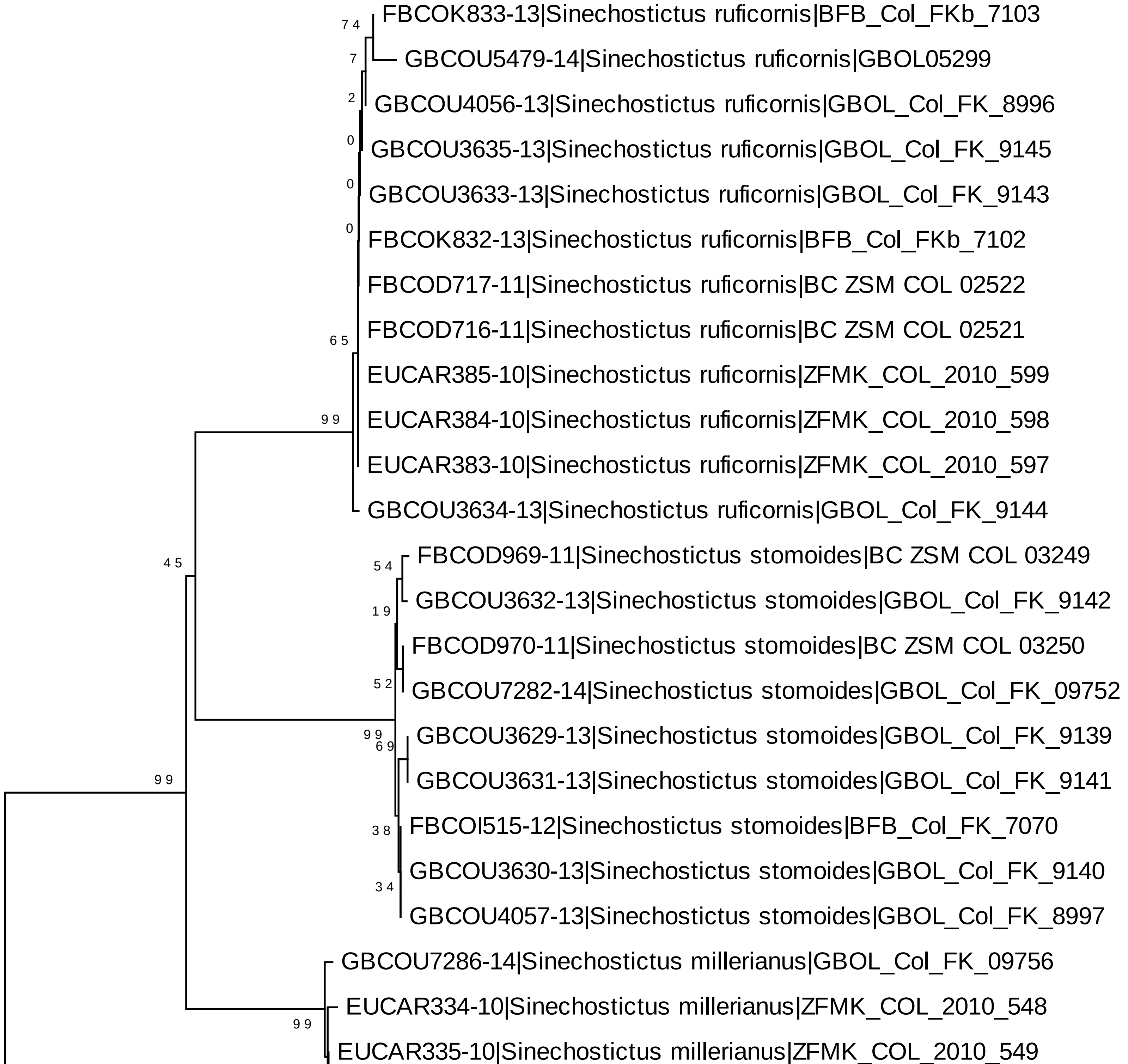

FBCOK833-13|Sinechostictus ruficornis|BFB_Col_FKb_7103
74
GBCOU5479-14|Sinechostictus ruficornis|GBOL05299
7
GBCOU4056-13|Sinechostictus ruficornis|GBOL_Col_FK_8996
2
GBCOU3635-13|Sinechostictus ruficornis|GBOL_Col_FK_9145
0
GBCOU3633-13|Sinechostictus ruficornis|GBOL_Col_FK_9143
0
FBCOK832-13|Sinechostictus ruficornis|BFB_Col_FKb_7102
0
FBCOD717-11|Sinechostictus ruficornis|BC ZSM COL 02522
FBCOD716-11|Sinechostictus ruficornis|BC ZSM COL 02521
65
EUCAR385-10|Sinechostictus ruficornis|ZFMK_COL_2010_599
99
EUCAR384-10|Sinechostictus ruficornis|ZFMK_COL_2010_598
EUCAR383-10|Sinechostictus ruficornis|ZFMK_COL_2010_597
GBCOU3634-13|Sinechostictus ruficornis|GBOL_Col_FK_9144
45
FBCOD969-11|Sinechostictus stomoides|BC ZSM COL 03249
54
GBCOU3632-13|Sinechostictus stomoides|GBOL_Col_FK_9142
19
FBCOD970-11|Sinechostictus stomoides|BC ZSM COL 03250
52
GBCOU7282-14|Sinechostictus stomoides|GBOL_Col_FK_09752
99
69
GBCOU3629-13|Sinechostictus stomoides|GBOL_Col_FK_9139
GBCOU3631-13|Sinechostictus stomoides|GBOL_Col_FK_9141
99
38
FBCOI515-12|Sinechostictus stomoides|BFB_Col_FK_7070
GBCOU3630-13|Sinechostictus stomoides|GBOL_Col_FK_9140
34
GBCOU4057-13|Sinechostictus stomoides|GBOL_Col_FK_8997
GBCOU7286-14|Sinechostictus millerianus|GBOL_Col_FK_09756
EUCAR334-10|Sinechostictus millerianus|ZFMK_COL_2010_548
99
EUCAR335-10|Sinechostictus millerianus|ZFMK_COL_2010_549

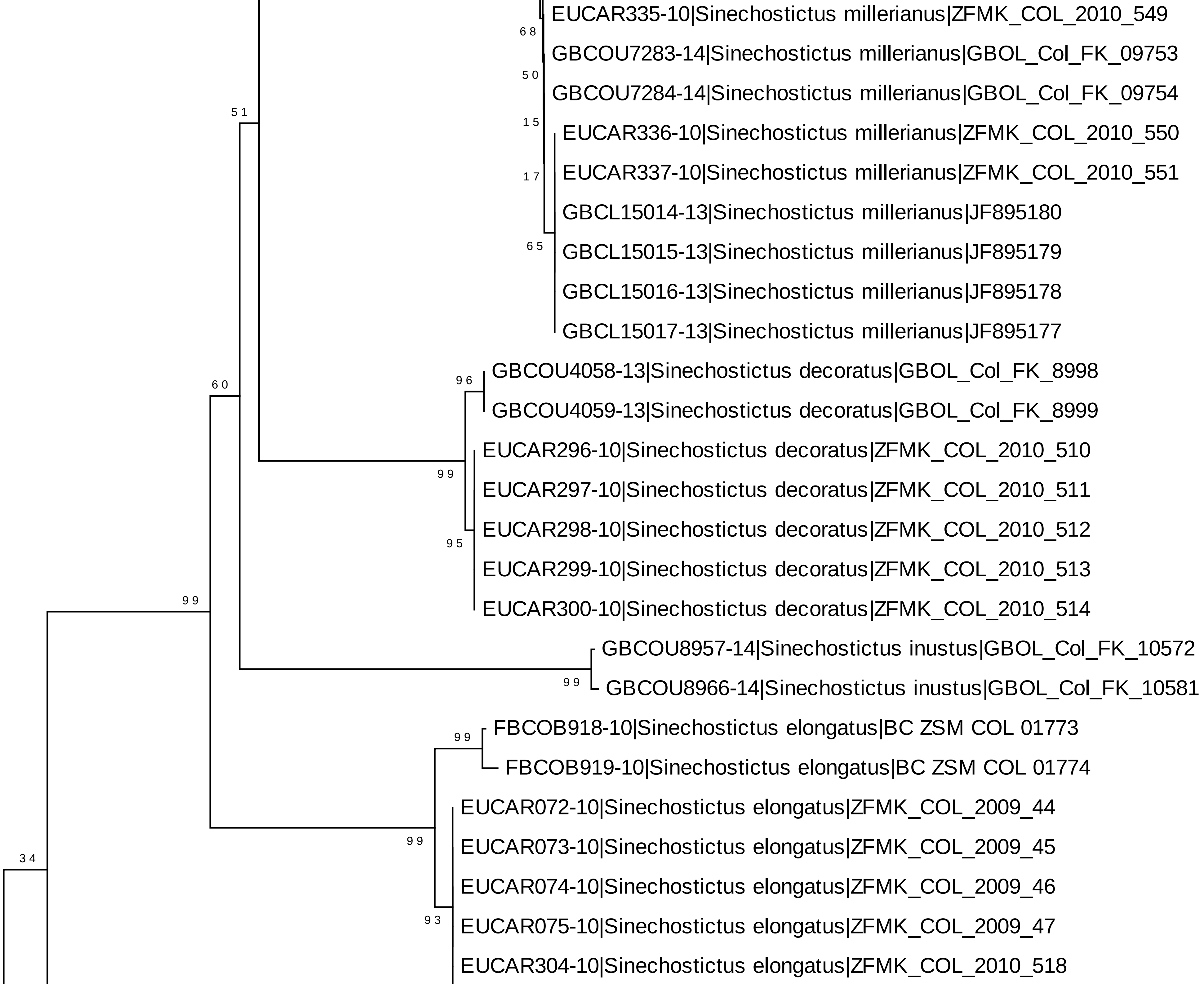
EUCAR335-10|Sinechostictus millerianus|ZFMK_COL_2010_549
68
GBCOU7283-14|Sinechostictus millerianus|GBOL_Col_FK_09753
50
GBCOU7284-14|Sinechostictus millerianus|GBOL_Col_FK_09754
51
15
EUCAR336-10|Sinechostictus millerianus|ZFMK_COL_2010_550
17
EUCAR337-10|Sinechostictus millerianus|ZFMK_COL_2010_551
GBCL15014-13|Sinechostictus millerianus|JF895180
65
GBCL15015-13|Sinechostictus millerianus|JF895179
GBCL15016-13|Sinechostictus millerianus|JF895178
GBCL15017-13|Sinechostictus millerianus|JF895177
96
GBCOU4058-13|Sinechostictus decoratus|GBOL_Col_FK_8998
60
GBCOU4059-13|Sinechostictus decoratus|GBOL_Col_FK_8999
EUCAR296-10|Sinechostictus decoratus|ZFMK_COL_2010_510
99
EUCAR297-10|Sinechostictus decoratus|ZFMK_COL_2010_511
EUCAR298-10|Sinechostictus decoratus|ZFMK_COL_2010_512
95
EUCAR299-10|Sinechostictus decoratus|ZFMK_COL_2010_513
99
EUCAR300-10|Sinechostictus decoratus|ZFMK_COL_2010_514
GBCOU8957-14|Sinechostictus inustus|GBOL_Col_FK_10572
99
GBCOU8966-14|Sinechostictus inustus|GBOL_Col_FK_10581
99
FBCOB918-10|Sinechostictus elongatus|BC ZSM COL 01773
FBCOB919-10|Sinechostictus elongatus|BC ZSM COL 01774
EUCAR072-10|Sinechostictus elongatus|ZFMK_COL_2009_44
99
34
EUCAR073-10|Sinechostictus elongatus|ZFMK_COL_2009_45
EUCAR074-10|Sinechostictus elongatus|ZFMK_COL_2009_46
93
EUCAR075-10|Sinechostictus elongatus|ZFMK_COL_2009_47
EUCAR304-10|Sinechostictus elongatus|ZFMK_COL_2010_518

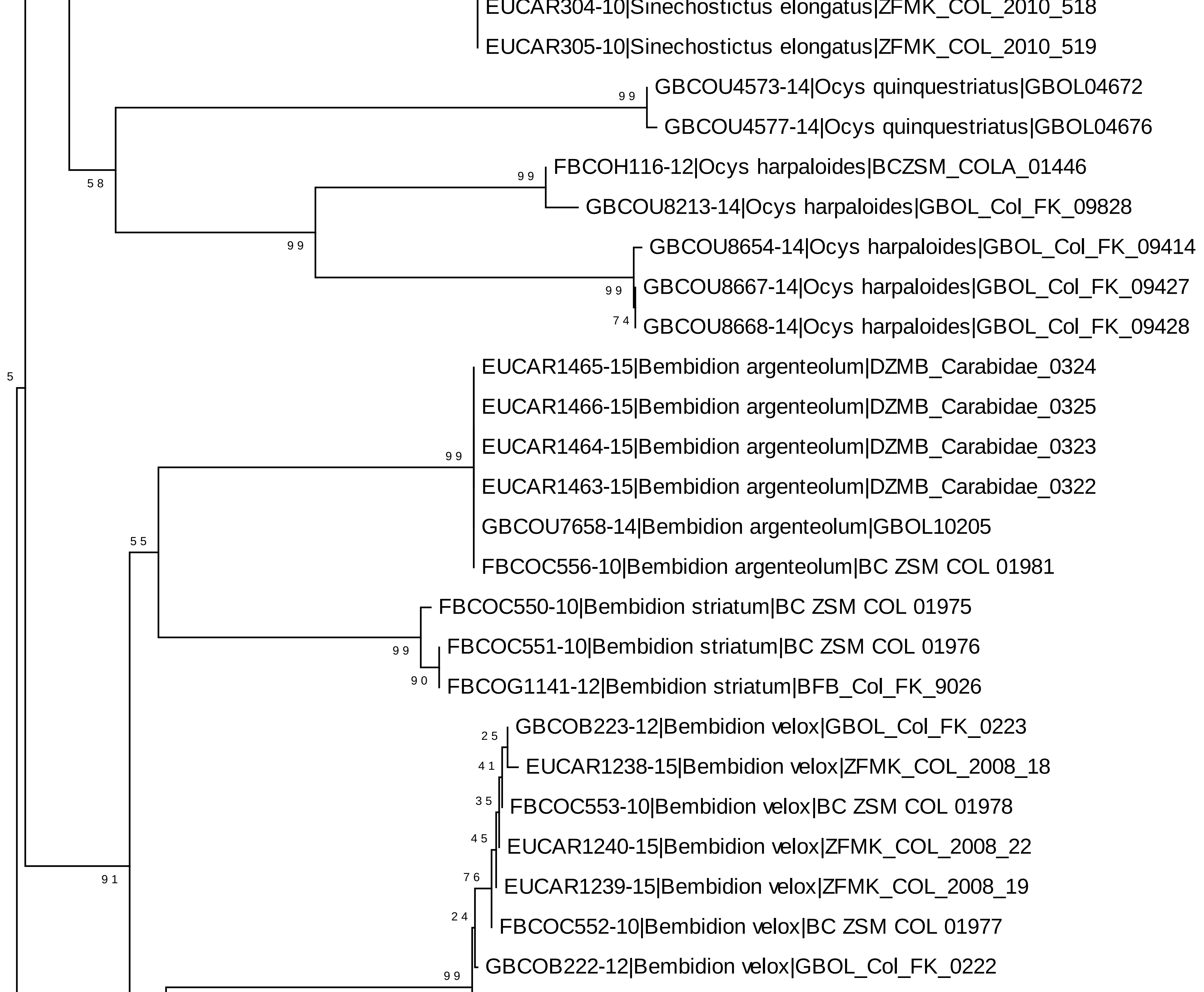
EUCAR304-10|Sinechostictus elongatus|ZFMK_COL_2010_518
EUCAR305-10|Sinechostictus elongatus|ZFMK_COL_2010_519
99
GBCOU4573-14|Ocys quinquestriatus|GBOL04672
GBCOU4577-14|Ocys quinquestriatus|GBOL04676
99
FBCOH116-12|Ocys harpaloides|BCZSM_COLA_01446
58
GBCOU8213-14|Ocys harpaloides|GBOL_Col_FK_09828
99
GBCOU8654-14|Ocys harpaloides|GBOL_Col_FK_09414
99
GBCOU8667-14|Ocys harpaloides|GBOL_Col_FK_09427
74
GBCOU8668-14|Ocys harpaloides|GBOL_Col_FK_09428
5
EUCAR1465-15|Bembidion argenteolum|DZMB_Carabidae_0324
EUCAR1466-15|Bembidion argenteolum|DZMB_Carabidae_0325
99
EUCAR1464-15|Bembidion argenteolum|DZMB_Carabidae_0323
EUCAR1463-15|Bembidion argenteolum|DZMB_Carabidae_0322
GBCOU7658-14|Bembidion argenteolum|GBOL10205
55
FBCOC556-10|Bembidion argenteolum|BC ZSM COL 01981
FBCOC550-10|Bembidion striatum|BC ZSM COL 01975
99
FBCOC551-10|Bembidion striatum|BC ZSM COL 01976
90
FBCOG1141-12|Bembidion striatum|BFB_Col_FK_9026
25
GBCOB223-12|Bembidion velox|GBOL_Col_FK_0223
41
EUCAR1238-15|Bembidion velox|ZFMK_COL_2008_18
35
FBCOC553-10|Bembidion velox|BC ZSM COL 01978
45
EUCAR1240-15|Bembidion velox|ZFMK_COL_2008_22
76
91
EUCAR1239-15|Bembidion velox|ZFMK_COL_2008_19
24
FBCOC552-10|Bembidion velox|BC ZSM COL 01977
99
GBCOB222-12|Bembidion velox|GBOL_Col_FK_0222

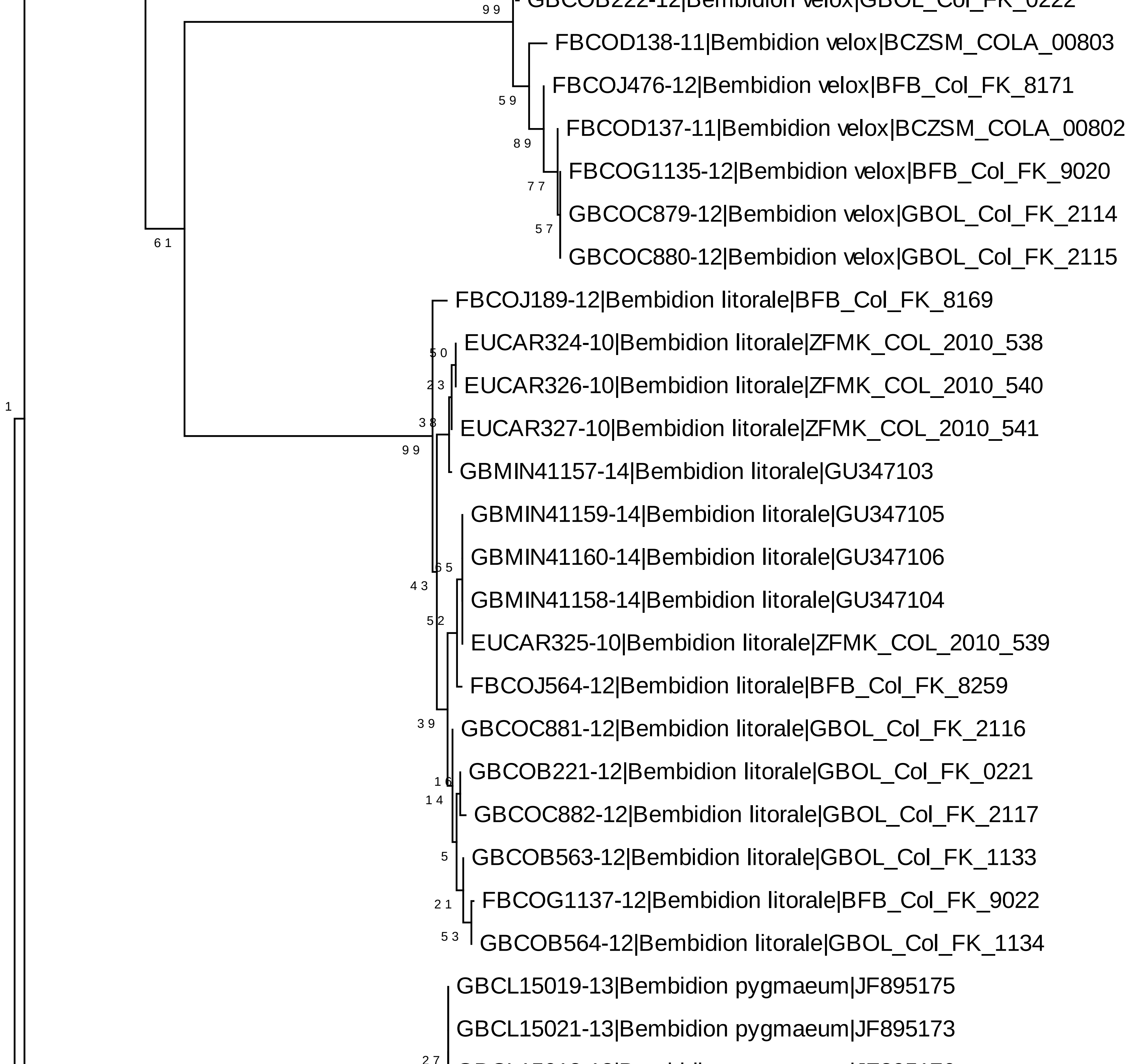
FBCOD138-11|Bembidion velox|BCZSM_COLA_00803
FBCOJ476-12|Bembidion velox|BFB_Col_FK_8171
FBCOD137-11|Bembidion velox|BCZSM_COLA_00802
FBCOG1135-12|Bembidion velox|BFB_Col_FK_9020
GBCOC879-12|Bembidion velox|GBOL_Col_FK_2114
GBCOC880-12|Bembidion velox|GBOL_Col_FK_2115
FBCOJ189-12|Bembidion litorale|BFB_Col_FK_8169
EUCAR324-10|Bembidion litorale|ZFMK_COL_2010_538
EUCAR326-10|Bembidion litorale|ZFMK_COL_2010_540
EUCAR327-10|Bembidion litorale|ZFMK_COL_2010_541
GBMIN41157-14|Bembidion litorale|GU347103
GBMIN41159-14|Bembidion litorale|GU347105
GBMIN41160-14|Bembidion litorale|GU347106
GBMIN41158-14|Bembidion litorale|GU347104
EUCAR325-10|Bembidion litorale|ZFMK_COL_2010_539
FBCOJ564-12|Bembidion litorale|BFB_Col_FK_8259
GBCOC881-12|Bembidion litorale|GBOL_Col_FK_2116
GBCOB221-12|Bembidion litorale|GBOL_Col_FK_0221
GBCOC882-12|Bembidion litorale|GBOL_Col_FK_2117
GBCOB563-12|Bembidion litorale|GBOL_Col_FK_1133
FBCOG1137-12|Bembidion litorale|BFB_Col_FK_9022
GBCOB564-12|Bembidion litorale|GBOL_Col_FK_1134
GBCL15019-13|Bembidion pygmaeum|JF895175
GBCL15021-13|Bembidion pygmaeum|JF895173

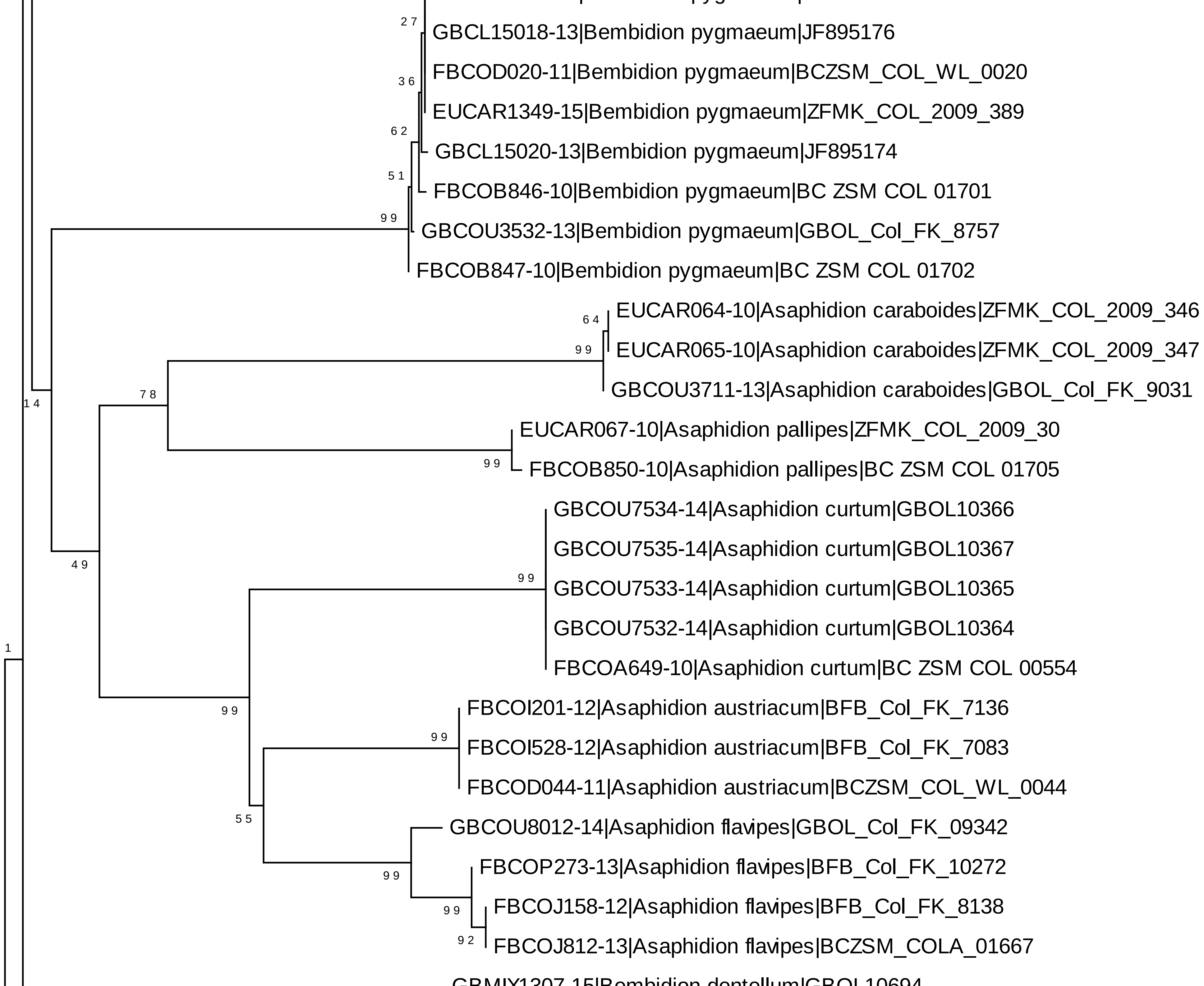
GBCL15018-13|Bembidion pygmaeum|JF895176
FBCOD020-11|Bembidion pygmaeum|BCZSM_COL_WL_0020
EUCAR1349-15|Bembidion pygmaeum|ZFMK_COL_2009_389
GBCL15020-13|Bembidion pygmaeum|JF895174
FBCOB846-10|Bembidion pygmaeum|BC ZSM COL 01701
GBCOU3532-13|Bembidion pygmaeum|GBOL_Col_FK_8757
FBCOB847-10|Bembidion pygmaeum|BC ZSM COL 01702
EUCAR064-10|Asaphidion caraboides|ZFMK_COL_2009_346
EUCAR065-10|Asaphidion caraboides|ZFMK_COL_2009_347
GBCOU3711-13|Asaphidion caraboides|GBOL_Col_FK_9031
EUCAR067-10|Asaphidion pallipes|ZFMK_COL_2009_30
FBCOB850-10|Asaphidion pallipes|BC ZSM COL 01705
GBCOU7534-14|Asaphidion curtum|GBOL10366
GBCOU7535-14|Asaphidion curtum|GBOL10367
GBCOU7533-14|Asaphidion curtum|GBOL10365
GBCOU7532-14|Asaphidion curtum|GBOL10364
FBCOA649-10|Asaphidion curtum|BC ZSM COL 00554
FBCOI201-12|Asaphidion austriacum|BFB_Col_FK_7136
FBCOI528-12|Asaphidion austriacum|BFB_Col_FK_7083
FBCOD044-11|Asaphidion austriacum|BCZSM_COL_WL_0044
GBCOU8012-14|Asaphidion flavipes|GBOL_Col_FK_09342
FBCOP273-13|Asaphidion flavipes|BFB_Col_FK_10272
FBCOJ158-12|Asaphidion flavipes|BFB_Col_FK_8138
FBCOJ812-13|Asaphidion flavipes|BCZSM_COLA_01667

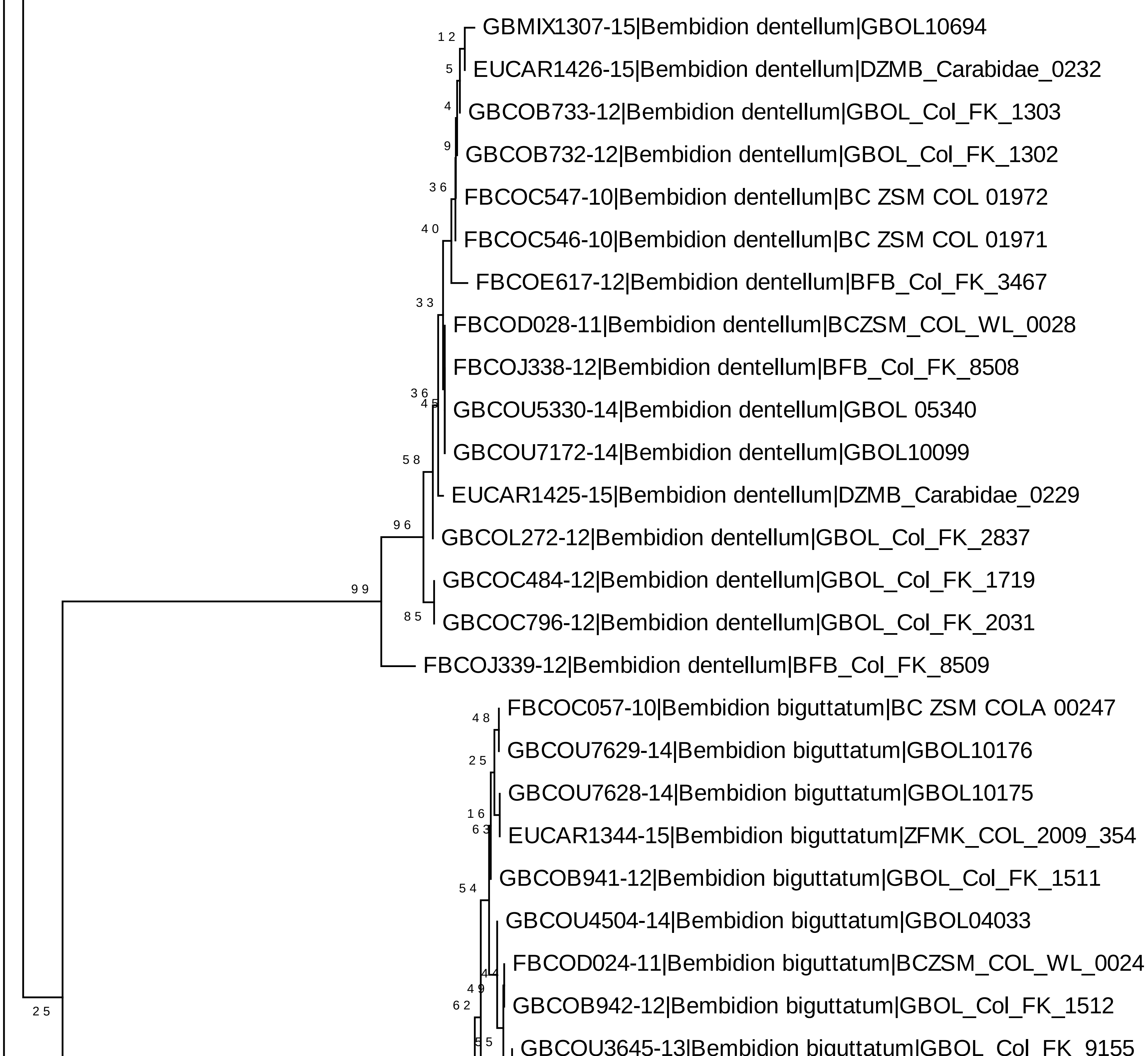
GBMIX1307-15|Bembidion dentellum|GBOL10694
EUCAR1426-15|Bembidion dentellum|DZMB_Carabidae_0232
GBCOB733-12|Bembidion dentellum|GBOL_Col_FK_1303
GBCOB732-12|Bembidion dentellum|GBOL_Col_FK_1302
FBCOC547-10|Bembidion dentellum|BC ZSM COL 01972
FBCOC546-10|Bembidion dentellum|BC ZSM COL 01971
FBCOE617-12|Bembidion dentellum|BFB_Col_FK_3467
FBCOD028-11|Bembidion dentellum|BCZSM_COL_WL_0028
FBCOJ338-12|Bembidion dentellum|BFB_Col_FK_8508
GBCOU5330-14|Bembidion dentellum|GBOL 05340
GBCOU7172-14|Bembidion dentellum|GBOL10099
EUCAR1425-15|Bembidion dentellum|DZMB_Carabidae_0229
GBCOL272-12|Bembidion dentellum|GBOL_Col_FK_2837
GBCOC484-12|Bembidion dentellum|GBOL_Col_FK_1719
GBCOC796-12|Bembidion dentellum|GBOL_Col_FK_2031
FBCOJ339-12|Bembidion dentellum|BFB_Col_FK_8509
FBCOC057-10|Bembidion biguttatum|BC ZSM COLA 00247
GBCOU7629-14|Bembidion biguttatum|GBOL10176
GBCOU7628-14|Bembidion biguttatum|GBOL10175
EUCAR1344-15|Bembidion biguttatum|ZFMK_COL_2009_354
GBCOB941-12|Bembidion biguttatum|GBOL_Col_FK_1511
GBCOU4504-14|Bembidion biguttatum|GBOL04033
FBCOD024-11|Bembidion biguttatum|BCZSM_COL_WL_0024
GBCOB942-12|Bembidion biguttatum|GBOL_Col_FK_1512
GBCOU3645-13|Bembidion biguttatum|GBOL_Col_FK_9155
12
5
4
9
36
40
33
36
45
58
96
99
85
48
25
16
63
54
49
62
55
25

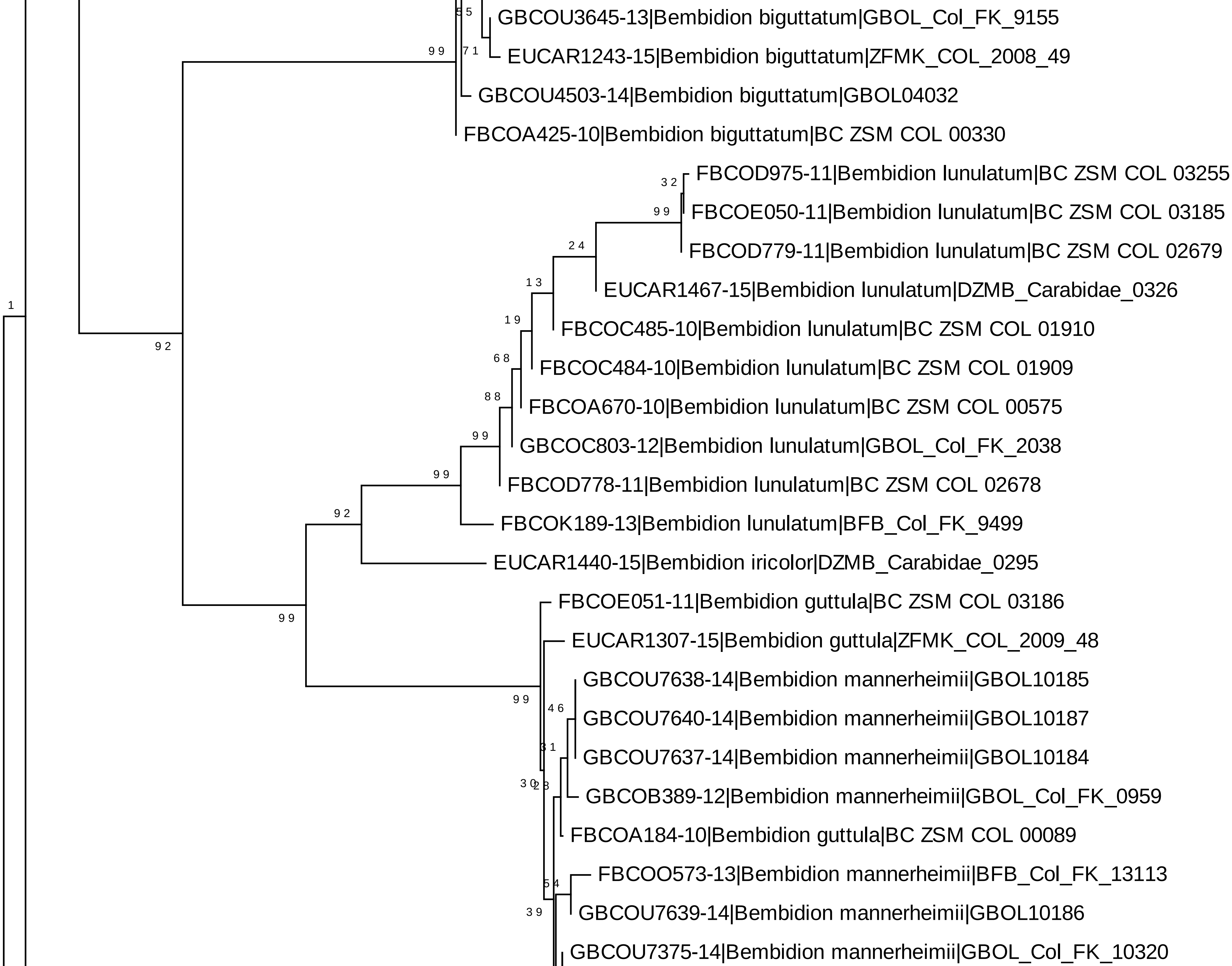
GBCOU3645-13|Bembidion biguttatum|GBOL_Col_FK_9155
EUCAR1243-15|Bembidion biguttatum|ZFMK_COL_2008_49
GBCOU4503-14|Bembidion biguttatum|GBOL04032
FBCOA425-10|Bembidion biguttatum|BC ZSM COL 00330
FBCOD975-11|Bembidion lunulatum|BC ZSM COL 03255
FBCOE050-11|Bembidion lunulatum|BC ZSM COL 03185
FBCOD779-11|Bembidion lunulatum|BC ZSM COL 02679
EUCAR1467-15|Bembidion lunulatum|DZMB_Carabidae_0326
FBCOC485-10|Bembidion lunulatum|BC ZSM COL 01910
FBCOC484-10|Bembidion lunulatum|BC ZSM COL 01909
FBCOA670-10|Bembidion lunulatum|BC ZSM COL 00575
GBCOC803-12|Bembidion lunulatum|GBOL_Col_FK_2038
FBCOD778-11|Bembidion lunulatum|BC ZSM COL 02678
FBCOK189-13|Bembidion lunulatum|BFB_Col_FK_9499
EUCAR1440-15|Bembidion iricolor|DZMB_Carabidae_0295
FBCOE051-11|Bembidion guttula|BC ZSM COL 03186
EUCAR1307-15|Bembidion guttula|ZFMK_COL_2009_48
GBCOU7638-14|Bembidion mannerheimii|GBOL10185
GBCOU7640-14|Bembidion mannerheimii|GBOL10187
GBCOU7637-14|Bembidion mannerheimii|GBOL10184
GBCOB389-12|Bembidion mannerheimii|GBOL_Col_FK_0959
FBCOA184-10|Bembidion guttula|BC ZSM COL 00089
FBCOO573-13|Bembidion mannerheimii|BFB_Col_FK_13113
GBCOU7639-14|Bembidion mannerheimii|GBOL10186
GBCOU7375-14|Bembidion mannerheimii|GBOL_Col_FK_10320
55
71
99
32
99
24
13
19
1
92
68
88
99
99
92
99
99
46
31
30
28
54
39

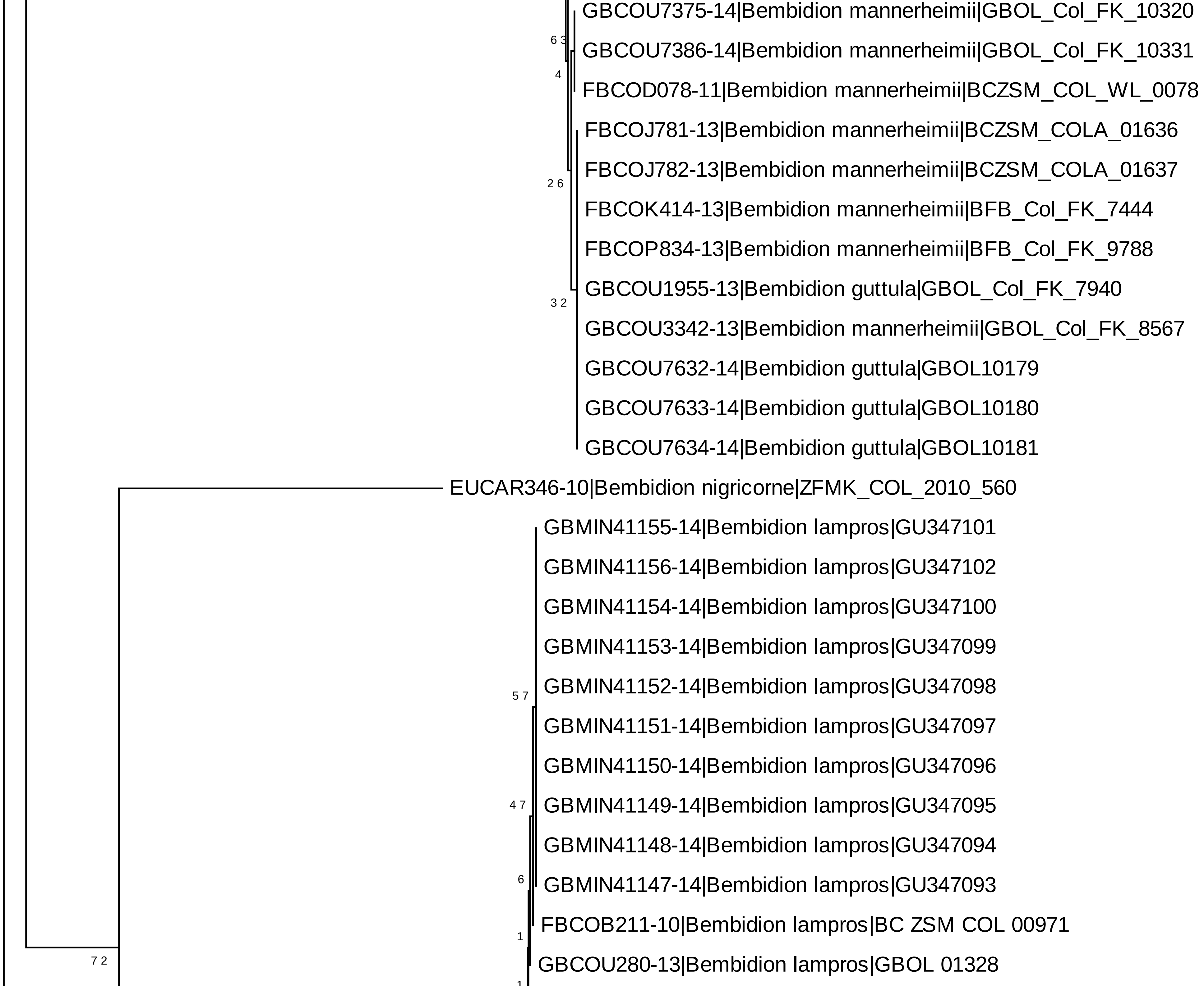
GBCOU7375-14|Bembidion mannerheimii|GBOL_Col_FK_10320
6 3
GBCOU7386-14|Bembidion mannerheimii|GBOL_Col_FK_10331
4
FBCOD078-11|Bembidion mannerheimii|BCZSM_COL_WL_0078
FBCOJ781-13|Bembidion mannerheimii|BCZSM_COLA_01636
FBCOJ782-13|Bembidion mannerheimii|BCZSM_COLA_01637
2 6
FBCOK414-13|Bembidion mannerheimii|BFB_Col_FK_7444
FBCOP834-13|Bembidion mannerheimii|BFB_Col_FK_9788
GBCOU1955-13|Bembidion guttula|GBOL_Col_FK_7940
3 2
GBCOU3342-13|Bembidion mannerheimii|GBOL_Col_FK_8567
GBCOU7632-14|Bembidion guttula|GBOL10179
GBCOU7633-14|Bembidion guttula|GBOL10180
GBCOU7634-14|Bembidion guttula|GBOL10181
EUCAR346-10|Bembidion nigricorne|ZFMK_COL_2010_560
GBMIN41155-14|Bembidion lampros|GU347101
GBMIN41156-14|Bembidion lampros|GU347102
GBMIN41154-14|Bembidion lampros|GU347100
GBMIN41153-14|Bembidion lampros|GU347099
GBMIN41152-14|Bembidion lampros|GU347098
5 7
GBMIN41151-14|Bembidion lampros|GU347097
GBMIN41150-14|Bembidion lampros|GU347096
4 7
GBMIN41149-14|Bembidion lampros|GU347095
GBMIN41148-14|Bembidion lampros|GU347094
6
GBMIN41147-14|Bembidion lampros|GU347093
FBCOB211-10|Bembidion lampros|BC ZSM COL 00971
1
GBCOU280-13|Bembidion lampros|GBOL 01328
7 2

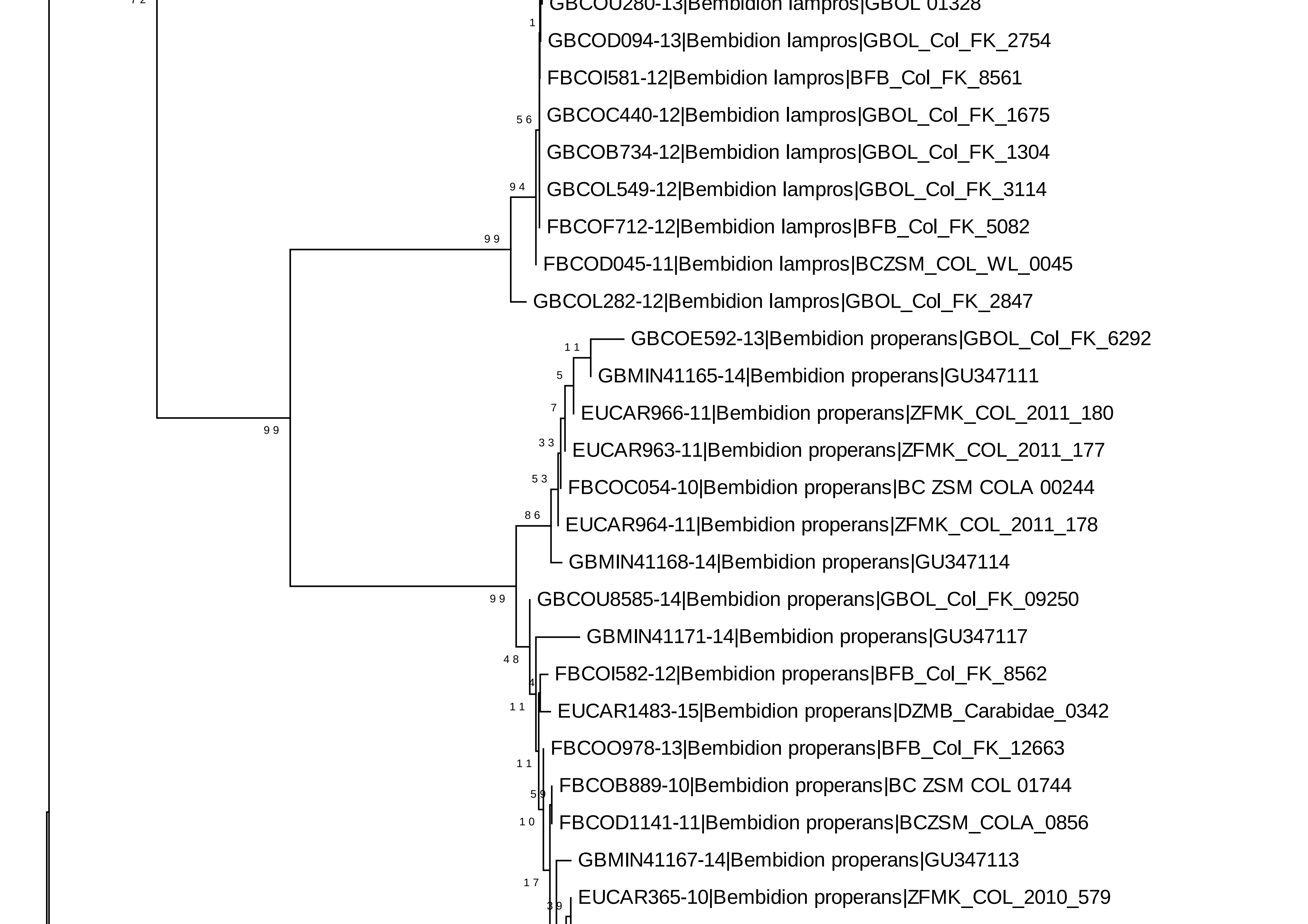
GBCOU280-13|Bembidion lampros|GBOL 01328
GBCOD094-13|Bembidion lampros|GBOL_Col_FK_2754
FBCOI581-12|Bembidion lampros|BFB_Col_FK_8561
GBCOC440-12|Bembidion lampros|GBOL_Col_FK_1675
GBCOB734-12|Bembidion lampros|GBOL_Col_FK_1304
GBCOL549-12|Bembidion lampros|GBOL_Col_FK_3114
FBCOF712-12|Bembidion lampros|BFB_Col_FK_5082
FBCOD045-11|Bembidion lampros|BCZSM_COL_WL_0045
GBCOL282-12|Bembidion lampros|GBOL_Col_FK_2847
GBCOE592-13|Bembidion properans|GBOL_Col_FK_6292
GBMIN41165-14|Bembidion properans|GU347111
EUCAR966-11|Bembidion properans|ZFMK_COL_2011_180
EUCAR963-11|Bembidion properans|ZFMK_COL_2011_177
FBCOC054-10|Bembidion properans|BC ZSM COLA 00244
EUCAR964-11|Bembidion properans|ZFMK_COL_2011_178
GBMIN41168-14|Bembidion properans|GU347114
GBCOU8585-14|Bembidion properans|GBOL_Col_FK_09250
GBMIN41171-14|Bembidion properans|GU347117
FBCOI582-12|Bembidion properans|BFB_Col_FK_8562
EUCAR1483-15|Bembidion properans|DZMB_Carabidae_0342
FBCOO978-13|Bembidion properans|BFB_Col_FK_12663
FBCOB889-10|Bembidion properans|BC ZSM COL 01744
FBCOD1141-11|Bembidion properans|BCZSM_COLA_0856
GBMIN41167-14|Bembidion properans|GU347113
EUCAR365-10|Bembidion properans|ZFMK_COL_2010_579

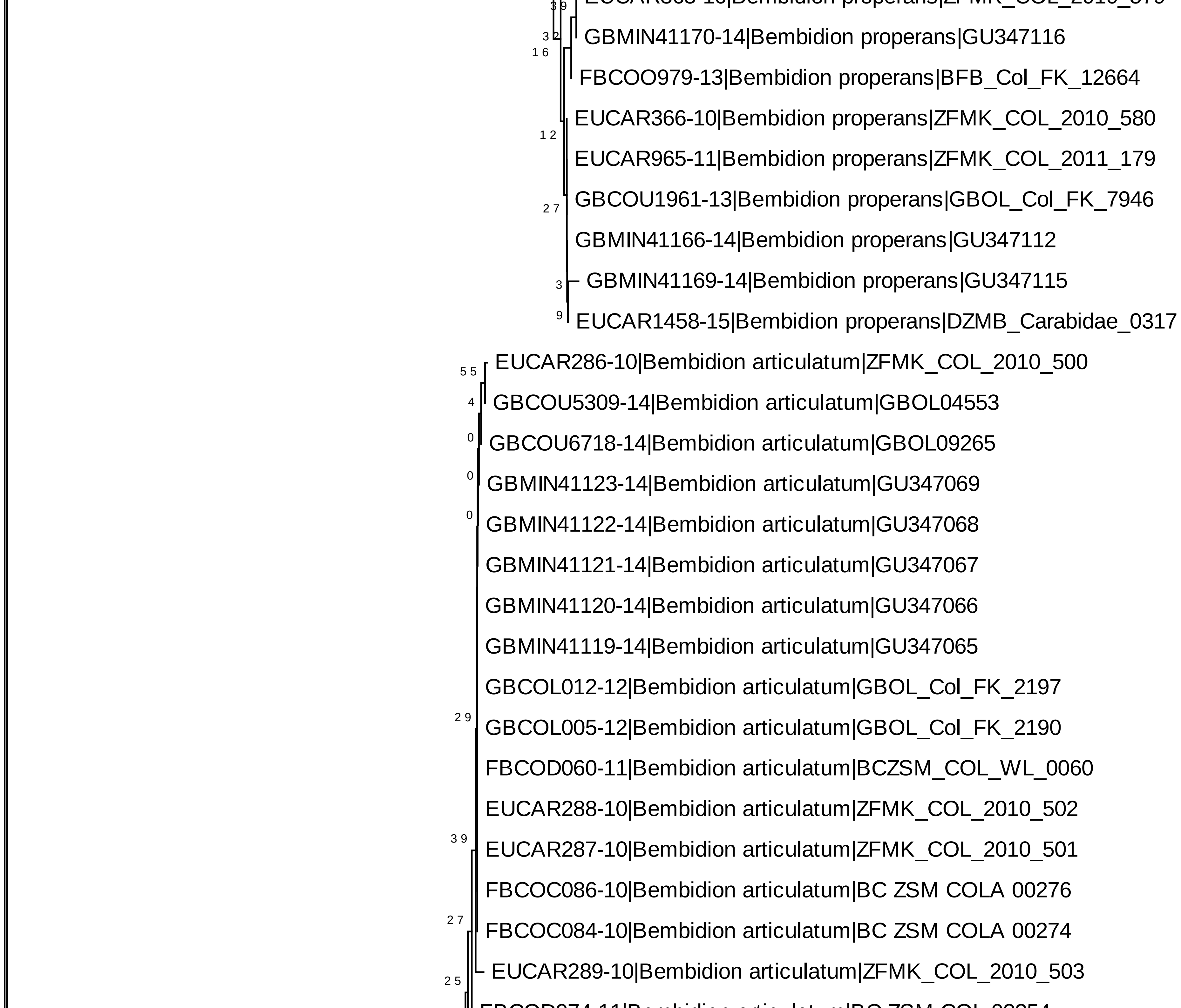
GBMIN41170-14|Bembidion properans|GU347116
FBCOO979-13|Bembidion properans|BFB_Col_FK_12664
EUCAR366-10|Bembidion properans|ZFMK_COL_2010_580
EUCAR965-11|Bembidion properans|ZFMK_COL_2011_179
GBCOU1961-13|Bembidion properans|GBOL_Col_FK_7946
GBMIN41166-14|Bembidion properans|GU347112
GBMIN41169-14|Bembidion properans|GU347115
EUCAR1458-15|Bembidion properans|DZMB_Carabidae_0317
EUCAR286-10|Bembidion articulatum|ZFMK_COL_2010_500
GBCOU5309-14|Bembidion articulatum|GBOL04553
GBCOU6718-14|Bembidion articulatum|GBOL09265
GBMIN41123-14|Bembidion articulatum|GU347069
GBMIN41122-14|Bembidion articulatum|GU347068
GBMIN41121-14|Bembidion articulatum|GU347067
GBMIN41120-14|Bembidion articulatum|GU347066
GBMIN41119-14|Bembidion articulatum|GU347065
GBCOL012-12|Bembidion articulatum|GBOL_Col_FK_2197
GBCOL005-12|Bembidion articulatum|GBOL_Col_FK_2190
FBCOD060-11|Bembidion articulatum|BCZSM_COL_WL_0060
EUCAR288-10|Bembidion articulatum|ZFMK_COL_2010_502
EUCAR287-10|Bembidion articulatum|ZFMK_COL_2010_501
FBCOC086-10|Bembidion articulatum|BC ZSM COLA 00276
FBCOC084-10|Bembidion articulatum|BC ZSM COLA 00274
EUCAR289-10|Bembidion articulatum|ZFMK_COL_2010_503

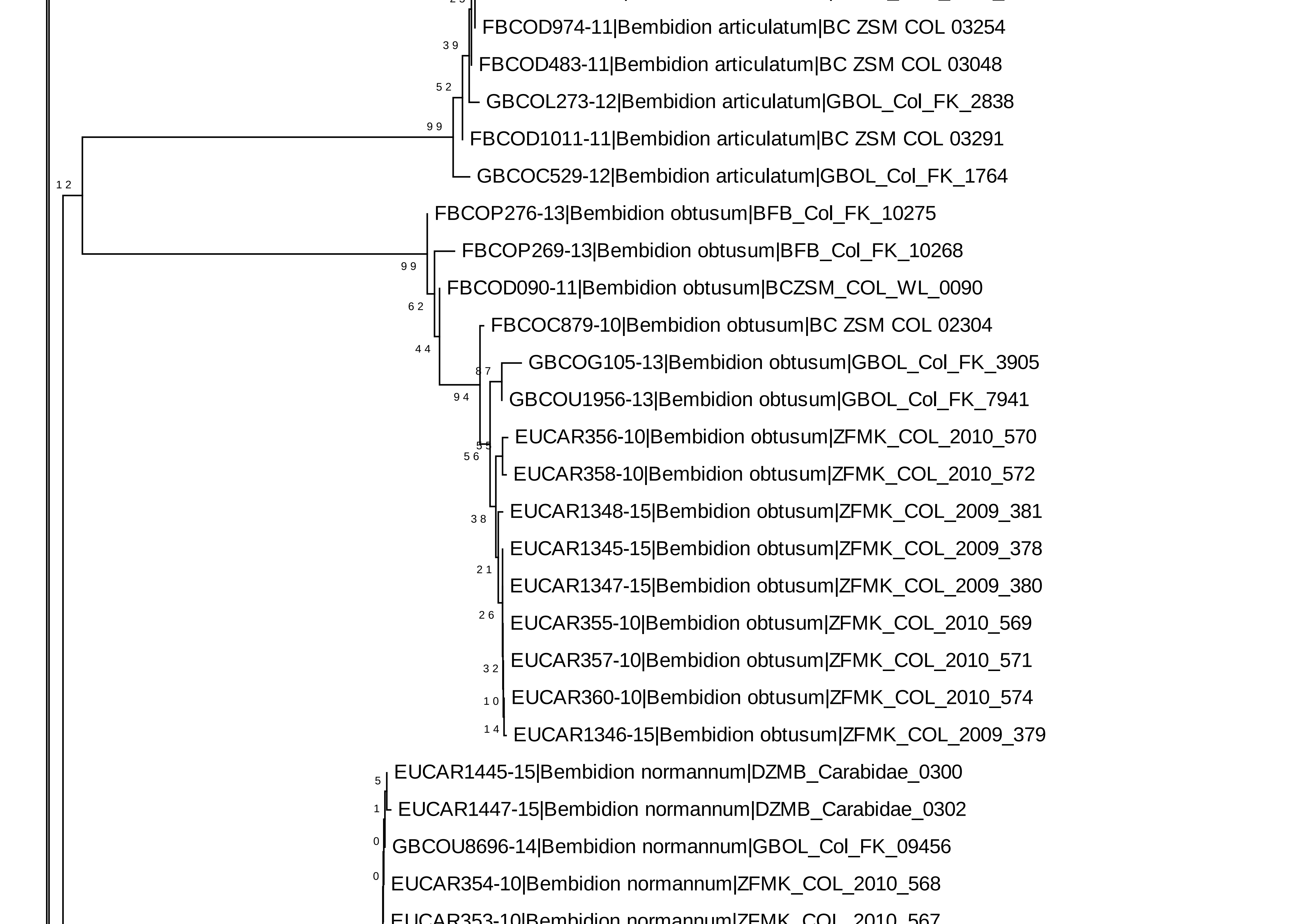

FBCOD974-11|Bembidion articulatum|BC ZSM COL 03254
FBCOD483-11|Bembidion articulatum|BC ZSM COL 03048
GBCOL273-12|Bembidion articulatum|GBOL_Col_FK_2838
FBCOD1011-11|Bembidion articulatum|BC ZSM COL 03291
GBCOC529-12|Bembidion articulatum|GBOL_Col_FK_1764
FBCOP276-13|Bembidion obtusum|BFB_Col_FK_10275
FBCOP269-13|Bembidion obtusum|BFB_Col_FK_10268
FBCOD090-11|Bembidion obtusum|BCZSM_COL_WL_0090
FBCOC879-10|Bembidion obtusum|BC ZSM COL 02304
GBCOG105-13|Bembidion obtusum|GBOL_Col_FK_3905
GBCOU1956-13|Bembidion obtusum|GBOL_Col_FK_7941
EUCAR356-10|Bembidion obtusum|ZFMK_COL_2010_570
EUCAR358-10|Bembidion obtusum|ZFMK_COL_2010_572
EUCAR1348-15|Bembidion obtusum|ZFMK_COL_2009_381
EUCAR1345-15|Bembidion obtusum|ZFMK_COL_2009_378
EUCAR1347-15|Bembidion obtusum|ZFMK_COL_2009_380
EUCAR355-10|Bembidion obtusum|ZFMK_COL_2010_569
EUCAR357-10|Bembidion obtusum|ZFMK_COL_2010_571
EUCAR360-10|Bembidion obtusum|ZFMK_COL_2010_574
EUCAR1346-15|Bembidion obtusum|ZFMK_COL_2009_379
EUCAR1445-15|Bembidion normannum|DZMB_Carabidae_0300
EUCAR1447-15|Bembidion normannum|DZMB_Carabidae_0302
GBCOU8696-14|Bembidion normannum|GBOL_Col_FK_09456
EUCAR354-10|Bembidion normannum|ZFMK_COL_2010_568

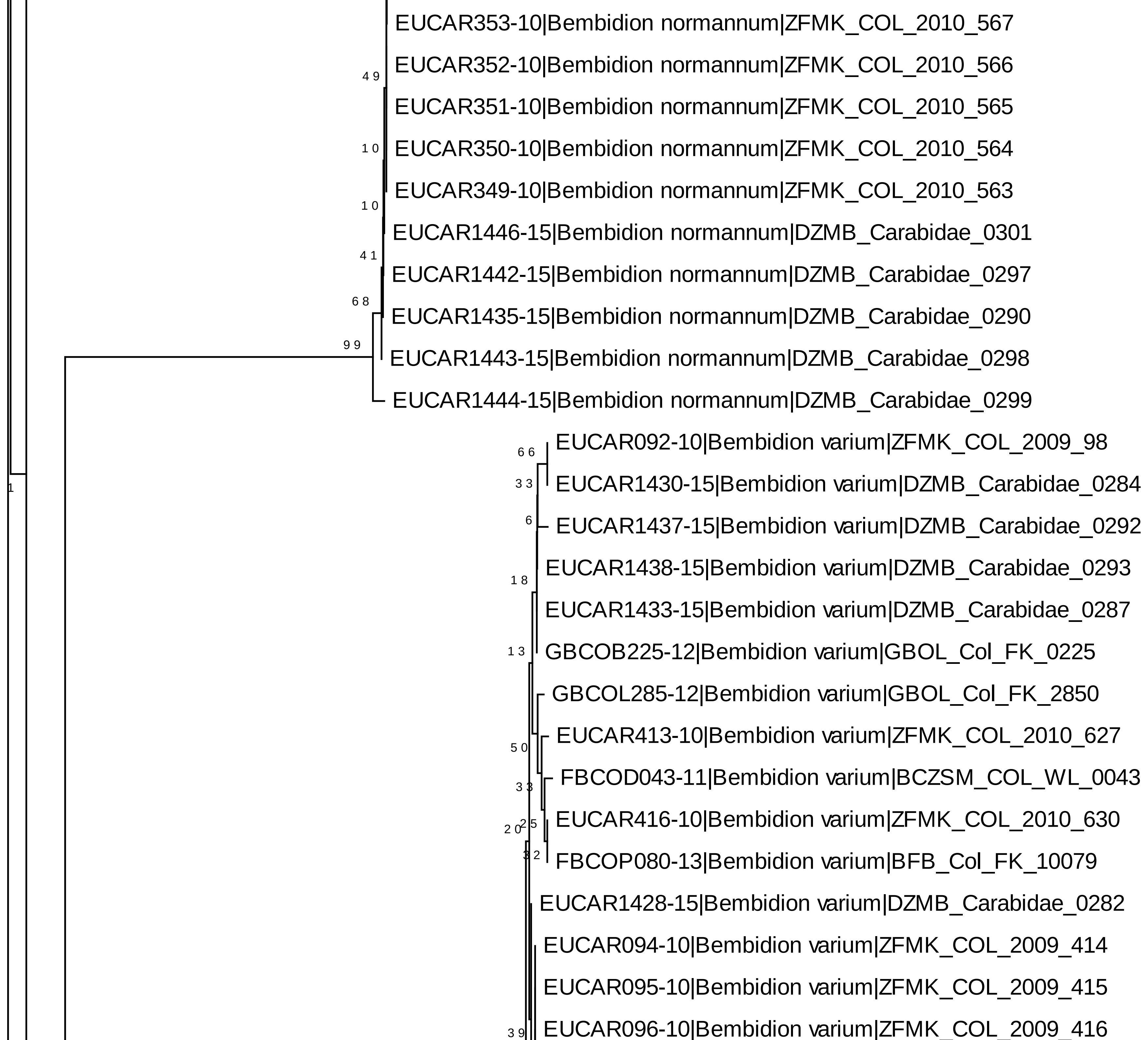
EUCAR353-10|Bembidion normannum|ZFMK_COL_2010_567
EUCAR352-10|Bembidion normannum|ZFMK_COL_2010_566
EUCAR351-10|Bembidion normannum|ZFMK_COL_2010_565
EUCAR350-10|Bembidion normannum|ZFMK_COL_2010_564
EUCAR349-10|Bembidion normannum|ZFMK_COL_2010_563
EUCAR1446-15|Bembidion normannum|DZMB_Carabidae_0301
EUCAR1442-15|Bembidion normannum|DZMB_Carabidae_0297
EUCAR1435-15|Bembidion normannum|DZMB_Carabidae_0290
EUCAR1443-15|Bembidion normannum|DZMB_Carabidae_0298
EUCAR1444-15|Bembidion normannum|DZMB_Carabidae_0299
EUCAR092-10|Bembidion varium|ZFMK_COL_2009_98
EUCAR1430-15|Bembidion varium|DZMB_Carabidae_0284
EUCAR1437-15|Bembidion varium|DZMB_Carabidae_0292
EUCAR1438-15|Bembidion varium|DZMB_Carabidae_0293
EUCAR1433-15|Bembidion varium|DZMB_Carabidae_0287
GBCOB225-12|Bembidion varium|GBOL_Col_FK_0225
GBCOL285-12|Bembidion varium|GBOL_Col_FK_2850
EUCAR413-10|Bembidion varium|ZFMK_COL_2010_627
FBCOD043-11|Bembidion varium|BCZSM_COL_WL_0043
EUCAR416-10|Bembidion varium|ZFMK_COL_2010_630
FBCOP080-13|Bembidion varium|BFB_Col_FK_10079
EUCAR1428-15|Bembidion varium|DZMB_Carabidae_0282
EUCAR094-10|Bembidion varium|ZFMK_COL_2009_414
EUCAR095-10|Bembidion varium|ZFMK_COL_2009_415
EUCAR096-10|Bembidion varium|ZFMK_COL_2009_416

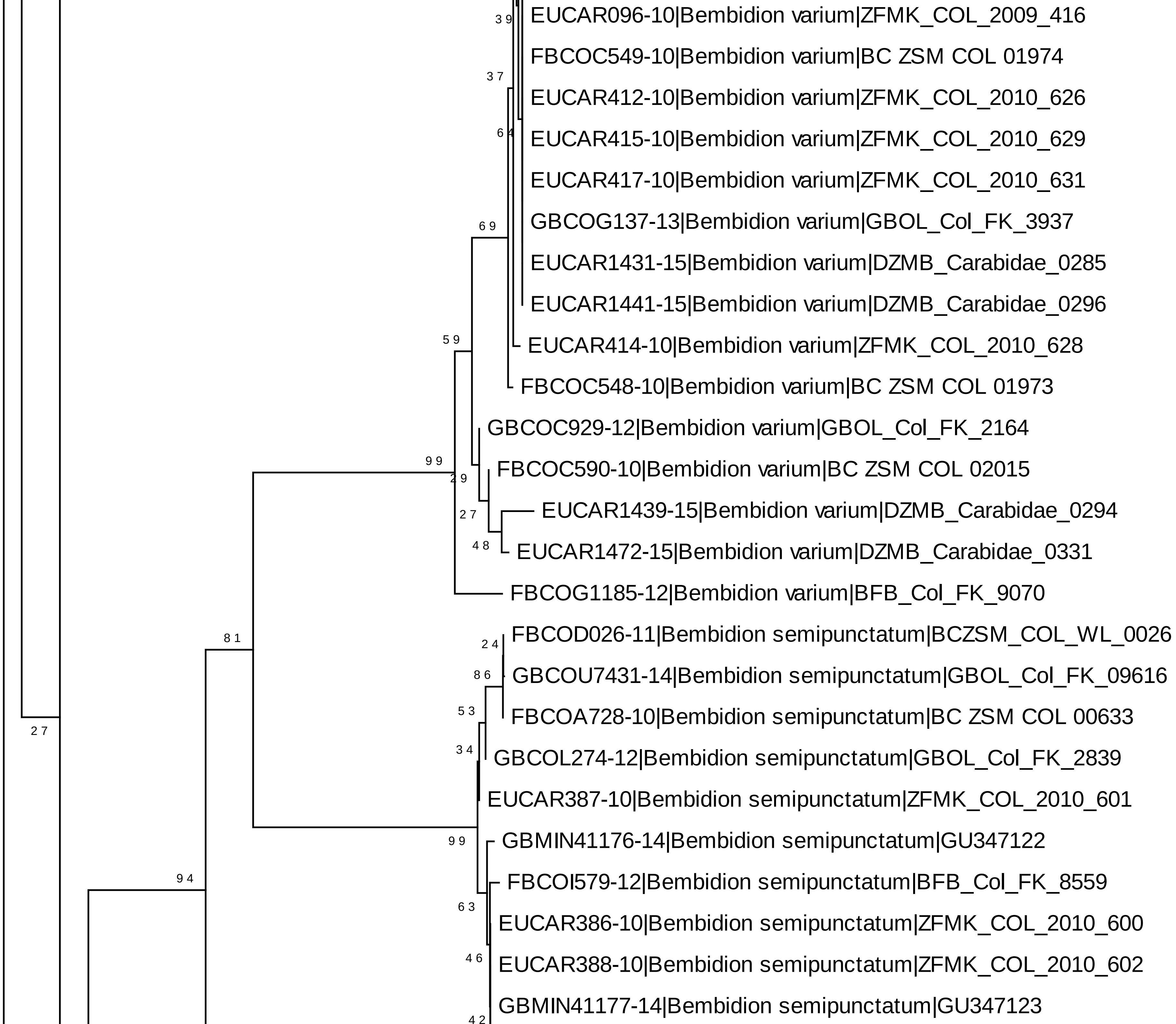
EUCAR096-10|Bembidion varium|ZFMK_COL_2009_416
FBCOC549-10|Bembidion varium|BC ZSM COL 01974
EUCAR412-10|Bembidion varium|ZFMK_COL_2010_626
EUCAR415-10|Bembidion varium|ZFMK_COL_2010_629
EUCAR417-10|Bembidion varium|ZFMK_COL_2010_631
GBCOG137-13|Bembidion varium|GBOL_Col_FK_3937
EUCAR1431-15|Bembidion varium|DZMB_Carabidae_0285
EUCAR1441-15|Bembidion varium|DZMB_Carabidae_0296
EUCAR414-10|Bembidion varium|ZFMK_COL_2010_628
FBCOC548-10|Bembidion varium|BC ZSM COL 01973
GBCOC929-12|Bembidion varium|GBOL_Col_FK_2164
FBCOC590-10|Bembidion varium|BC ZSM COL 02015
EUCAR1439-15|Bembidion varium|DZMB_Carabidae_0294
EUCAR1472-15|Bembidion varium|DZMB_Carabidae_0331
FBCOG1185-12|Bembidion varium|BFB_Col_FK_9070
FBCOD026-11|Bembidion semipunctatum|BCZSM_COL_WL_0026
GBCOU7431-14|Bembidion semipunctatum|GBOL_Col_FK_09616
FBCOA728-10|Bembidion semipunctatum|BC ZSM COL 00633
GBCOL274-12|Bembidion semipunctatum|GBOL_Col_FK_2839
EUCAR387-10|Bembidion semipunctatum|ZFMK_COL_2010_601
GBMIN41176-14|Bembidion semipunctatum|GU347122
FBCOI579-12|Bembidion semipunctatum|BFB_Col_FK_8559
EUCAR386-10|Bembidion semipunctatum|ZFMK_COL_2010_600
EUCAR388-10|Bembidion semipunctatum|ZFMK_COL_2010_602
GBMIN41177-14|Bembidion semipunctatum|GU347123
39
37
64
69
59
99
29
27
48
81
24
86
53
34
99
63
46
42
94
27

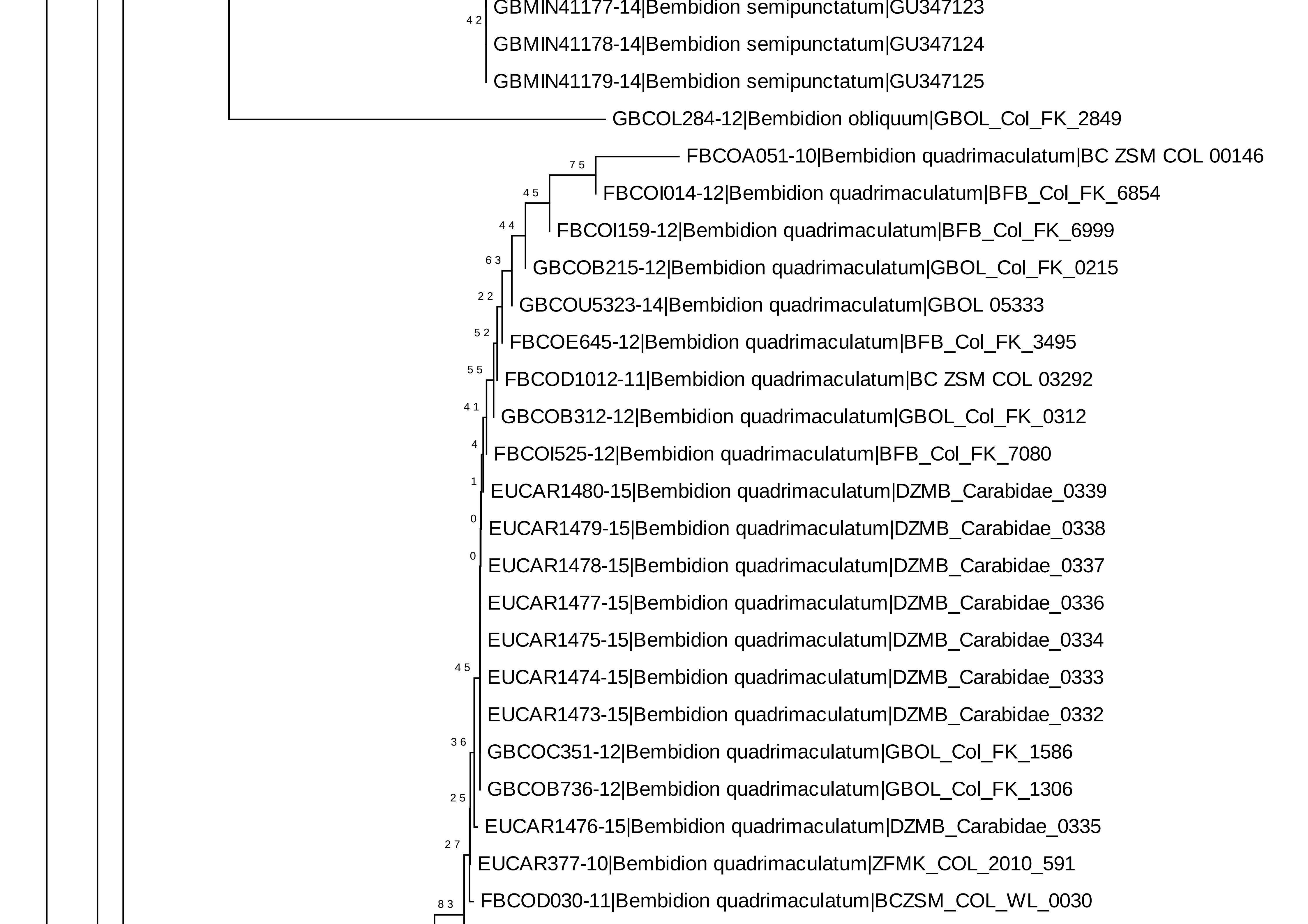
GBMIN41177-14|Bembidion semipunctatum|GU347123
GBMIN41178-14|Bembidion semipunctatum|GU347124
GBMIN41179-14|Bembidion semipunctatum|GU347125
GBCOL284-12|Bembidion obliquum|GBOL_Col_FK_2849
FBCOA051-10|Bembidion quadrimaculatum|BC ZSM COL 00146
FBCOI014-12|Bembidion quadrimaculatum|BFB_Col_FK_6854
FBCOI159-12|Bembidion quadrimaculatum|BFB_Col_FK_6999
GBCOB215-12|Bembidion quadrimaculatum|GBOL_Col_FK_0215
GBCOU5323-14|Bembidion quadrimaculatum|GBOL 05333
FBCOE645-12|Bembidion quadrimaculatum|BFB_Col_FK_3495
FBCOD1012-11|Bembidion quadrimaculatum|BC ZSM COL 03292
GBCOB312-12|Bembidion quadrimaculatum|GBOL_Col_FK_0312
FBCOI525-12|Bembidion quadrimaculatum|BFB_Col_FK_7080
EUCAR1480-15|Bembidion quadrimaculatum|DZMB_Carabidae_0339
EUCAR1479-15|Bembidion quadrimaculatum|DZMB_Carabidae_0338
EUCAR1478-15|Bembidion quadrimaculatum|DZMB_Carabidae_0337
EUCAR1477-15|Bembidion quadrimaculatum|DZMB_Carabidae_0336
EUCAR1475-15|Bembidion quadrimaculatum|DZMB_Carabidae_0334
EUCAR1474-15|Bembidion quadrimaculatum|DZMB_Carabidae_0333
EUCAR1473-15|Bembidion quadrimaculatum|DZMB_Carabidae_0332
GBCOC351-12|Bembidion quadrimaculatum|GBOL_Col_FK_1586
GBCOB736-12|Bembidion quadrimaculatum|GBOL_Col_FK_1306
EUCAR1476-15|Bembidion quadrimaculatum|DZMB_Carabidae_0335
EUCAR377-10|Bembidion quadrimaculatum|ZFMK_COL_2010_591
FBCOD030-11|Bembidion quadrimaculatum|BCZSM_COL_WL_0030

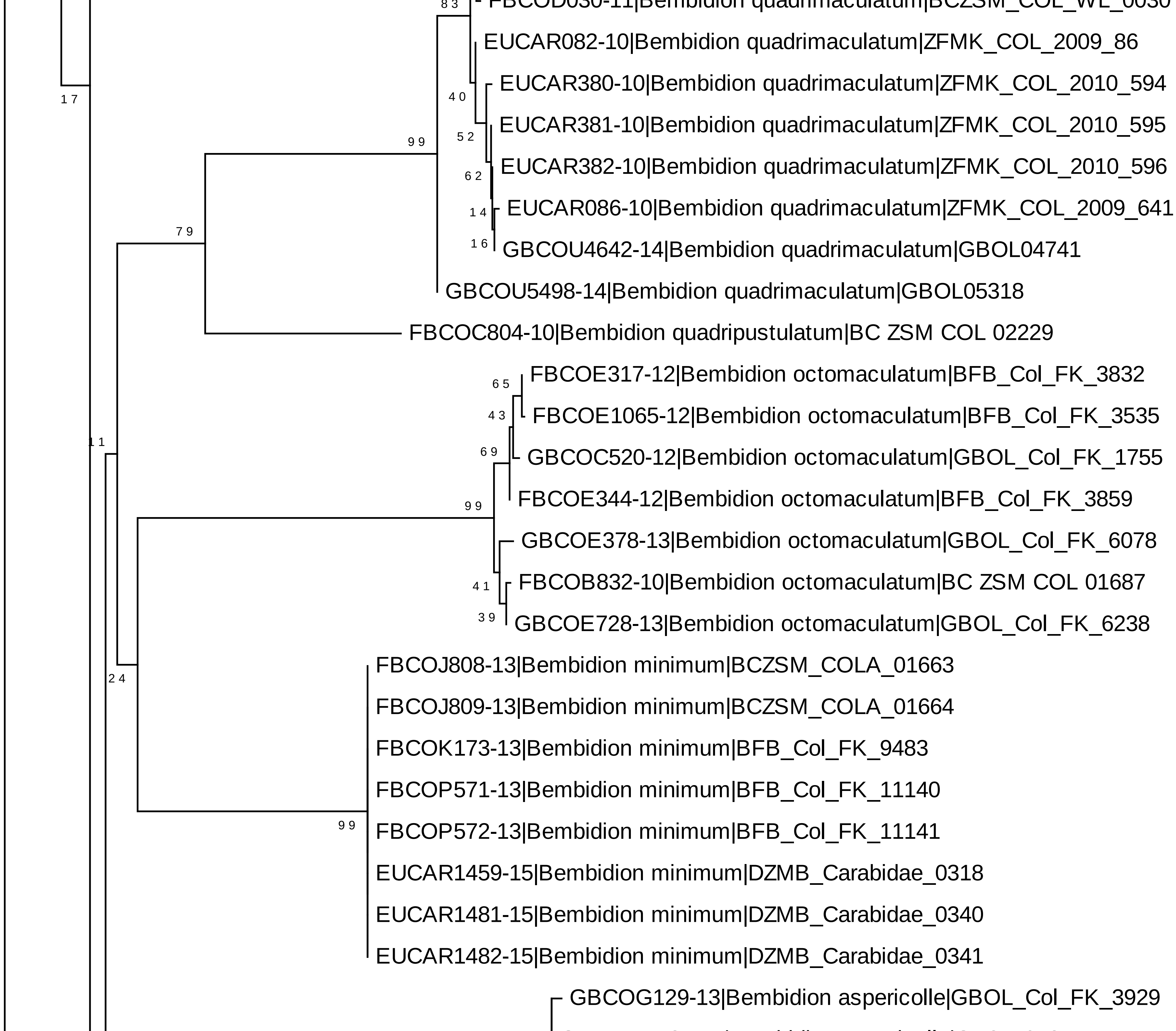

EUCAR082-10|Bembidion quadrimaculatum|ZFMK_COL_2009_86
EUCAR380-10|Bembidion quadrimaculatum|ZFMK_COL_2010_594
EUCAR381-10|Bembidion quadrimaculatum|ZFMK_COL_2010_595
EUCAR382-10|Bembidion quadrimaculatum|ZFMK_COL_2010_596
EUCAR086-10|Bembidion quadrimaculatum|ZFMK_COL_2009_641
GBCOU4642-14|Bembidion quadrimaculatum|GBOL04741
GBCOU5498-14|Bembidion quadrimaculatum|GBOL05318
FBCOC804-10|Bembidion quadripustulatum|BC ZSM COL 02229
FBCOE317-12|Bembidion octomaculatum|BFB_Col_FK_3832
FBCOE1065-12|Bembidion octomaculatum|BFB_Col_FK_3535
GBCOC520-12|Bembidion octomaculatum|GBOL_Col_FK_1755
FBCOE344-12|Bembidion octomaculatum|BFB_Col_FK_3859
GBCOE378-13|Bembidion octomaculatum|GBOL_Col_FK_6078
FBCOB832-10|Bembidion octomaculatum|BC ZSM COL 01687
GBCOE728-13|Bembidion octomaculatum|GBOL_Col_FK_6238
FBCOJ808-13|Bembidion minimum|BCZSM_COLA_01663
FBCOJ809-13|Bembidion minimum|BCZSM_COLA_01664
FBCOK173-13|Bembidion minimum|BFB_Col_FK_9483
FBCOP571-13|Bembidion minimum|BFB_Col_FK_11140
FBCOP572-13|Bembidion minimum|BFB_Col_FK_11141
EUCAR1459-15|Bembidion minimum|DZMB_Carabidae_0318
EUCAR1481-15|Bembidion minimum|DZMB_Carabidae_0340
EUCAR1482-15|Bembidion minimum|DZMB_Carabidae_0341
GBCOG129-13|Bembidion aspericolle|GBOL_Col_FK_3929

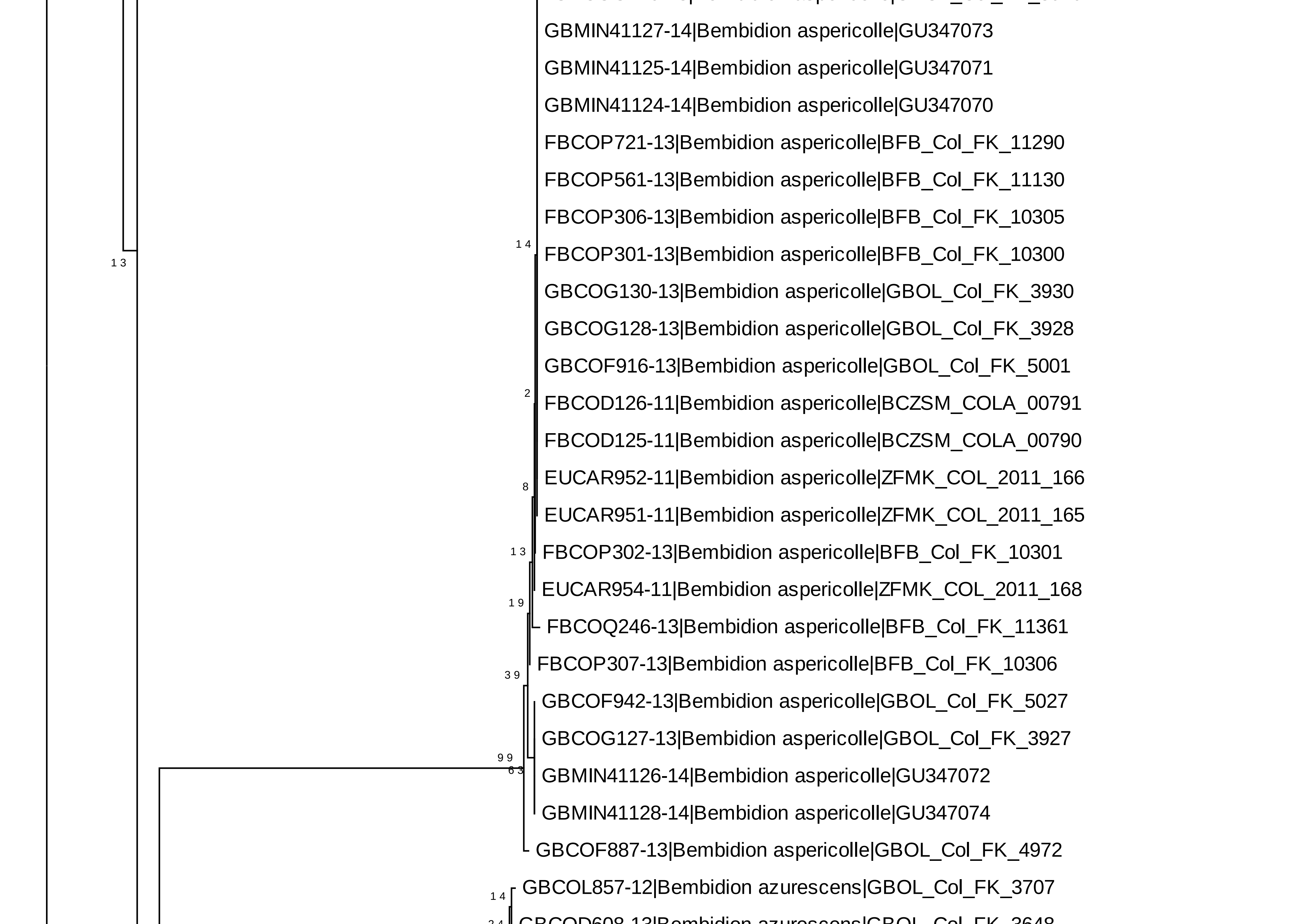
GBMIN41127-14|Bembidion aspericolle|GU347073
GBMIN41125-14|Bembidion aspericolle|GU347071
GBMIN41124-14|Bembidion aspericolle|GU347070
FBCOP721-13|Bembidion aspericolle|BFB_Col_FK_11290
FBCOP561-13|Bembidion aspericolle|BFB_Col_FK_11130
FBCOP306-13|Bembidion aspericolle|BFB_Col_FK_10305
14
FBCOP301-13|Bembidion aspericolle|BFB_Col_FK_10300
13
GBCOG130-13|Bembidion aspericolle|GBOL_Col_FK_3930
GBCOG128-13|Bembidion aspericolle|GBOL_Col_FK_3928
GBCOF916-13|Bembidion aspericolle|GBOL_Col_FK_5001
2
FBCOD126-11|Bembidion aspericolle|BCZSM_COLA_00791
FBCOD125-11|Bembidion aspericolle|BCZSM_COLA_00790
EUCAR952-11|Bembidion aspericolle|ZFMK_COL_2011_166
8
EUCAR951-11|Bembidion aspericolle|ZFMK_COL_2011_165
13
FBCOP302-13|Bembidion aspericolle|BFB_Col_FK_10301
EUCAR954-11|Bembidion aspericolle|ZFMK_COL_2011_168
19
FBCOQ246-13|Bembidion aspericolle|BFB_Col_FK_11361
FBCOP307-13|Bembidion aspericolle|BFB_Col_FK_10306
39
GBCOF942-13|Bembidion aspericolle|GBOL_Col_FK_5027
GBCOG127-13|Bembidion aspericolle|GBOL_Col_FK_3927
99
63
GBMIN41126-14|Bembidion aspericolle|GU347072
GBMIN41128-14|Bembidion aspericolle|GU347074
GBCOF887-13|Bembidion aspericolle|GBOL_Col_FK_4972
14
GBCOL857-12|Bembidion azurescens|GBOL_Col_FK_3707

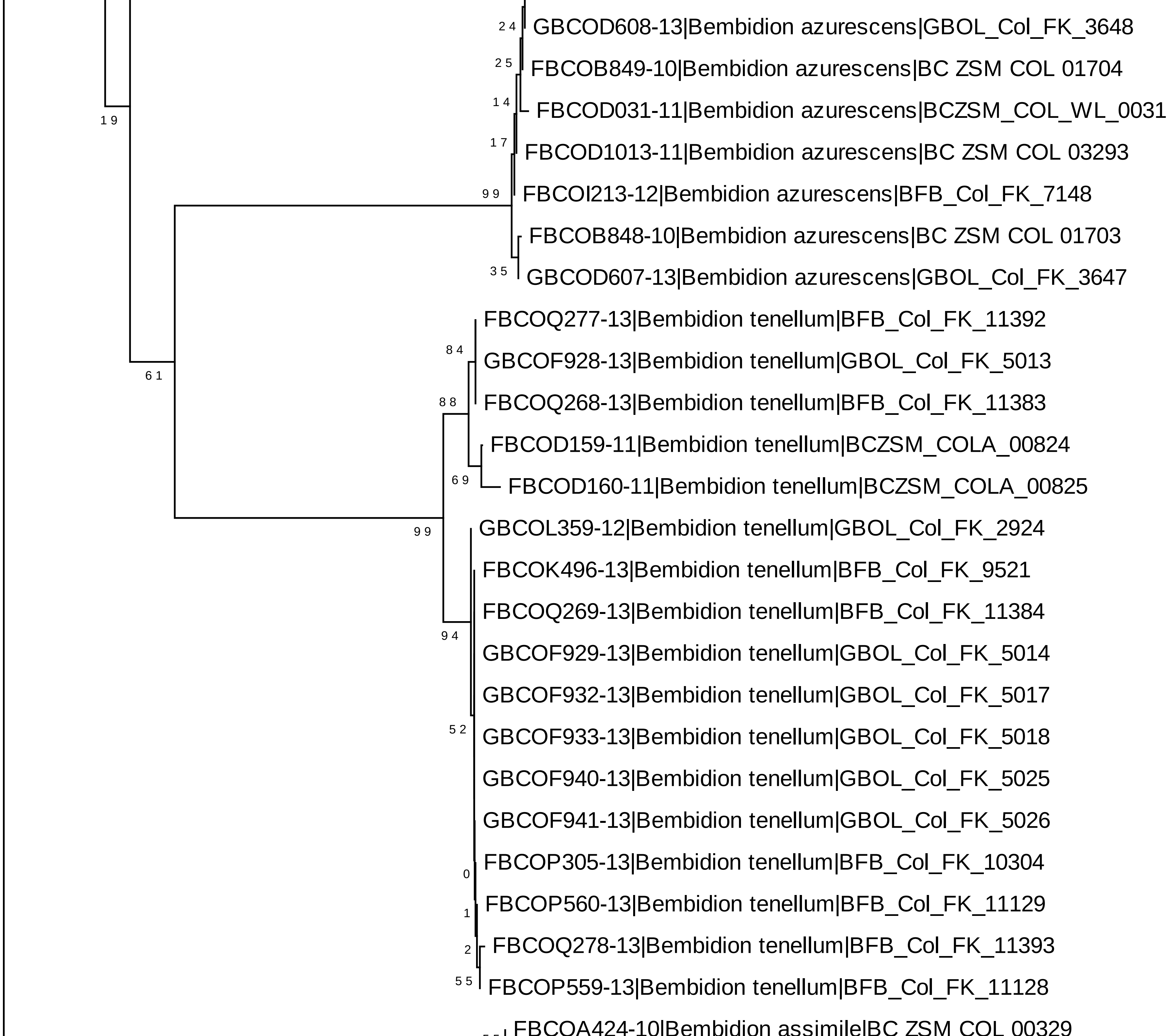
24
GBCOD608-13|Bembidion azurescens|GBOL_Col_FK_3648
25
FBCOB849-10|Bembidion azurescens|BC ZSM COL 01704
19
14
FBCOD031-11|Bembidion azurescens|BCZSM_COL_WL_0031
17
FBCOD1013-11|Bembidion azurescens|BC ZSM COL 03293
99
FBCOI213-12|Bembidion azurescens|BFB_Col_FK_7148
FBCOB848-10|Bembidion azurescens|BC ZSM COL 01703
35
GBCOD607-13|Bembidion azurescens|GBOL_Col_FK_3647
FBCOQ277-13|Bembidion tenellum|BFB_Col_FK_11392
84
GBCOF928-13|Bembidion tenellum|GBOL_Col_FK_5013
61
88
FBCOQ268-13|Bembidion tenellum|BFB_Col_FK_11383
FBCOD159-11|Bembidion tenellum|BCZSM_COLA_00824
69
FBCOD160-11|Bembidion tenellum|BCZSM_COLA_00825
GBCOL359-12|Bembidion tenellum|GBOL_Col_FK_2924
99
FBCOK496-13|Bembidion tenellum|BFB_Col_FK_9521
FBCOQ269-13|Bembidion tenellum|BFB_Col_FK_11384
94
GBCOF929-13|Bembidion tenellum|GBOL_Col_FK_5014
GBCOF932-13|Bembidion tenellum|GBOL_Col_FK_5017
52
GBCOF933-13|Bembidion tenellum|GBOL_Col_FK_5018
GBCOF940-13|Bembidion tenellum|GBOL_Col_FK_5025
GBCOF941-13|Bembidion tenellum|GBOL_Col_FK_5026
FBCOP305-13|Bembidion tenellum|BFB_Col_FK_10304
0
FBCOP560-13|Bembidion tenellum|BFB_Col_FK_11129
1
FBCOQ278-13|Bembidion tenellum|BFB_Col_FK_11393
2
55
FBCOP559-13|Bembidion tenellum|BFB_Col_FK_11128
FBCOA424-10|Bembidion assimile|BC ZSM COL 00329

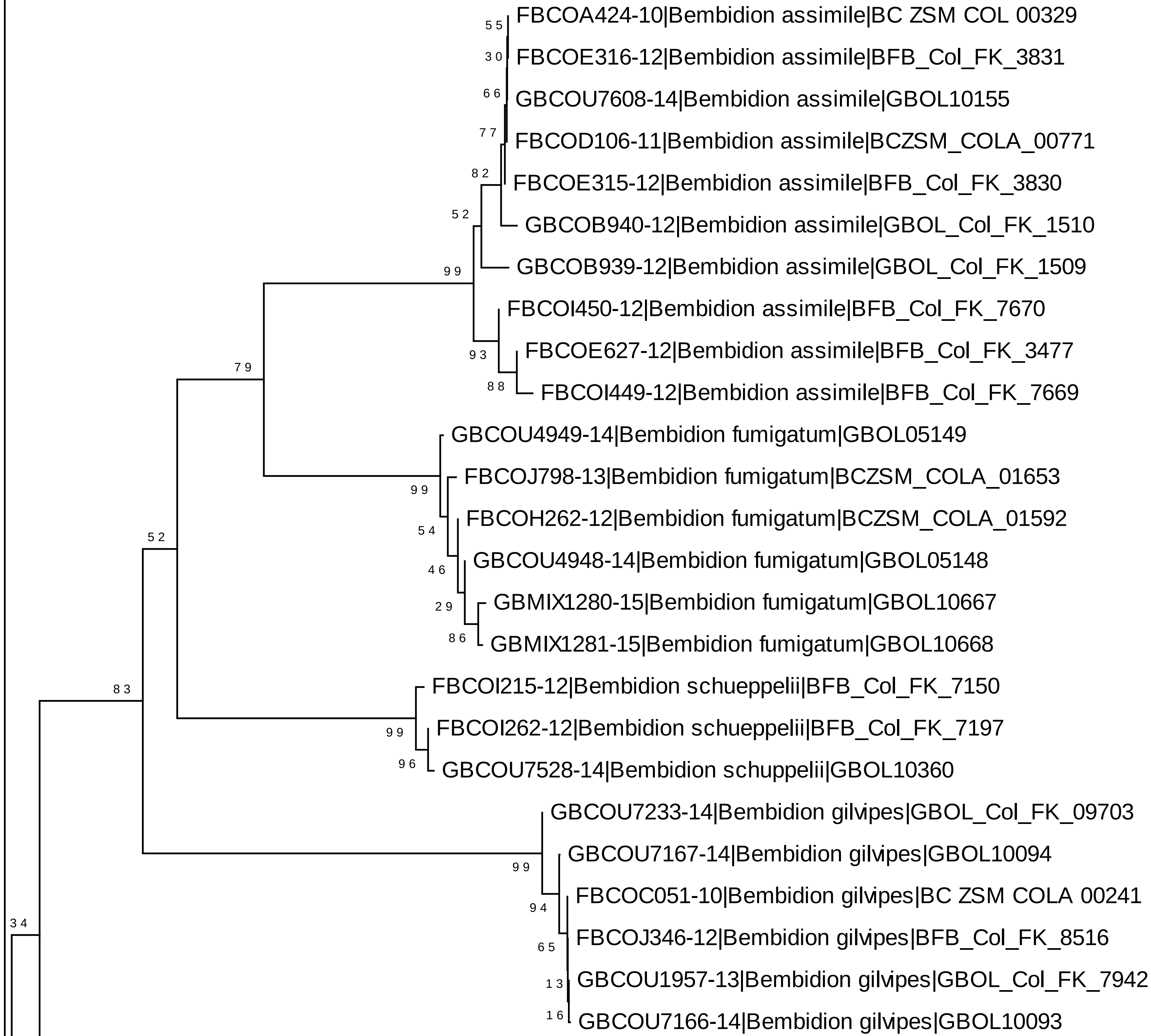

FBCOA424-10|Bembidion assimile|BC ZSM COL 00329
FBCOE316-12|Bembidion assimile|BFB_Col_FK_3831
GBCOU7608-14|Bembidion assimile|GBOL10155
FBCOD106-11|Bembidion assimile|BCZSM_COLA_00771
FBCOE315-12|Bembidion assimile|BFB_Col_FK_3830
GBCOB940-12|Bembidion assimile|GBOL_Col_FK_1510
GBCOB939-12|Bembidion assimile|GBOL_Col_FK_1509
FBCOI450-12|Bembidion assimile|BFB_Col_FK_7670
FBCOE627-12|Bembidion assimile|BFB_Col_FK_3477
FBCOI449-12|Bembidion assimile|BFB_Col_FK_7669
GBCOU4949-14|Bembidion fumigatum|GBOL05149
FBCOJ798-13|Bembidion fumigatum|BCZSM_COLA_01653
FBCOH262-12|Bembidion fumigatum|BCZSM_COLA_01592
GBCOU4948-14|Bembidion fumigatum|GBOL05148
GBMIX1280-15|Bembidion fumigatum|GBOL10667
GBMIX1281-15|Bembidion fumigatum|GBOL10668
FBCOI215-12|Bembidion schueppelii|BFB_Col_FK_7150
FBCOI262-12|Bembidion schueppelii|BFB_Col_FK_7197
GBCOU7528-14|Bembidion schuppelii|GBOL10360
GBCOU7233-14|Bembidion gilvipes|GBOL_Col_FK_09703
GBCOU7167-14|Bembidion gilvipes|GBOL10094
FBCOC051-10|Bembidion gilvipes|BC ZSM COLA 00241
FBCOJ346-12|Bembidion gilvipes|BFB_Col_FK_8516
GBCOU1957-13|Bembidion gilvipes|GBOL_Col_FK_7942
GBCOU7166-14|Bembidion gilvipes|GBOL10093
55
30
66
77
82
52
99
93
88
79
99
54
46
29
86
52
99
96
83
99
94
65
13
16
34

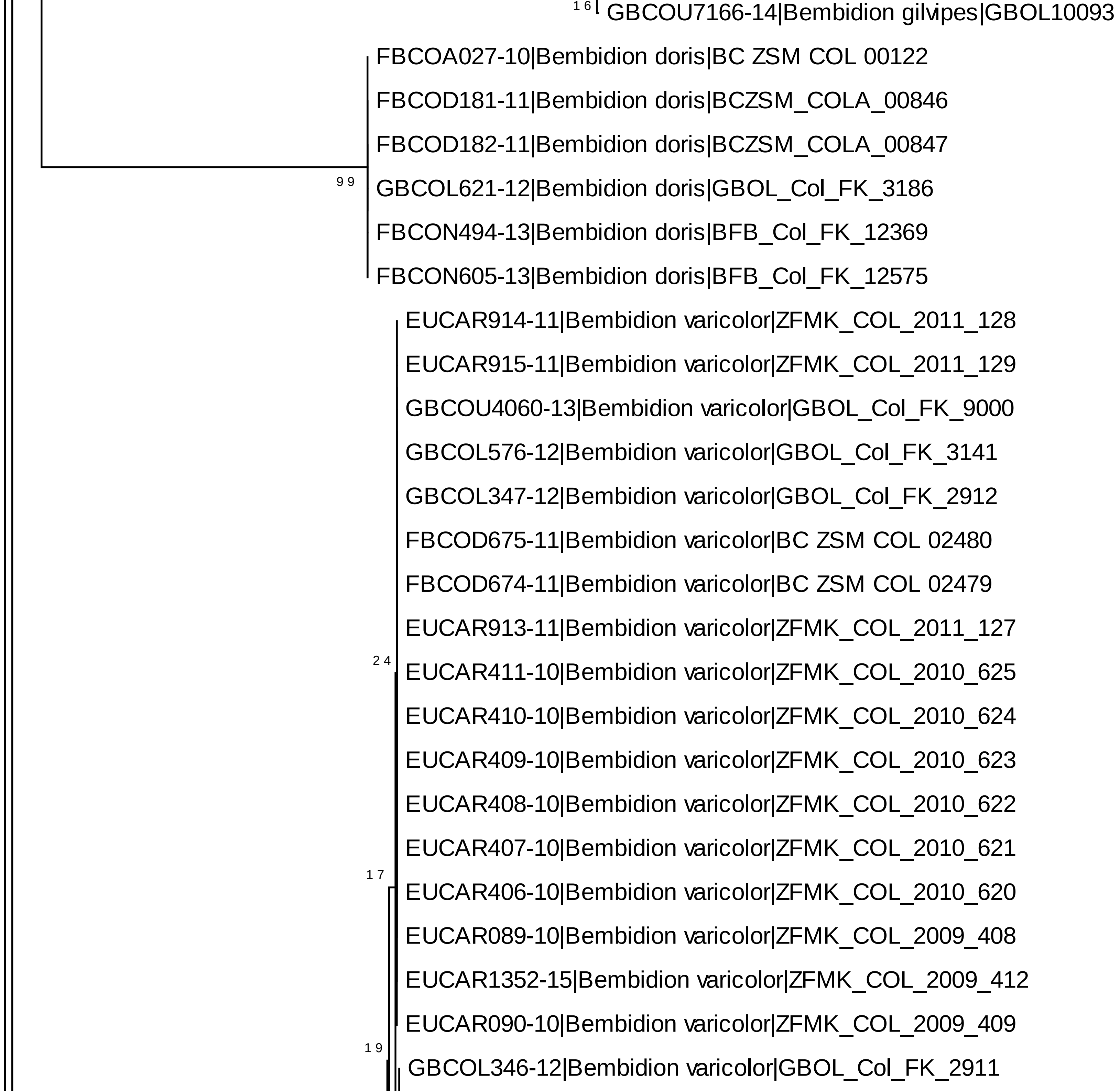
16
GBCOU7166-14|Bembidion gilvipes|GBOL10093
FBCOA027-10|Bembidion doris|BC ZSM COL 00122
FBCOD181-11|Bembidion doris|BCZSM_COLA_00846
FBCOD182-11|Bembidion doris|BCZSM_COLA_00847
99
GBCOL621-12|Bembidion doris|GBOL_Col_FK_3186
FBCON494-13|Bembidion doris|BFB_Col_FK_12369
FBCON605-13|Bembidion doris|BFB_Col_FK_12575
EUCAR914-11|Bembidion varicolor|ZFMK_COL_2011_128
EUCAR915-11|Bembidion varicolor|ZFMK_COL_2011_129
GBCOU4060-13|Bembidion varicolor|GBOL_Col_FK_9000
GBCOL576-12|Bembidion varicolor|GBOL_Col_FK_3141
GBCOL347-12|Bembidion varicolor|GBOL_Col_FK_2912
FBCOD675-11|Bembidion varicolor|BC ZSM COL 02480
FBCOD674-11|Bembidion varicolor|BC ZSM COL 02479
EUCAR913-11|Bembidion varicolor|ZFMK_COL_2011_127
24
EUCAR411-10|Bembidion varicolor|ZFMK_COL_2010_625
EUCAR410-10|Bembidion varicolor|ZFMK_COL_2010_624
EUCAR409-10|Bembidion varicolor|ZFMK_COL_2010_623
EUCAR408-10|Bembidion varicolor|ZFMK_COL_2010_622
EUCAR407-10|Bembidion varicolor|ZFMK_COL_2010_621
17
EUCAR406-10|Bembidion varicolor|ZFMK_COL_2010_620
EUCAR089-10|Bembidion varicolor|ZFMK_COL_2009_408
EUCAR1352-15|Bembidion varicolor|ZFMK_COL_2009_412
EUCAR090-10|Bembidion varicolor|ZFMK_COL_2009_409
19
GBCOL346-12|Bembidion varicolor|GBOL_Col_FK_2911

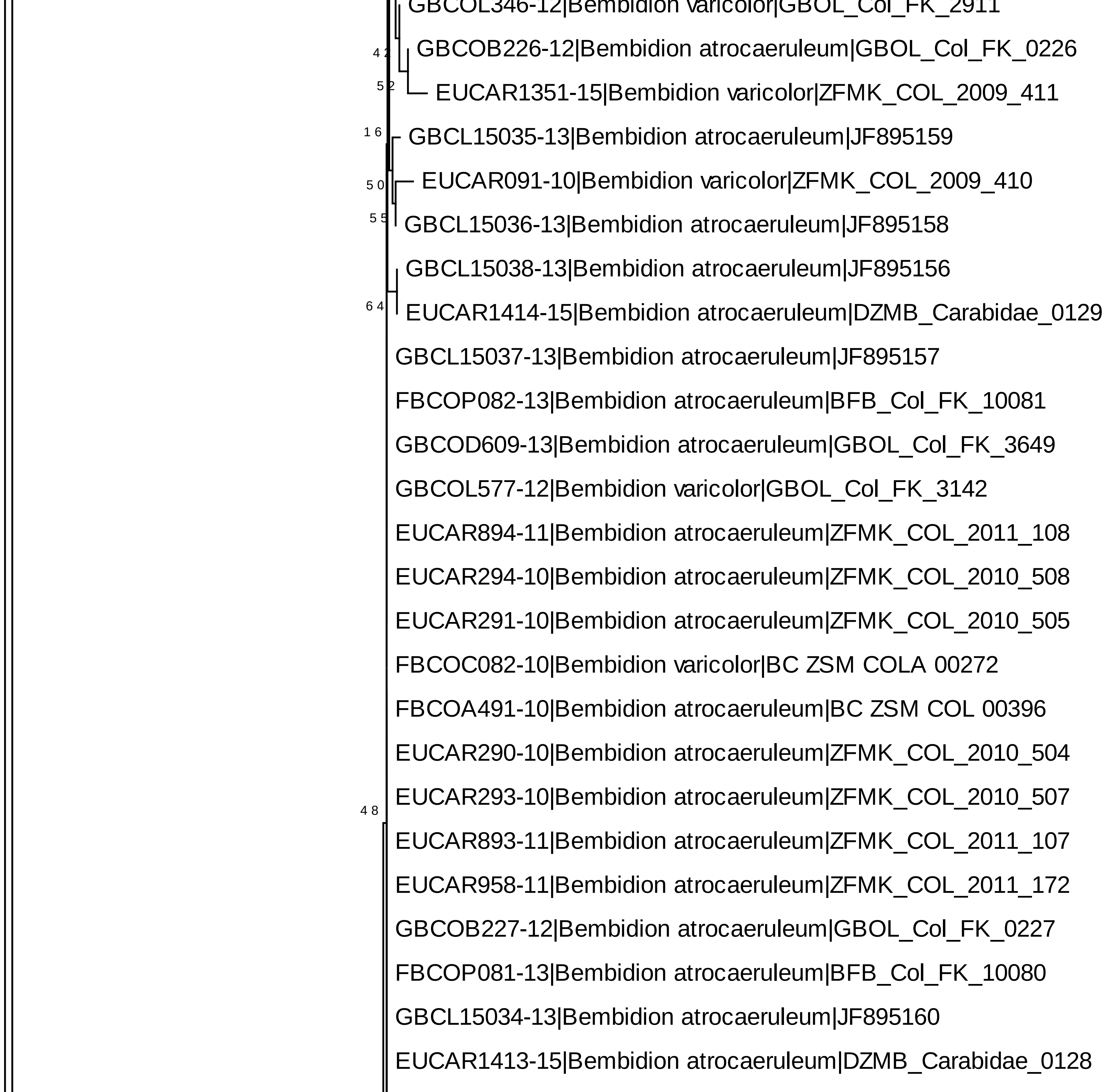
GBCOL346-12|Bembidion varicolor|GBOL_Col_FK_2911
4 2
GBCOB226-12|Bembidion atrocaeruleum|GBOL_Col_FK_0226
5 2
EUCAR1351-15|Bembidion varicolor|ZFMK_COL_2009_411
1 6
GBCL15035-13|Bembidion atrocaeruleum|JF895159
5 0
EUCAR091-10|Bembidion varicolor|ZFMK_COL_2009_410
5 5
GBCL15036-13|Bembidion atrocaeruleum|JF895158
GBCL15038-13|Bembidion atrocaeruleum|JF895156
6 4
EUCAR1414-15|Bembidion atrocaeruleum|DZMB_Carabidae_0129
GBCL15037-13|Bembidion atrocaeruleum|JF895157
FBCOP082-13|Bembidion atrocaeruleum|BFB_Col_FK_10081
GBCOD609-13|Bembidion atrocaeruleum|GBOL_Col_FK_3649
GBCOL577-12|Bembidion varicolor|GBOL_Col_FK_3142
EUCAR894-11|Bembidion atrocaeruleum|ZFMK_COL_2011_108
EUCAR294-10|Bembidion atrocaeruleum|ZFMK_COL_2010_508
EUCAR291-10|Bembidion atrocaeruleum|ZFMK_COL_2010_505
FBCOC082-10|Bembidion varicolor|BC ZSM COLA 00272
FBCOA491-10|Bembidion atrocaeruleum|BC ZSM COL 00396
EUCAR290-10|Bembidion atrocaeruleum|ZFMK_COL_2010_504
EUCAR293-10|Bembidion atrocaeruleum|ZFMK_COL_2010_507
4 8
EUCAR893-11|Bembidion atrocaeruleum|ZFMK_COL_2011_107
EUCAR958-11|Bembidion atrocaeruleum|ZFMK_COL_2011_172
GBCOB227-12|Bembidion atrocaeruleum|GBOL_Col_FK_0227
FBCOP081-13|Bembidion atrocaeruleum|BFB_Col_FK_10080
GBCL15034-13|Bembidion atrocaeruleum|JF895160
EUCAR1413-15|Bembidion atrocaeruleum|DZMB_Carabidae_0128

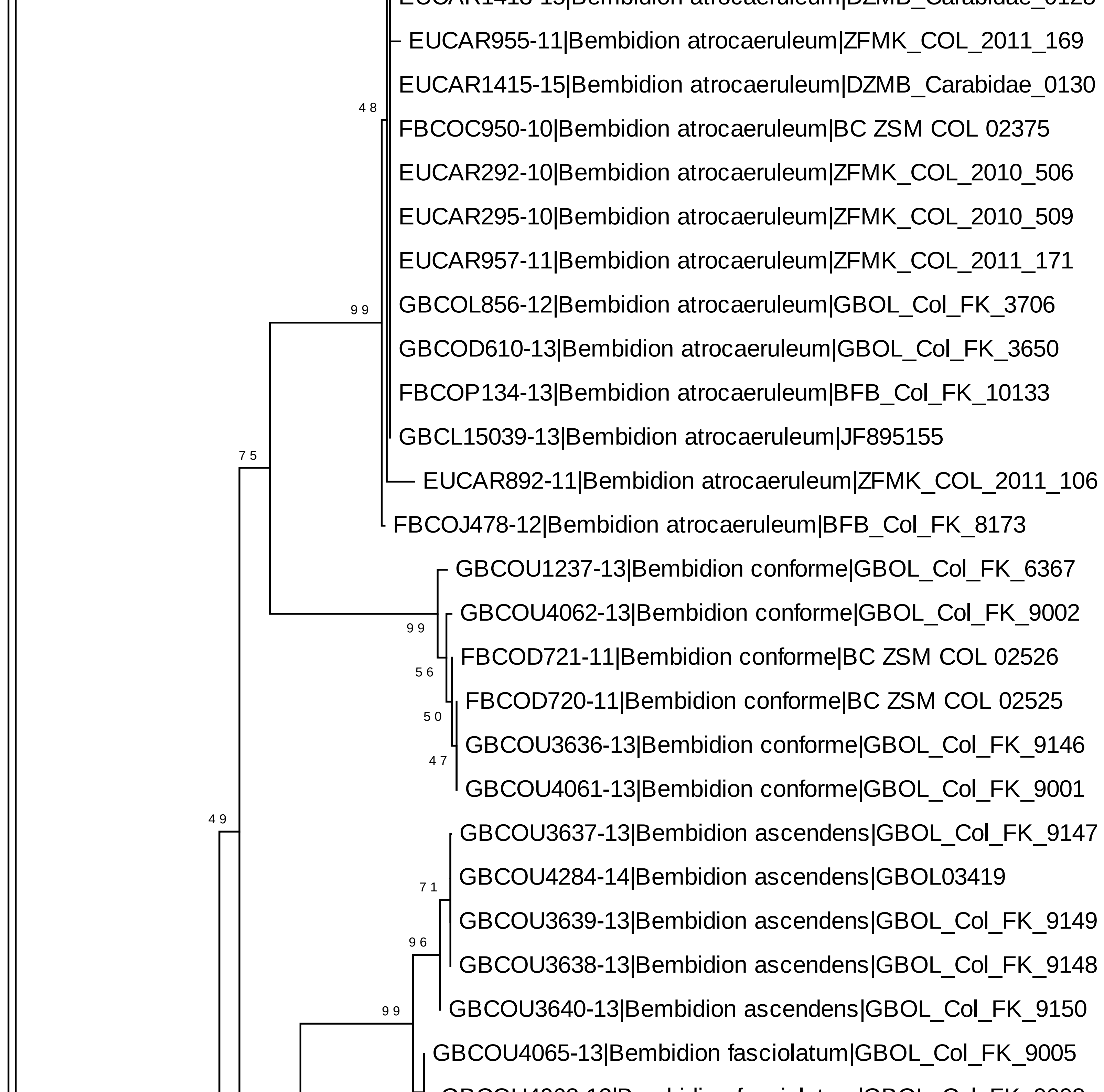

EUCAR955-11|Bembidion atrocaeruleum|ZFMK_COL_2011_169
EUCAR1415-15|Bembidion atrocaeruleum|DZMB_Carabidae_0130
48
FBCOC950-10|Bembidion atrocaeruleum|BC ZSM COL 02375
EUCAR292-10|Bembidion atrocaeruleum|ZFMK_COL_2010_506
EUCAR295-10|Bembidion atrocaeruleum|ZFMK_COL_2010_509
EUCAR957-11|Bembidion atrocaeruleum|ZFMK_COL_2011_171
99
GBCOL856-12|Bembidion atrocaeruleum|GBOL_Col_FK_3706
GBCOD610-13|Bembidion atrocaeruleum|GBOL_Col_FK_3650
FBCOP134-13|Bembidion atrocaeruleum|BFB_Col_FK_10133
GBCL15039-13|Bembidion atrocaeruleum|JF895155
75
EUCAR892-11|Bembidion atrocaeruleum|ZFMK_COL_2011_106
FBCOJ478-12|Bembidion atrocaeruleum|BFB_Col_FK_8173
GBCOU1237-13|Bembidion conforme|GBOL_Col_FK_6367
GBCOU4062-13|Bembidion conforme|GBOL_Col_FK_9002
99
FBCOD721-11|Bembidion conforme|BC ZSM COL 02526
56
FBCOD720-11|Bembidion conforme|BC ZSM COL 02525
50
GBCOU3636-13|Bembidion conforme|GBOL_Col_FK_9146
47
GBCOU4061-13|Bembidion conforme|GBOL_Col_FK_9001
49
GBCOU3637-13|Bembidion ascendens|GBOL_Col_FK_9147
GBCOU4284-14|Bembidion ascendens|GBOL03419
71
GBCOU3639-13|Bembidion ascendens|GBOL_Col_FK_9149
96
GBCOU3638-13|Bembidion ascendens|GBOL_Col_FK_9148
GBCOU3640-13|Bembidion ascendens|GBOL_Col_FK_9150
99
GBCOU4065-13|Bembidion fasciolatum|GBOL_Col_FK_9005

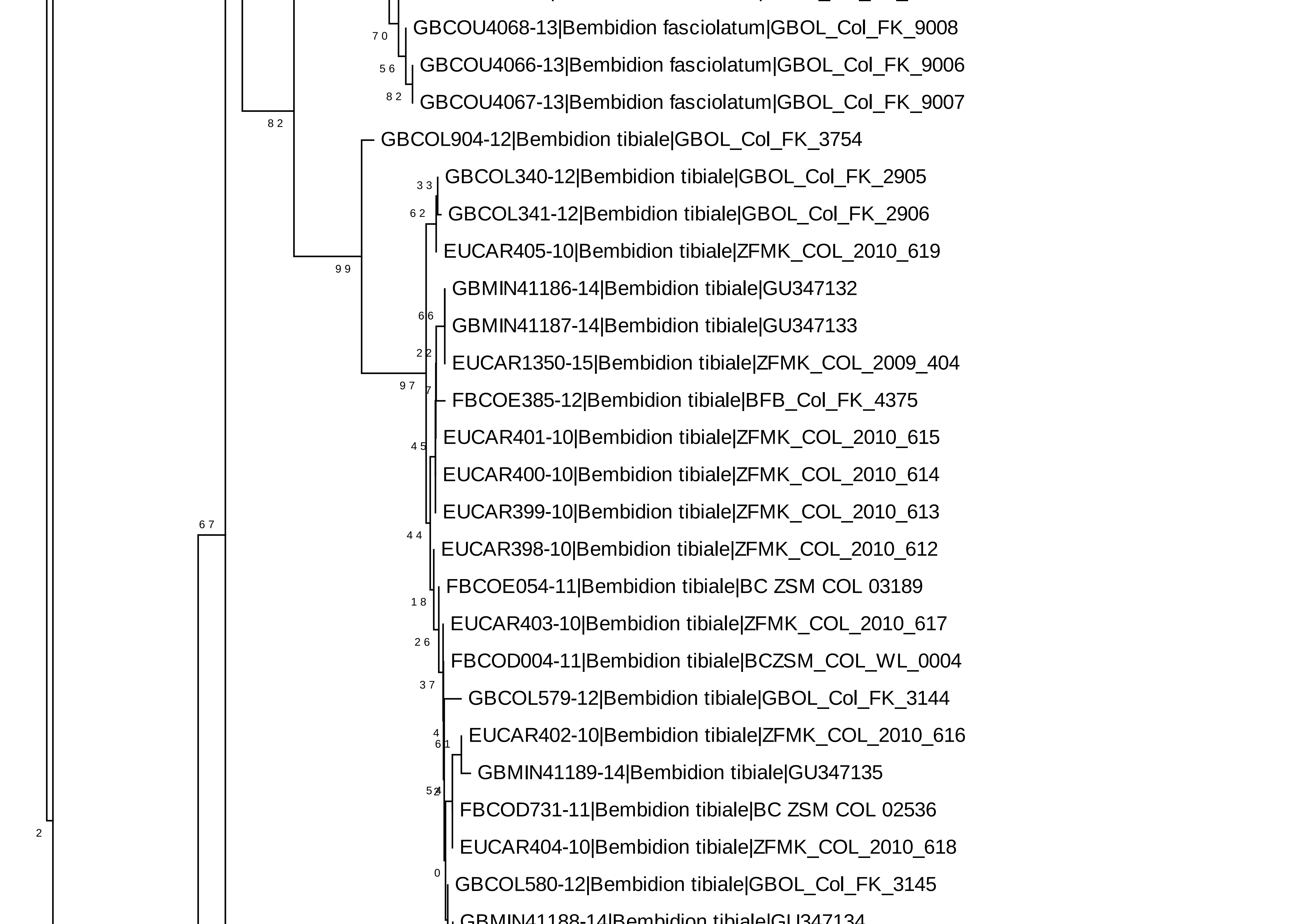
GBCOU4068-13|Bembidion fasciolatum|GBOL_Col_FK_9008
GBCOU4066-13|Bembidion fasciolatum|GBOL_Col_FK_9006
GBCOU4067-13|Bembidion fasciolatum|GBOL_Col_FK_9007
GBCOL904-12|Bembidion tibiale|GBOL_Col_FK_3754
GBCOL340-12|Bembidion tibiale|GBOL_Col_FK_2905
GBCOL341-12|Bembidion tibiale|GBOL_Col_FK_2906
EUCAR405-10|Bembidion tibiale|ZFMK_COL_2010_619
GBMIN41186-14|Bembidion tibiale|GU347132
GBMIN41187-14|Bembidion tibiale|GU347133
EUCAR1350-15|Bembidion tibiale|ZFMK_COL_2009_404
FBCOE385-12|Bembidion tibiale|BFB_Col_FK_4375
EUCAR401-10|Bembidion tibiale|ZFMK_COL_2010_615
EUCAR400-10|Bembidion tibiale|ZFMK_COL_2010_614
EUCAR399-10|Bembidion tibiale|ZFMK_COL_2010_613
EUCAR398-10|Bembidion tibiale|ZFMK_COL_2010_612
FBCOE054-11|Bembidion tibiale|BC ZSM COL 03189
EUCAR403-10|Bembidion tibiale|ZFMK_COL_2010_617
FBCOD004-11|Bembidion tibiale|BCZSM_COL_WL_0004
GBCOL579-12|Bembidion tibiale|GBOL_Col_FK_3144
EUCAR402-10|Bembidion tibiale|ZFMK_COL_2010_616
GBMIN41189-14|Bembidion tibiale|GU347135
FBCOD731-11|Bembidion tibiale|BC ZSM COL 02536
EUCAR404-10|Bembidion tibiale|ZFMK_COL_2010_618
GBCOL580-12|Bembidion tibiale|GBOL_Col_FK_3145
GBMIN41188-14|Bembidion tibiale|GU347134
70
56
82
82
33
62
99
66
22
97
7
45
67
44
18
26
37
4
61
2
0

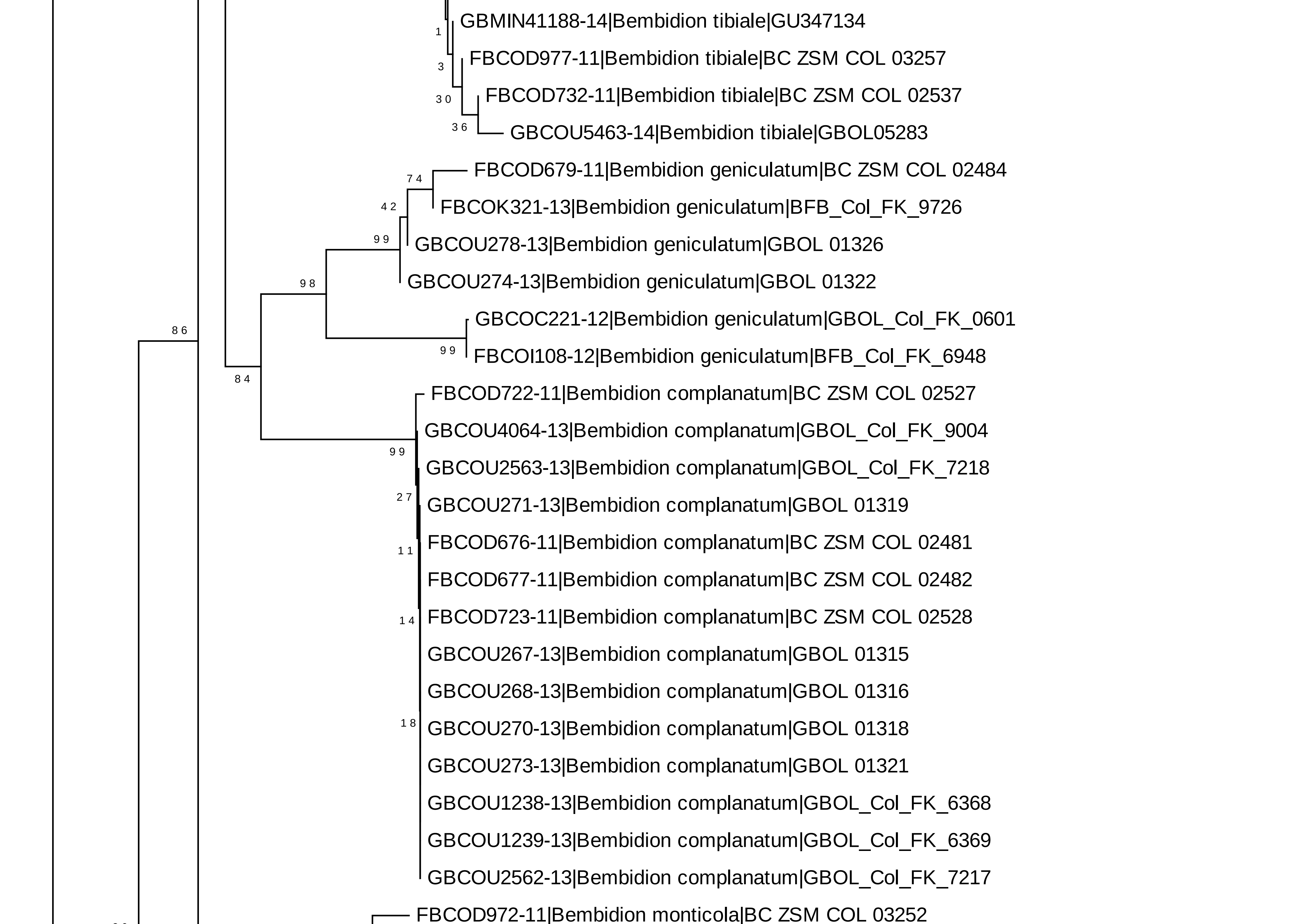
GBMIN41188-14|Bembidion tibiale|GU347134
1
FBCOD977-11|Bembidion tibiale|BC ZSM COL 03257
3
FBCOD732-11|Bembidion tibiale|BC ZSM COL 02537
30
36
GBCOU5463-14|Bembidion tibiale|GBOL05283
FBCOD679-11|Bembidion geniculatum|BC ZSM COL 02484
74
FBCOK321-13|Bembidion geniculatum|BFB_Col_FK_9726
42
GBCOU278-13|Bembidion geniculatum|GBOL 01326
99
GBCOU274-13|Bembidion geniculatum|GBOL 01322
98
GBCOC221-12|Bembidion geniculatum|GBOL_Col_FK_0601
86
99
FBCOI108-12|Bembidion geniculatum|BFB_Col_FK_6948
84
FBCOD722-11|Bembidion complanatum|BC ZSM COL 02527
GBCOU4064-13|Bembidion complanatum|GBOL_Col_FK_9004
99
GBCOU2563-13|Bembidion complanatum|GBOL_Col_FK_7218
27
GBCOU271-13|Bembidion complanatum|GBOL 01319
FBCOD676-11|Bembidion complanatum|BC ZSM COL 02481
11
FBCOD677-11|Bembidion complanatum|BC ZSM COL 02482
FBCOD723-11|Bembidion complanatum|BC ZSM COL 02528
14
GBCOU267-13|Bembidion complanatum|GBOL 01315
GBCOU268-13|Bembidion complanatum|GBOL 01316
18
GBCOU270-13|Bembidion complanatum|GBOL 01318
GBCOU273-13|Bembidion complanatum|GBOL 01321
GBCOU1238-13|Bembidion complanatum|GBOL_Col_FK_6368
GBCOU1239-13|Bembidion complanatum|GBOL_Col_FK_6369
GBCOU2562-13|Bembidion complanatum|GBOL_Col_FK_7217
FBCOD972-11|Bembidion monticola|BC ZSM COL 03252

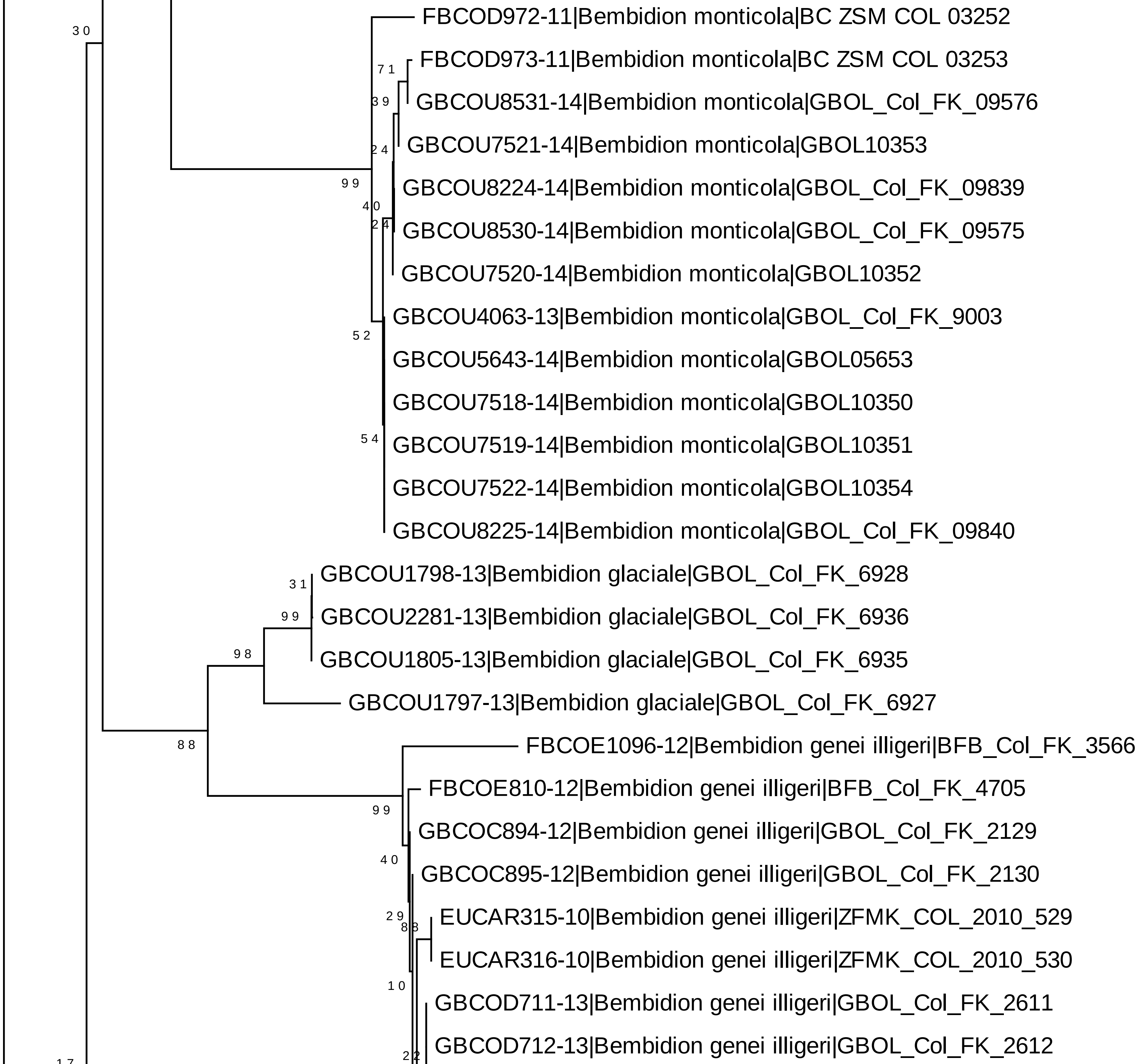
FBCOD972-11|Bembidion monticola|BC ZSM COL 03252
FBCOD973-11|Bembidion monticola|BC ZSM COL 03253
GBCOU8531-14|Bembidion monticola|GBOL_Col_FK_09576
GBCOU7521-14|Bembidion monticola|GBOL10353
GBCOU8224-14|Bembidion monticola|GBOL_Col_FK_09839
GBCOU8530-14|Bembidion monticola|GBOL_Col_FK_09575
GBCOU7520-14|Bembidion monticola|GBOL10352
GBCOU4063-13|Bembidion monticola|GBOL_Col_FK_9003
GBCOU5643-14|Bembidion monticola|GBOL05653
GBCOU7518-14|Bembidion monticola|GBOL10350
GBCOU7519-14|Bembidion monticola|GBOL10351
GBCOU7522-14|Bembidion monticola|GBOL10354
GBCOU8225-14|Bembidion monticola|GBOL_Col_FK_09840
GBCOU1798-13|Bembidion glaciale|GBOL_Col_FK_6928
GBCOU2281-13|Bembidion glaciale|GBOL_Col_FK_6936
GBCOU1805-13|Bembidion glaciale|GBOL_Col_FK_6935
GBCOU1797-13|Bembidion glaciale|GBOL_Col_FK_6927
FBCOE1096-12|Bembidion genei illigeri|BFB_Col_FK_3566
FBCOE810-12|Bembidion genei illigeri|BFB_Col_FK_4705
GBCOC894-12|Bembidion genei illigeri|GBOL_Col_FK_2129
GBCOC895-12|Bembidion genei illigeri|GBOL_Col_FK_2130
EUCAR315-10|Bembidion genei illigeri|ZFMK_COL_2010_529
EUCAR316-10|Bembidion genei illigeri|ZFMK_COL_2010_530
GBCOD711-13|Bembidion genei illigeri|GBOL_Col_FK_2611
GBCOD712-13|Bembidion genei illigeri|GBOL_Col_FK_2612

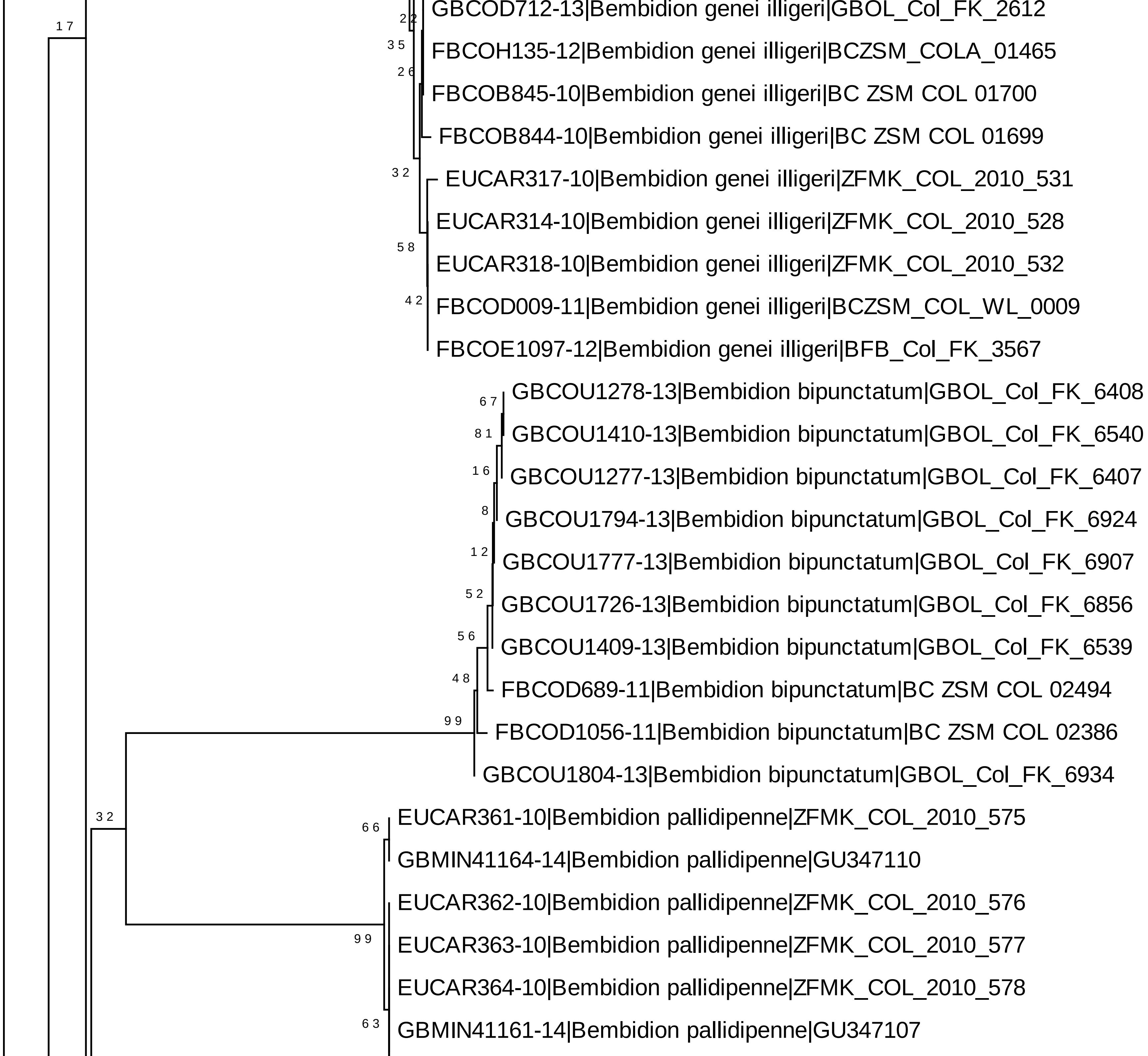
GBCOD712-13|Bembidion genei illigeri|GBOL_Col_FK_2612
FBCOH135-12|Bembidion genei illigeri|BCZSM_COLA_01465
FBCOB845-10|Bembidion genei illigeri|BC ZSM COL 01700
FBCOB844-10|Bembidion genei illigeri|BC ZSM COL 01699
EUCAR317-10|Bembidion genei illigeri|ZFMK_COL_2010_531
EUCAR314-10|Bembidion genei illigeri|ZFMK_COL_2010_528
EUCAR318-10|Bembidion genei illigeri|ZFMK_COL_2010_532
FBCOD009-11|Bembidion genei illigeri|BCZSM_COL_WL_0009
FBCOE1097-12|Bembidion genei illigeri|BFB_Col_FK_3567
GBCOU1278-13|Bembidion bipunctatum|GBOL_Col_FK_6408
GBCOU1410-13|Bembidion bipunctatum|GBOL_Col_FK_6540
GBCOU1277-13|Bembidion bipunctatum|GBOL_Col_FK_6407
GBCOU1794-13|Bembidion bipunctatum|GBOL_Col_FK_6924
GBCOU1777-13|Bembidion bipunctatum|GBOL_Col_FK_6907
GBCOU1726-13|Bembidion bipunctatum|GBOL_Col_FK_6856
GBCOU1409-13|Bembidion bipunctatum|GBOL_Col_FK_6539
FBCOD689-11|Bembidion bipunctatum|BC ZSM COL 02494
FBCOD1056-11|Bembidion bipunctatum|BC ZSM COL 02386
GBCOU1804-13|Bembidion bipunctatum|GBOL_Col_FK_6934
EUCAR361-10|Bembidion pallidipenne|ZFMK_COL_2010_575
GBMIN41164-14|Bembidion pallidipenne|GU347110
EUCAR362-10|Bembidion pallidipenne|ZFMK_COL_2010_576
EUCAR363-10|Bembidion pallidipenne|ZFMK_COL_2010_577
EUCAR364-10|Bembidion pallidipenne|ZFMK_COL_2010_578
GBMIN41161-14|Bembidion pallidipenne|GU347107

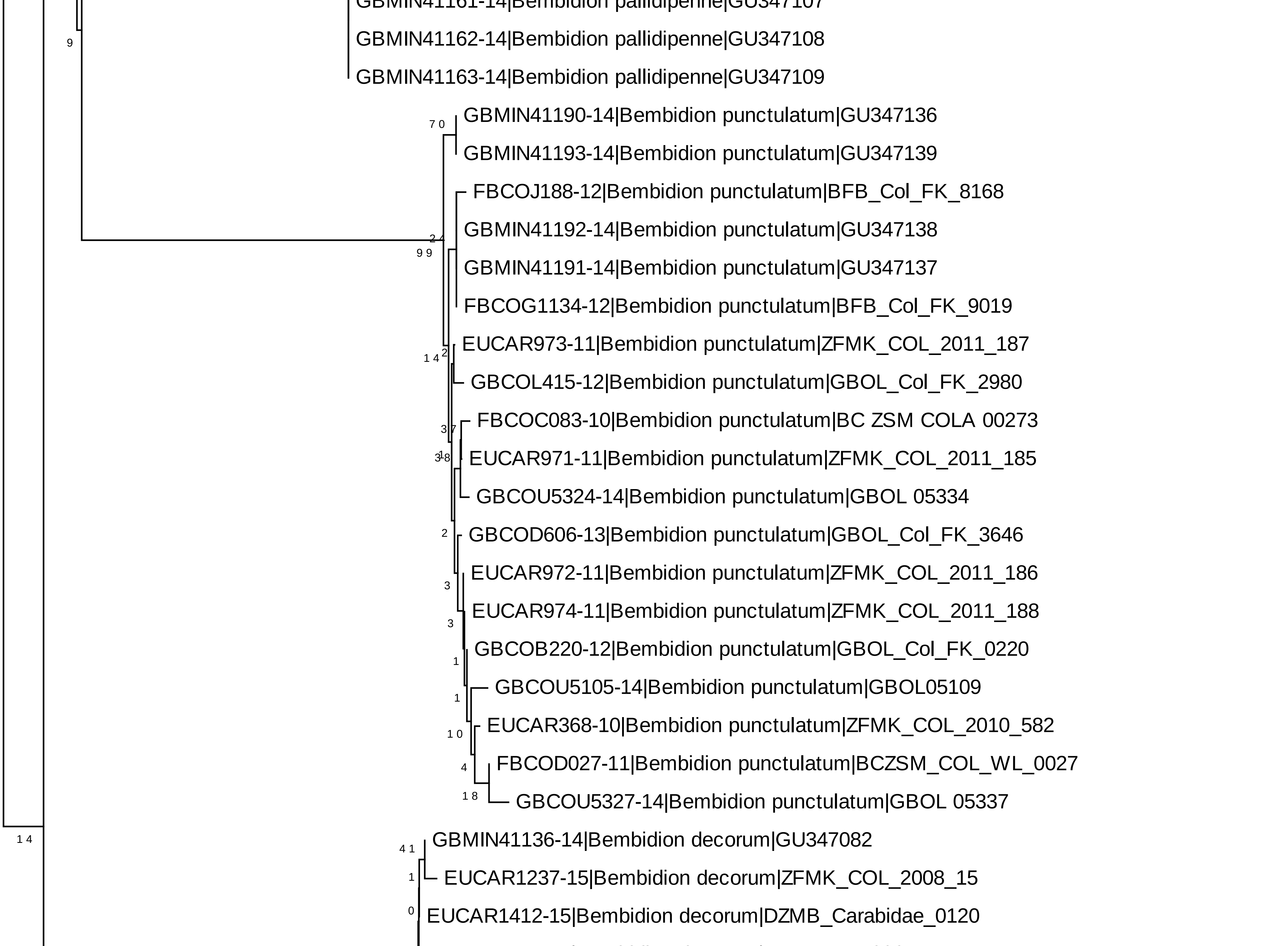
GBMIN41161-14|Bembidion pallidipenne|GU347107
GBMIN41162-14|Bembidion pallidipenne|GU347108
GBMIN41163-14|Bembidion pallidipenne|GU347109
GBMIN41190-14|Bembidion punctulatum|GU347136
GBMIN41193-14|Bembidion punctulatum|GU347139
FBCOJ188-12|Bembidion punctulatum|BFB_Col_FK_8168
GBMIN41192-14|Bembidion punctulatum|GU347138
GBMIN41191-14|Bembidion punctulatum|GU347137
FBCOG1134-12|Bembidion punctulatum|BFB_Col_FK_9019
EUCAR973-11|Bembidion punctulatum|ZFMK_COL_2011_187
GBCOL415-12|Bembidion punctulatum|GBOL_Col_FK_2980
FBCOC083-10|Bembidion punctulatum|BC ZSM COLA 00273
EUCAR971-11|Bembidion punctulatum|ZFMK_COL_2011_185
GBCOU5324-14|Bembidion punctulatum|GBOL 05334
GBCOD606-13|Bembidion punctulatum|GBOL_Col_FK_3646
EUCAR972-11|Bembidion punctulatum|ZFMK_COL_2011_186
EUCAR974-11|Bembidion punctulatum|ZFMK_COL_2011_188
GBCOB220-12|Bembidion punctulatum|GBOL_Col_FK_0220
GBCOU5105-14|Bembidion punctulatum|GBOL05109
EUCAR368-10|Bembidion punctulatum|ZFMK_COL_2010_582
FBCOD027-11|Bembidion punctulatum|BCZSM_COL_WL_0027
GBCOU5327-14|Bembidion punctulatum|GBOL 05337
GBMIN41136-14|Bembidion decorum|GU347082
EUCAR1237-15|Bembidion decorum|ZFMK_COL_2008_15
EUCAR1412-15|Bembidion decorum|DZMB_Carabidae_0120

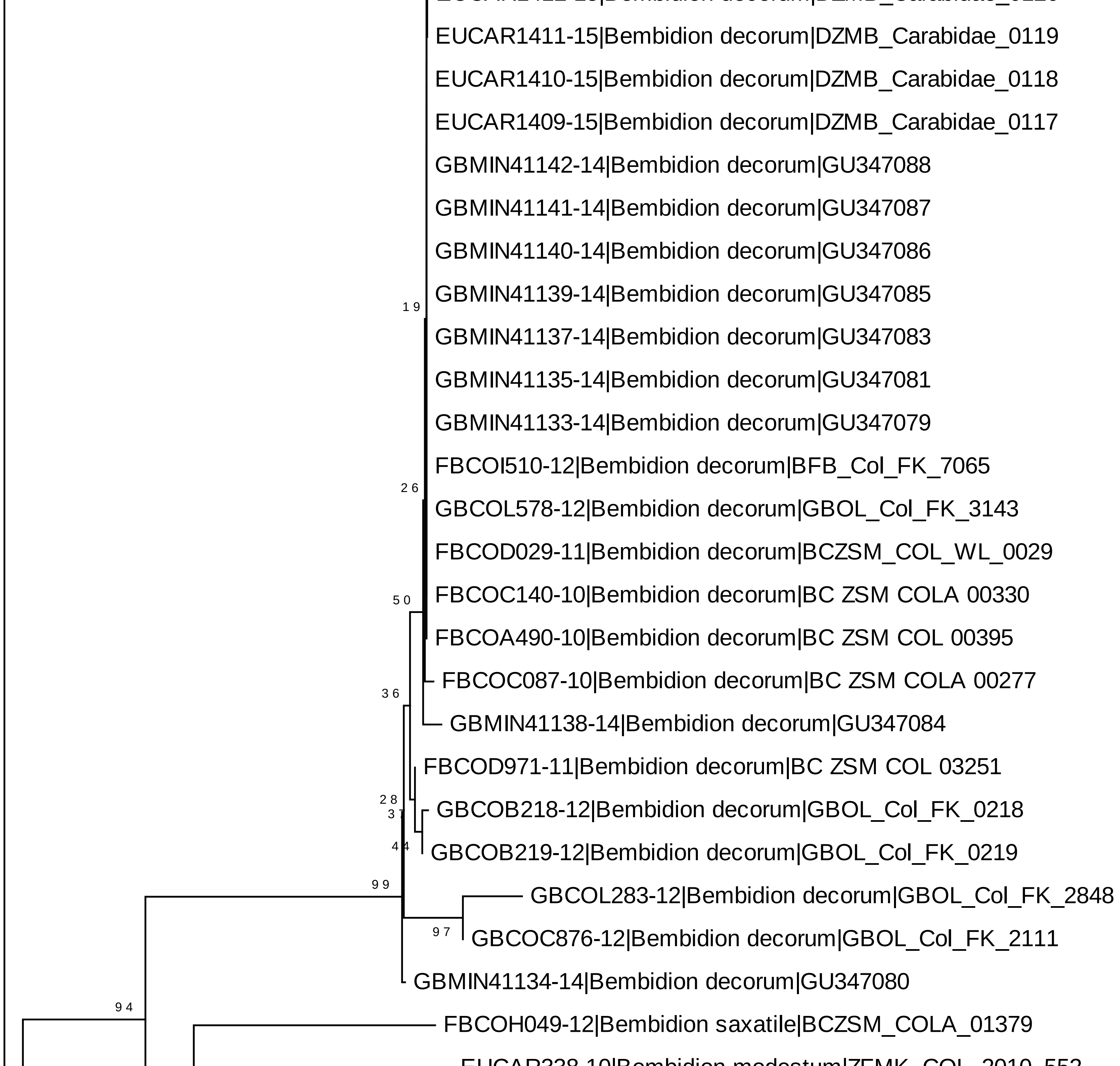
EUCAR1411-15|Bembidion decorum|DZMB_Carabidae_0119
EUCAR1410-15|Bembidion decorum|DZMB_Carabidae_0118
EUCAR1409-15|Bembidion decorum|DZMB_Carabidae_0117
GBMIN41142-14|Bembidion decorum|GU347088
GBMIN41141-14|Bembidion decorum|GU347087
GBMIN41140-14|Bembidion decorum|GU347086
GBMIN41139-14|Bembidion decorum|GU347085
19
GBMIN41137-14|Bembidion decorum|GU347083
GBMIN41135-14|Bembidion decorum|GU347081
GBMIN41133-14|Bembidion decorum|GU347079
FBCOI510-12|Bembidion decorum|BFB_Col_FK_7065
26
GBCOL578-12|Bembidion decorum|GBOL_Col_FK_3143
FBCOD029-11|Bembidion decorum|BCZSM_COL_WL_0029
50
FBCOC140-10|Bembidion decorum|BC ZSM COLA 00330
FBCOA490-10|Bembidion decorum|BC ZSM COL 00395
FBCOC087-10|Bembidion decorum|BC ZSM COLA 00277
36
GBMIN41138-14|Bembidion decorum|GU347084
FBCOD971-11|Bembidion decorum|BC ZSM COL 03251
28
GBCOB218-12|Bembidion decorum|GBOL_Col_FK_0218
44
GBCOB219-12|Bembidion decorum|GBOL_Col_FK_0219
99
GBCOL283-12|Bembidion decorum|GBOL_Col_FK_2848
97
GBCOC876-12|Bembidion decorum|GBOL_Col_FK_2111
GBMIN41134-14|Bembidion decorum|GU347080
94
FBCOH049-12|Bembidion saxatile|BCZSM_COLA_01379

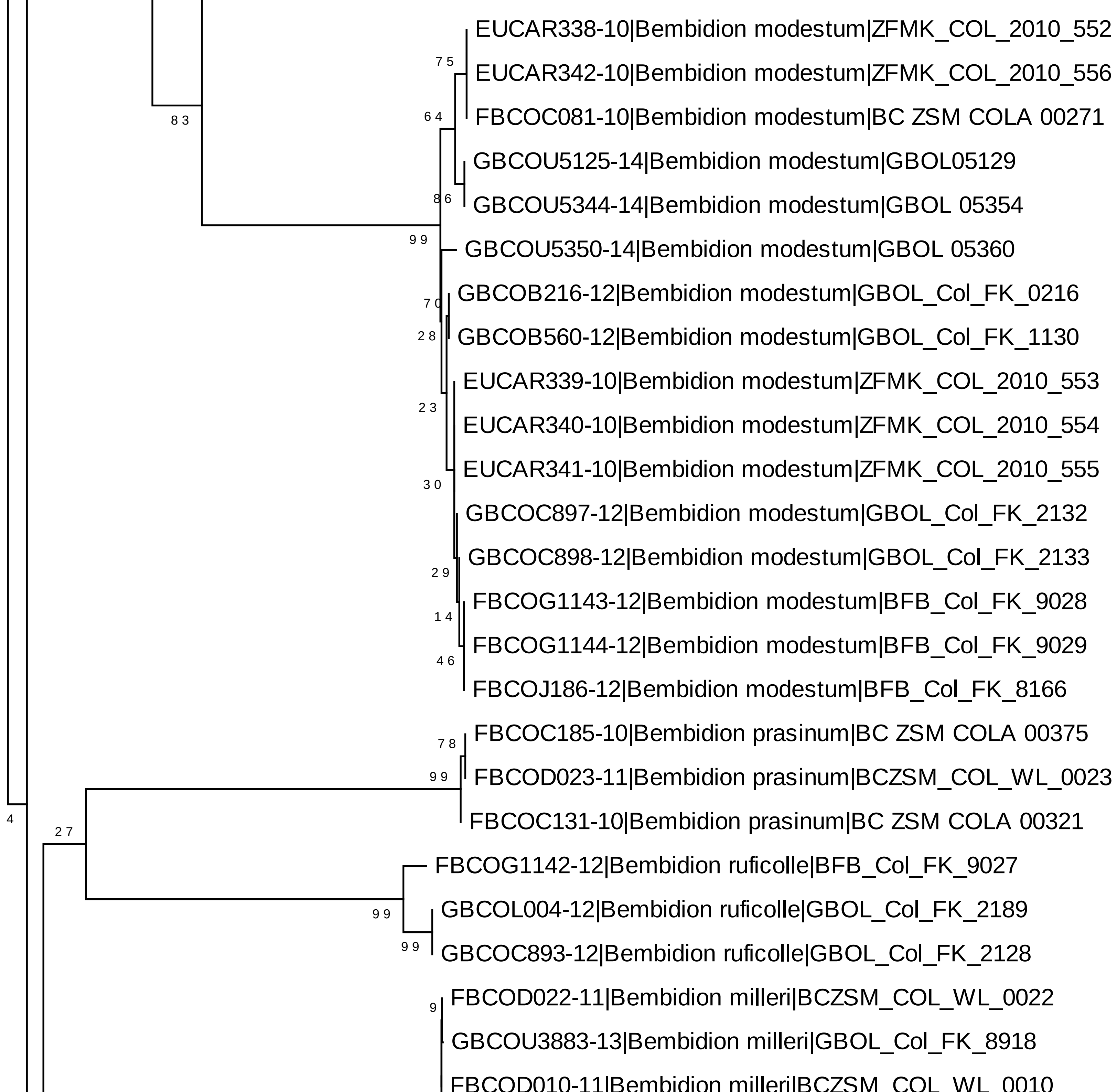

EUCAR338-10|Bembidion modestum|ZFMK_COL_2010_552
75
EUCAR342-10|Bembidion modestum|ZFMK_COL_2010_556
83
64
FBCOC081-10|Bembidion modestum|BC ZSM COLA 00271
GBCOU5125-14|Bembidion modestum|GBOL05129
86
GBCOU5344-14|Bembidion modestum|GBOL 05354
99
GBCOU5350-14|Bembidion modestum|GBOL 05360
GBCOB216-12|Bembidion modestum|GBOL_Col_FK_0216
70
28
GBCOB560-12|Bembidion modestum|GBOL_Col_FK_1130
EUCAR339-10|Bembidion modestum|ZFMK_COL_2010_553
23
EUCAR340-10|Bembidion modestum|ZFMK_COL_2010_554
EUCAR341-10|Bembidion modestum|ZFMK_COL_2010_555
30
GBCOC897-12|Bembidion modestum|GBOL_Col_FK_2132
GBCOC898-12|Bembidion modestum|GBOL_Col_FK_2133
29
FBCOG1143-12|Bembidion modestum|BFB_Col_FK_9028
14
FBCOG1144-12|Bembidion modestum|BFB_Col_FK_9029
46
FBCOJ186-12|Bembidion modestum|BFB_Col_FK_8166
FBCOC185-10|Bembidion prasinum|BC ZSM COLA 00375
78
99
FBCOD023-11|Bembidion prasinum|BCZSM_COL_WL_0023
4
FBCOC131-10|Bembidion prasinum|BC ZSM COLA 00321
27
FBCOG1142-12|Bembidion ruficolle|BFB_Col_FK_9027
GBCOL004-12|Bembidion ruficolle|GBOL_Col_FK_2189
99
99
GBCOC893-12|Bembidion ruficolle|GBOL_Col_FK_2128
FBCOD022-11|Bembidion milleri|BCZSM_COL_WL_0022
9
GBCOU3883-13|Bembidion milleri|GBOL_Col_FK_8918
FBCOD010-11|Bembidion milleri|BCZSM_COL_WL_0010

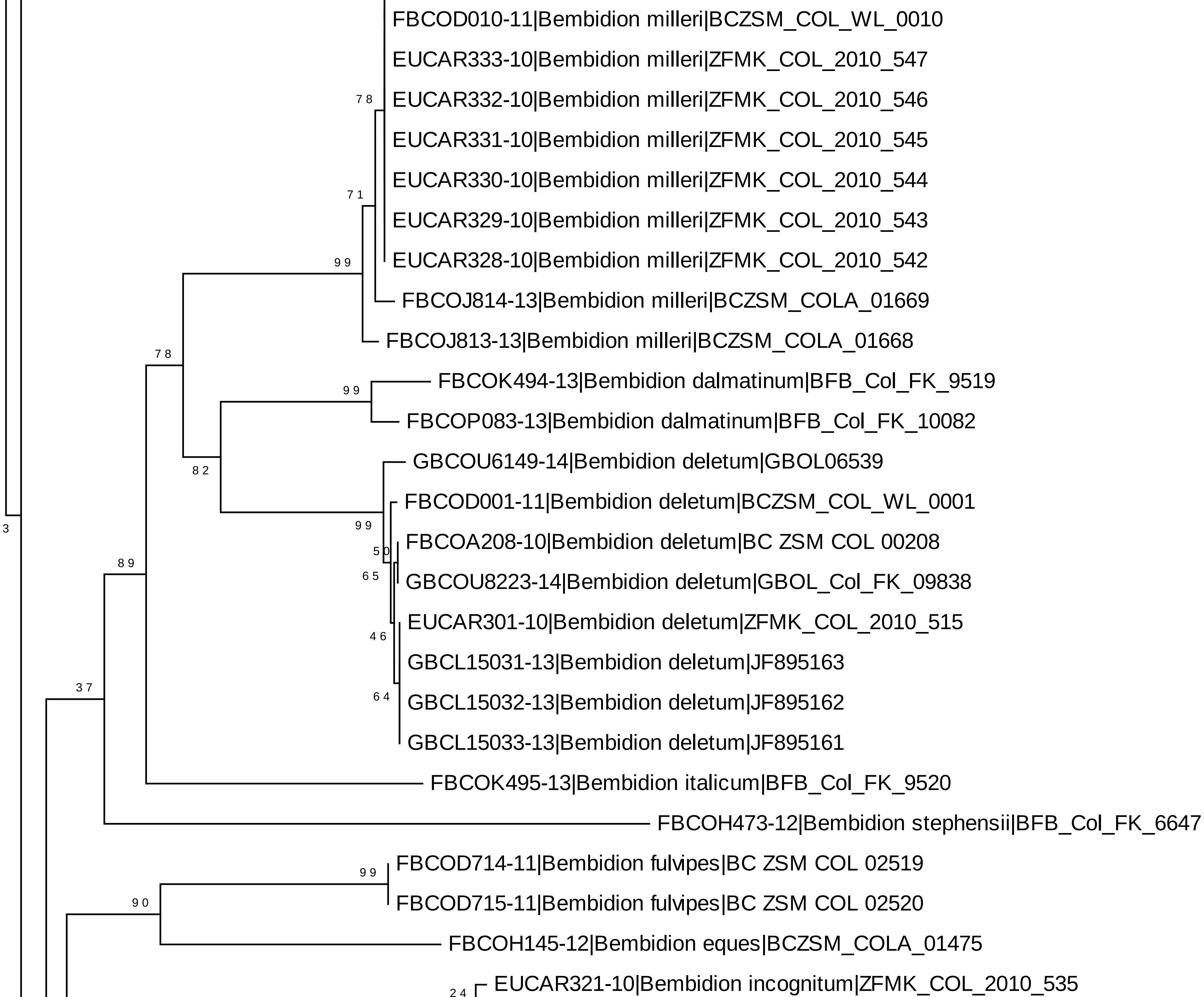
FBCOD010-11|Bembidion milleri|BCZSM_COL_WL_0010
EUCAR333-10|Bembidion milleri|ZFMK_COL_2010_547
78
EUCAR332-10|Bembidion milleri|ZFMK_COL_2010_546
EUCAR331-10|Bembidion milleri|ZFMK_COL_2010_545
71
EUCAR330-10|Bembidion milleri|ZFMK_COL_2010_544
EUCAR329-10|Bembidion milleri|ZFMK_COL_2010_543
99
EUCAR328-10|Bembidion milleri|ZFMK_COL_2010_542
FBCOJ814-13|Bembidion milleri|BCZSM_COLA_01669
78
FBCOJ813-13|Bembidion milleri|BCZSM_COLA_01668
99
FBCOK494-13|Bembidion dalmatinum|BFB_Col_FK_9519
FBCOP083-13|Bembidion dalmatinum|BFB_Col_FK_10082
GBCOU6149-14|Bembidion deletum|GBOL06539
82
FBCOD001-11|Bembidion deletum|BCZSM_COL_WL_0001
3
99
FBCOA208-10|Bembidion deletum|BC ZSM COL 00208
50
89
65
GBCOU8223-14|Bembidion deletum|GBOL_Col_FK_09838
EUCAR301-10|Bembidion deletum|ZFMK_COL_2010_515
46
GBCL15031-13|Bembidion deletum|JF895163
37
64
GBCL15032-13|Bembidion deletum|JF895162
GBCL15033-13|Bembidion deletum|JF895161
FBCOK495-13|Bembidion italicum|BFB_Col_FK_9520
FBCOH473-12|Bembidion stephensii|BFB_Col_FK_6647
99
FBCOD714-11|Bembidion fulvipes|BC ZSM COL 02519
90
FBCOD715-11|Bembidion fulvipes|BC ZSM COL 02520
FBCOH145-12|Bembidion eques|BCZSM_COLA_01475
24
EUCAR321-10|Bembidion incognitum|ZFMK_COL_2010_535

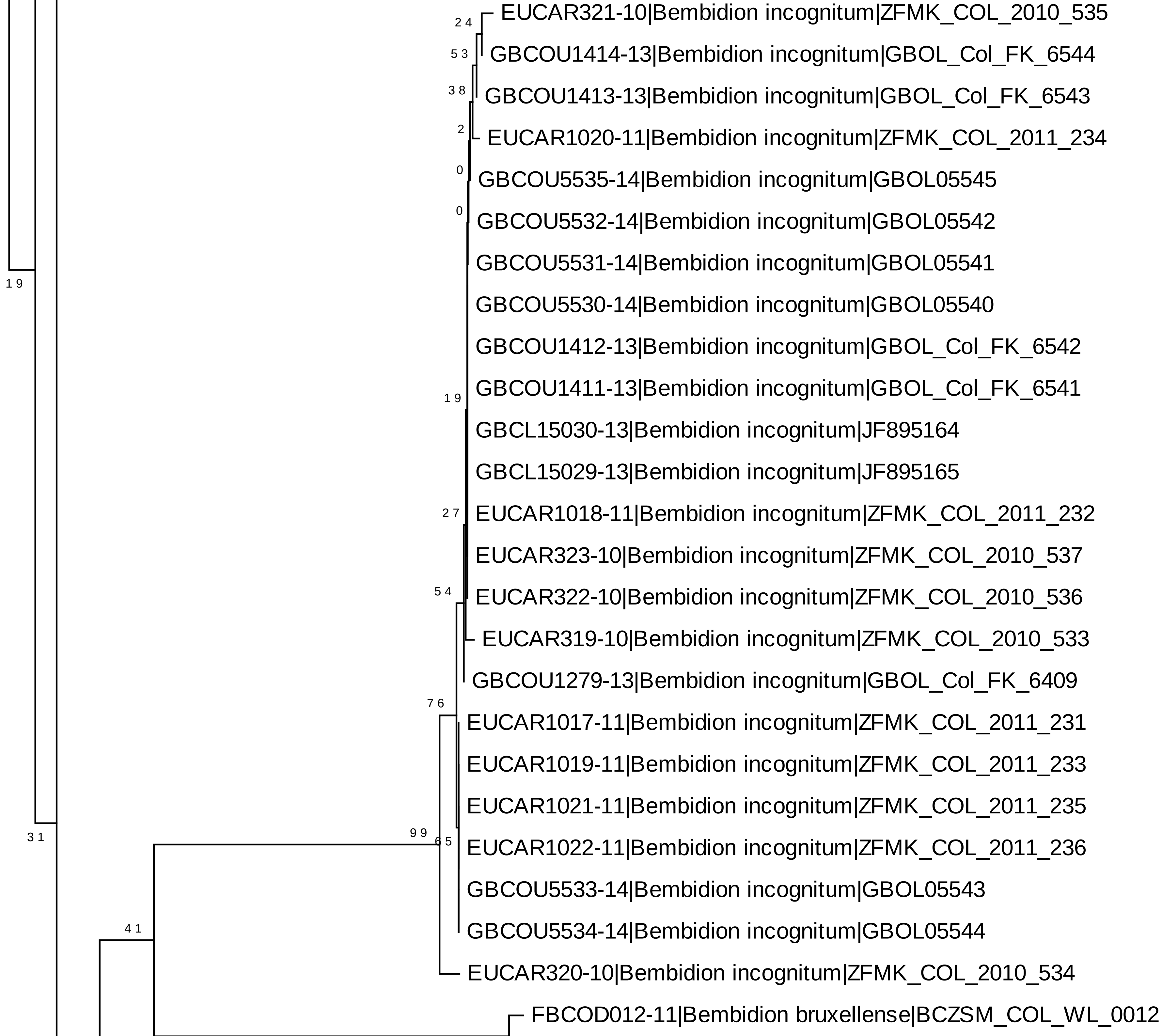
EUCAR321-10|Bembidion incognitum|ZFMK_COL_2010_535
GBCOU1414-13|Bembidion incognitum|GBOL_Col_FK_6544
GBCOU1413-13|Bembidion incognitum|GBOL_Col_FK_6543
EUCAR1020-11|Bembidion incognitum|ZFMK_COL_2011_234
GBCOU5535-14|Bembidion incognitum|GBOL05545
GBCOU5532-14|Bembidion incognitum|GBOL05542
GBCOU5531-14|Bembidion incognitum|GBOL05541
GBCOU5530-14|Bembidion incognitum|GBOL05540
GBCOU1412-13|Bembidion incognitum|GBOL_Col_FK_6542
GBCOU1411-13|Bembidion incognitum|GBOL_Col_FK_6541
GBCL15030-13|Bembidion incognitum|JF895164
GBCL15029-13|Bembidion incognitum|JF895165
EUCAR1018-11|Bembidion incognitum|ZFMK_COL_2011_232
EUCAR323-10|Bembidion incognitum|ZFMK_COL_2010_537
EUCAR322-10|Bembidion incognitum|ZFMK_COL_2010_536
EUCAR319-10|Bembidion incognitum|ZFMK_COL_2010_533
GBCOU1279-13|Bembidion incognitum|GBOL_Col_FK_6409
EUCAR1017-11|Bembidion incognitum|ZFMK_COL_2011_231
EUCAR1019-11|Bembidion incognitum|ZFMK_COL_2011_233
EUCAR1021-11|Bembidion incognitum|ZFMK_COL_2011_235
EUCAR1022-11|Bembidion incognitum|ZFMK_COL_2011_236
GBCOU5533-14|Bembidion incognitum|GBOL05543
GBCOU5534-14|Bembidion incognitum|GBOL05544
EUCAR320-10|Bembidion incognitum|ZFMK_COL_2010_534
FBCOD012-11|Bembidion bruxellense|BCZSM_COL_WL_0012
24
53
38
2
0
0
19
19
27
54
76
99
65
31
41

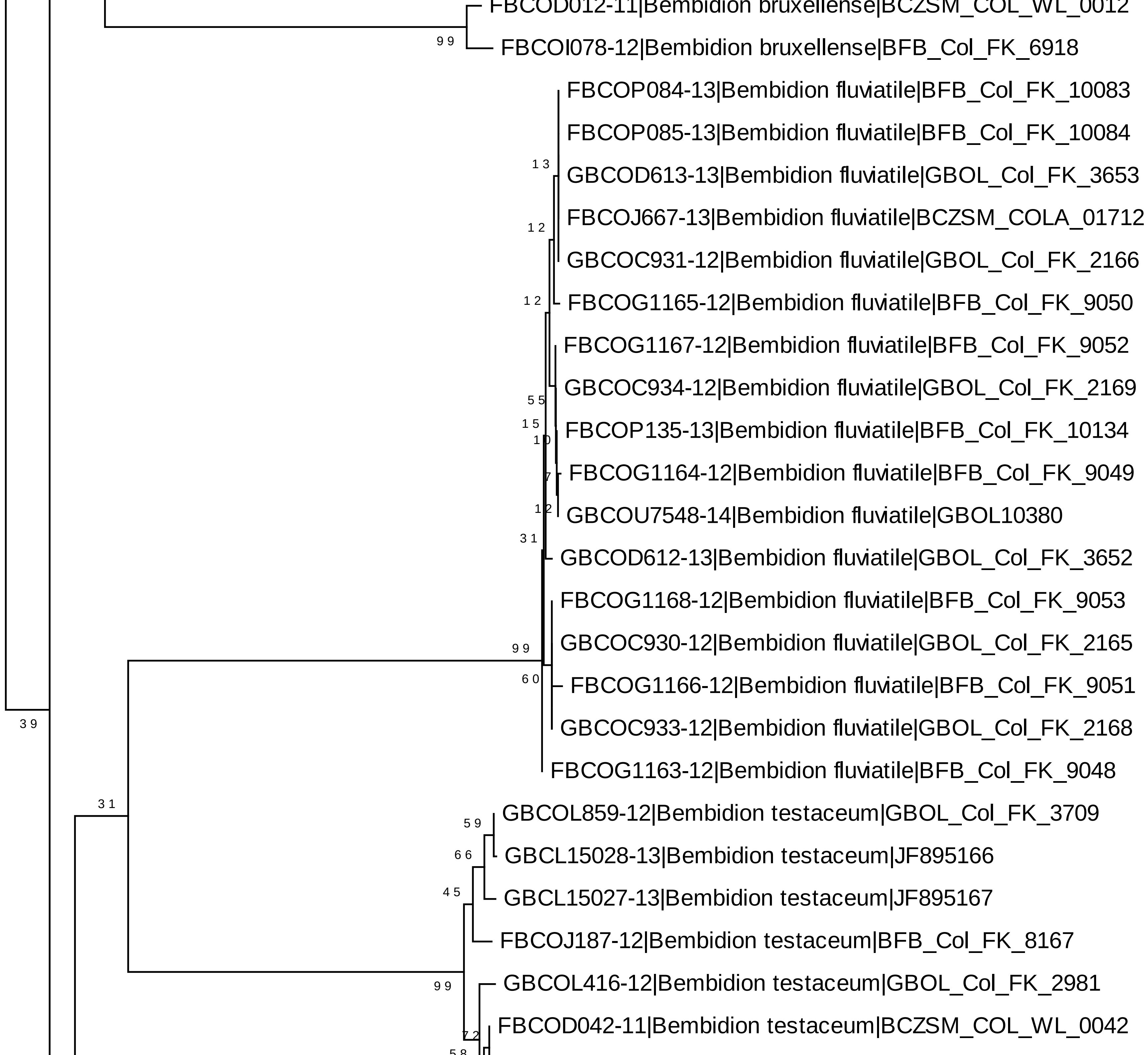
FBCOD012-11|Bembidion bruxellense|BCZSM_COL_WL_0012
99
FBCOI078-12|Bembidion bruxellense|BFB_Col_FK_6918
FBCOP084-13|Bembidion fluviatile|BFB_Col_FK_10083
FBCOP085-13|Bembidion fluviatile|BFB_Col_FK_10084
13
GBCOD613-13|Bembidion fluviatile|GBOL_Col_FK_3653
FBCOJ667-13|Bembidion fluviatile|BCZSM_COLA_01712
12
GBCOC931-12|Bembidion fluviatile|GBOL_Col_FK_2166
12
FBCOG1165-12|Bembidion fluviatile|BFB_Col_FK_9050
FBCOG1167-12|Bembidion fluviatile|BFB_Col_FK_9052
55
GBCOC934-12|Bembidion fluviatile|GBOL_Col_FK_2169
15
FBCOP135-13|Bembidion fluviatile|BFB_Col_FK_10134
10
7
FBCOG1164-12|Bembidion fluviatile|BFB_Col_FK_9049
12
GBCOU7548-14|Bembidion fluviatile|GBOL10380
31
GBCOD612-13|Bembidion fluviatile|GBOL_Col_FK_3652
FBCOG1168-12|Bembidion fluviatile|BFB_Col_FK_9053
99
GBCOC930-12|Bembidion fluviatile|GBOL_Col_FK_2165
60
FBCOG1166-12|Bembidion fluviatile|BFB_Col_FK_9051
39
GBCOC933-12|Bembidion fluviatile|GBOL_Col_FK_2168
FBCOG1163-12|Bembidion fluviatile|BFB_Col_FK_9048
31
59
GBCOL859-12|Bembidion testaceum|GBOL_Col_FK_3709
66
GBCL15028-13|Bembidion testaceum|JF895166
45
GBCL15027-13|Bembidion testaceum|JF895167
FBCOJ187-12|Bembidion testaceum|BFB_Col_FK_8167
99
GBCOL416-12|Bembidion testaceum|GBOL_Col_FK_2981
72
FBCOD042-11|Bembidion testaceum|BCZSM_COL_WL_0042
58

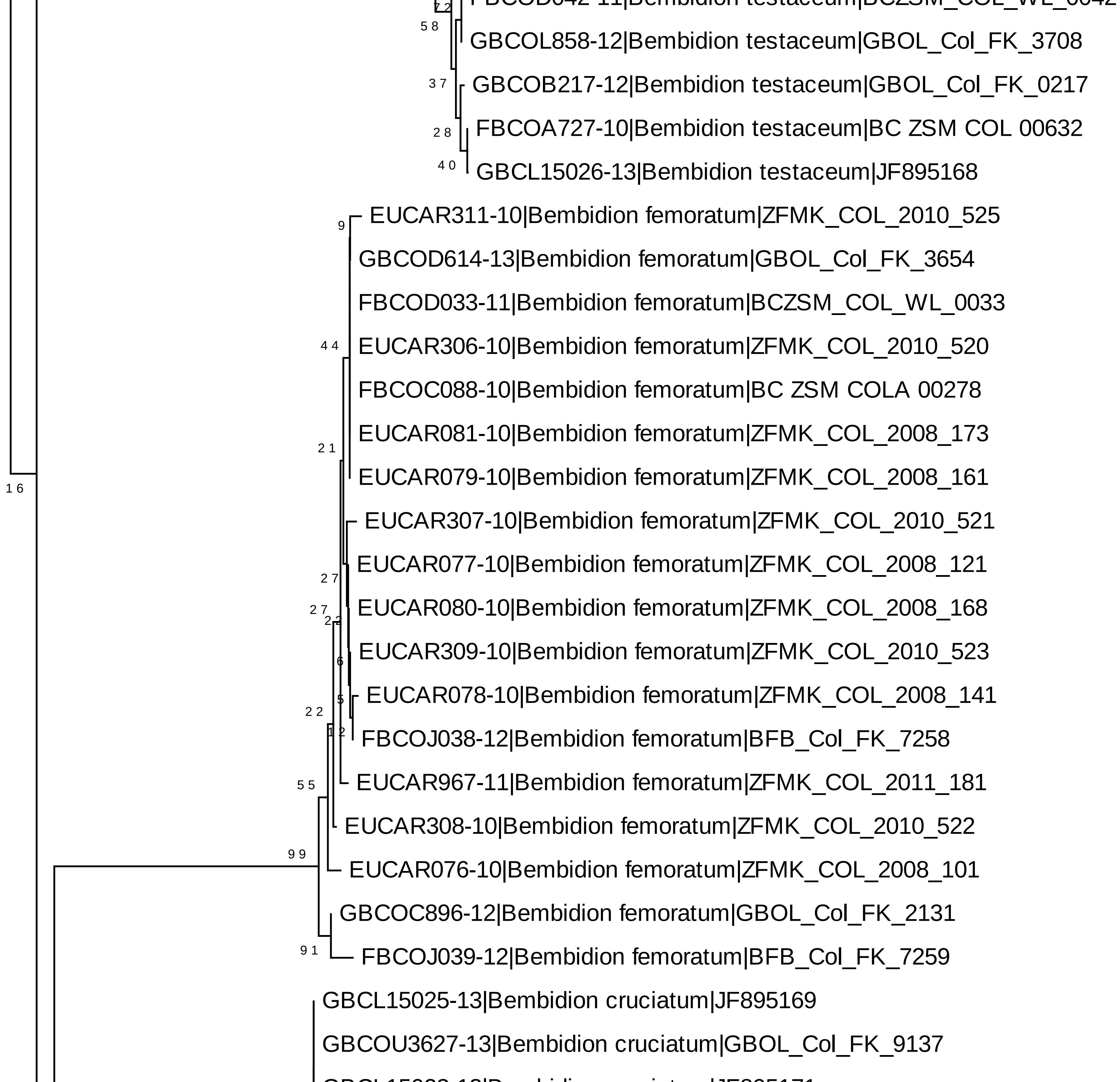
GBCOL858-12|Bembidion testaceum|GBOL_Col_FK_3708
GBCOB217-12|Bembidion testaceum|GBOL_Col_FK_0217
FBCOA727-10|Bembidion testaceum|BC ZSM COL 00632
GBCL15026-13|Bembidion testaceum|JF895168
EUCAR311-10|Bembidion femoratum|ZFMK_COL_2010_525
GBCOD614-13|Bembidion femoratum|GBOL_Col_FK_3654
FBCOD033-11|Bembidion femoratum|BCZSM_COL_WL_0033
EUCAR306-10|Bembidion femoratum|ZFMK_COL_2010_520
FBCOC088-10|Bembidion femoratum|BC ZSM COLA 00278
EUCAR081-10|Bembidion femoratum|ZFMK_COL_2008_173
EUCAR079-10|Bembidion femoratum|ZFMK_COL_2008_161
EUCAR307-10|Bembidion femoratum|ZFMK_COL_2010_521
EUCAR077-10|Bembidion femoratum|ZFMK_COL_2008_121
EUCAR080-10|Bembidion femoratum|ZFMK_COL_2008_168
EUCAR309-10|Bembidion femoratum|ZFMK_COL_2010_523
EUCAR078-10|Bembidion femoratum|ZFMK_COL_2008_141
FBCOJ038-12|Bembidion femoratum|BFB_Col_FK_7258
EUCAR967-11|Bembidion femoratum|ZFMK_COL_2011_181
EUCAR308-10|Bembidion femoratum|ZFMK_COL_2010_522
EUCAR076-10|Bembidion femoratum|ZFMK_COL_2008_101
GBCOC896-12|Bembidion femoratum|GBOL_Col_FK_2131
FBCOJ039-12|Bembidion femoratum|BFB_Col_FK_7259
GBCL15025-13|Bembidion cruciatum|JF895169
GBCOU3627-13|Bembidion cruciatum|GBOL_Col_FK_9137

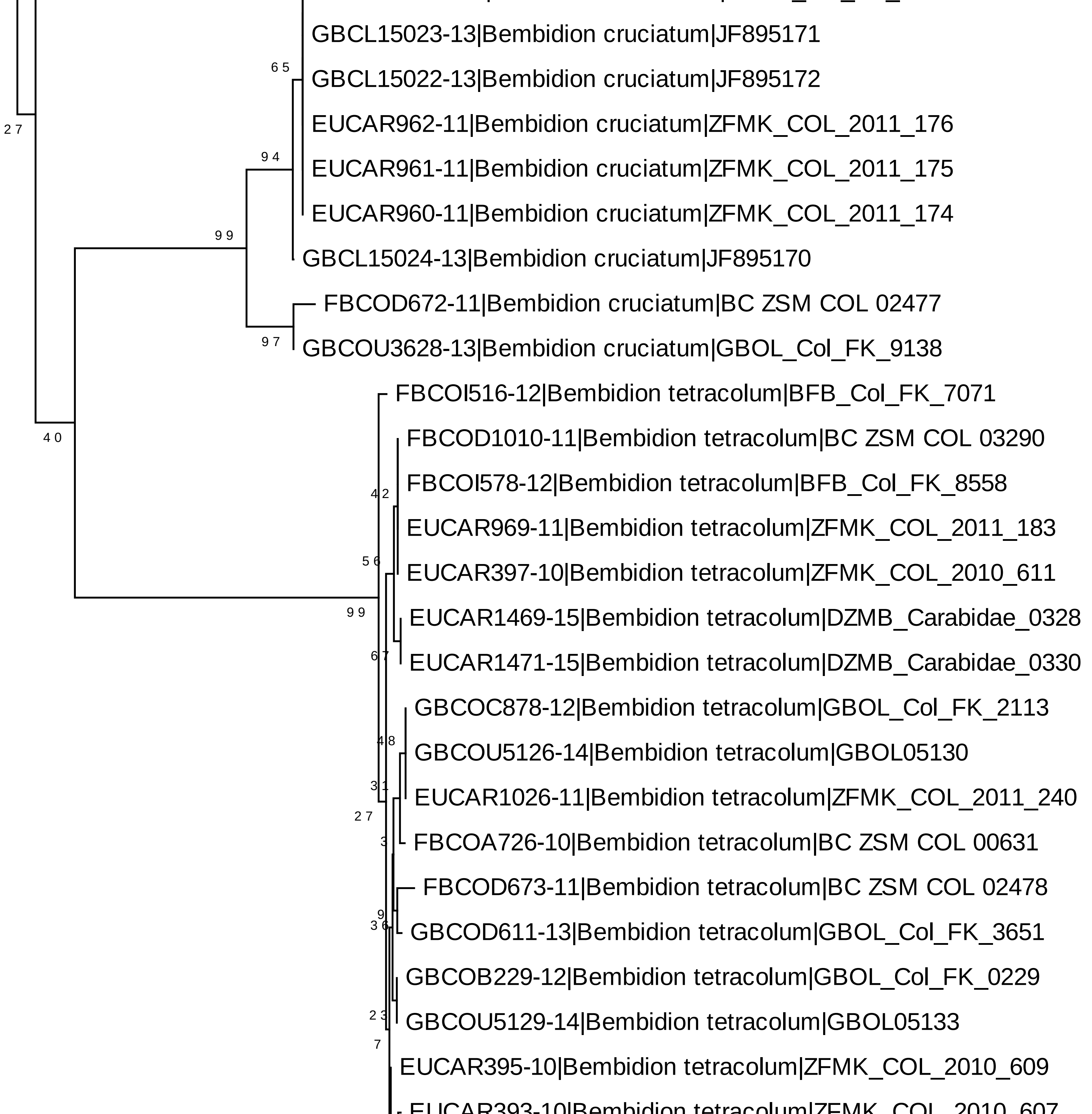
GBCL15023-13|Bembidion cruciatum|JF895171
GBCL15022-13|Bembidion cruciatum|JF895172
EUCAR962-11|Bembidion cruciatum|ZFMK_COL_2011_176
EUCAR961-11|Bembidion cruciatum|ZFMK_COL_2011_175
EUCAR960-11|Bembidion cruciatum|ZFMK_COL_2011_174
GBCL15024-13|Bembidion cruciatum|JF895170
FBCOD672-11|Bembidion cruciatum|BC ZSM COL 02477
GBCOU3628-13|Bembidion cruciatum|GBOL_Col_FK_9138
FBCOI516-12|Bembidion tetracolum|BFB_Col_FK_7071
FBCOD1010-11|Bembidion tetracolum|BC ZSM COL 03290
FBCOI578-12|Bembidion tetracolum|BFB_Col_FK_8558
EUCAR969-11|Bembidion tetracolum|ZFMK_COL_2011_183
EUCAR397-10|Bembidion tetracolum|ZFMK_COL_2010_611
EUCAR1469-15|Bembidion tetracolum|DZMB_Carabidae_0328
EUCAR1471-15|Bembidion tetracolum|DZMB_Carabidae_0330
GBCOC878-12|Bembidion tetracolum|GBOL_Col_FK_2113
GBCOU5126-14|Bembidion tetracolum|GBOL05130
EUCAR1026-11|Bembidion tetracolum|ZFMK_COL_2011_240
FBCOA726-10|Bembidion tetracolum|BC ZSM COL 00631
FBCOD673-11|Bembidion tetracolum|BC ZSM COL 02478
GBCOD611-13|Bembidion tetracolum|GBOL_Col_FK_3651
GBCOB229-12|Bembidion tetracolum|GBOL_Col_FK_0229
GBCOU5129-14|Bembidion tetracolum|GBOL05133
EUCAR395-10|Bembidion tetracolum|ZFMK_COL_2010_609

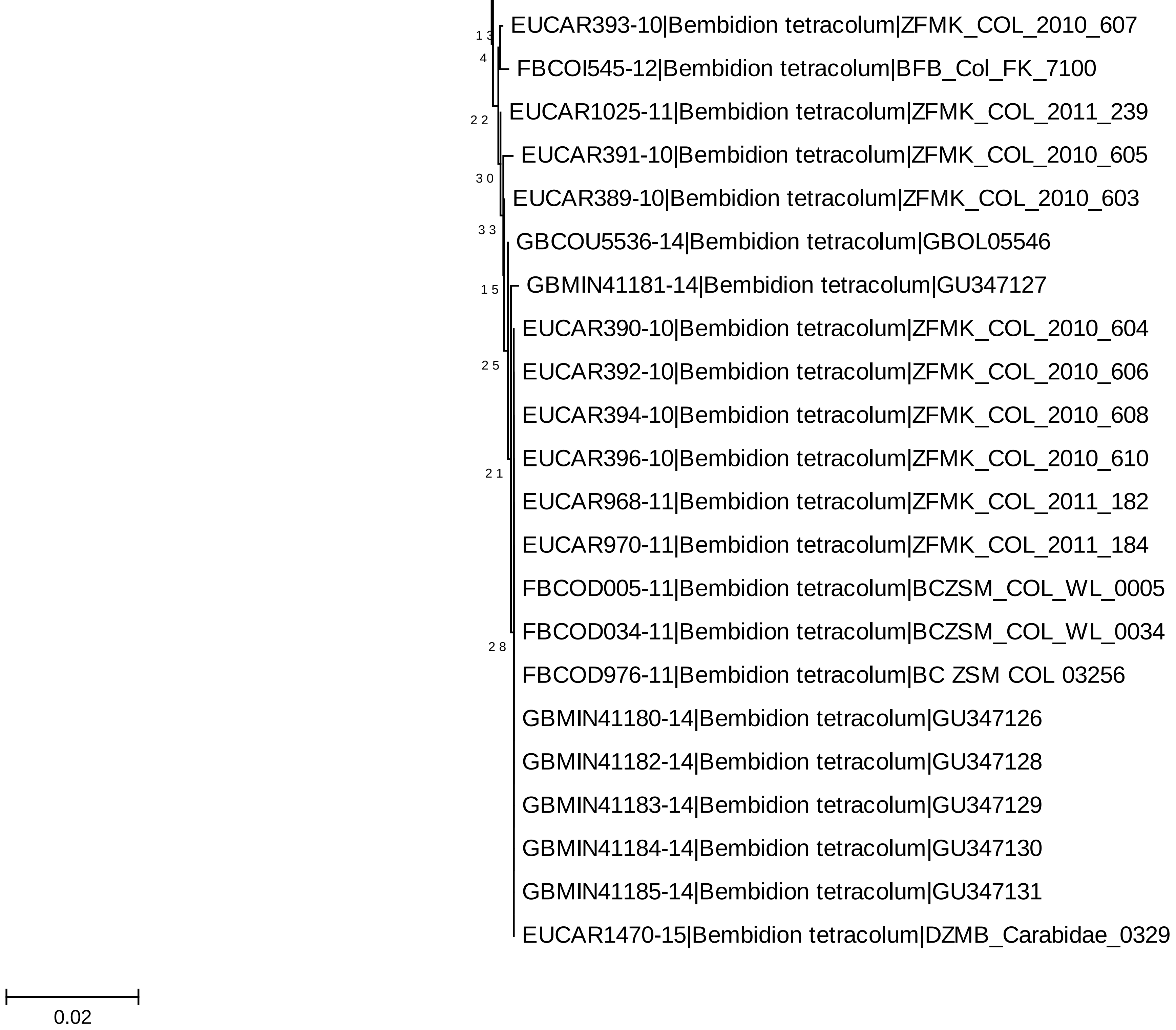
EUCAR393-10|Bembidion tetracolum|ZFMK_COL_2010_607
FBCOI545-12|Bembidion tetracolum|BFB_Col_FK_7100
EUCAR1025-11|Bembidion tetracolum|ZFMK_COL_2011_239
EUCAR391-10|Bembidion tetracolum|ZFMK_COL_2010_605
EUCAR389-10|Bembidion tetracolum|ZFMK_COL_2010_603
GBCOU5536-14|Bembidion tetracolum|GBOL05546
GBMIN41181-14|Bembidion tetracolum|GU347127
EUCAR390-10|Bembidion tetracolum|ZFMK_COL_2010_604
EUCAR392-10|Bembidion tetracolum|ZFMK_COL_2010_606
EUCAR394-10|Bembidion tetracolum|ZFMK_COL_2010_608
EUCAR396-10|Bembidion tetracolum|ZFMK_COL_2010_610
EUCAR968-11|Bembidion tetracolum|ZFMK_COL_2011_182
EUCAR970-11|Bembidion tetracolum|ZFMK_COL_2011_184
FBCOD005-11|Bembidion tetracolum|BCZSM_COL_WL_0005
FBCOD034-11|Bembidion tetracolum|BCZSM_COL_WL_0034
FBCOD976-11|Bembidion tetracolum|BC ZSM COL 03256
GBMIN41180-14|Bembidion tetracolum|GU347126
GBMIN41182-14|Bembidion tetracolum|GU347128
GBMIN41183-14|Bembidion tetracolum|GU347129
GBMIN41184-14|Bembidion tetracolum|GU347130
GBMIN41185-14|Bembidion tetracolum|GU347131
EUCAR1470-15|Bembidion tetracolum|DZMB_Carabidae_0329
13
4
22
30
33
15
25
21
28
0.02
